# From Monomers to Nanocapsules: The Role of Structural Features in Amino-Acid-Derived BTA Self-Assembly

**DOI:** 10.1021/acs.joc.5c01500

**Published:** 2025-10-08

**Authors:** Anna Walczak, Grzegorz Markiewicz, Michał Gliński, Miroslava Čonková, Artur R. Stefankiewicz

**Affiliations:** a Centre for Advanced Technologies, 49562 Adam Mickiewicz University in Poznań, Uniwersytetu Poznańskiego 10, Poznań 61-614, Poland; b Faculty of Chemistry, 49562 Adam Mickiewicz University in Poznań, Uniwersytetu Poznańskiego 8, Poznań 61-614, Poland

## Abstract

The morphology of supramolecular assemblies can be profoundly influenced by even subtle changes in the molecular structure. In this study, we investigate how variations in amino acid-functionalized benzene-1,3,5-tricarboxamide (BTA) derivatives affect their self-assembly behavior in nonpolar solvents. Specifically, we examine the roles of linker flexibility, steric hindrance introduced by bulky substituents at the 2,4,6-positions, and the nature of the central core (aromatic vs aliphatic). Our results show that these structural changes lead to strikingly different aggregation outcomes, ranging from monomeric species and ill-defined oligomers to well-defined nanocapsules. These findings highlight the importance of precise molecular design in controlling supramolecular self-assembly and demonstrate how specific structural factors dictate the morphology and properties of the resulting materials.

## Graphical abstract

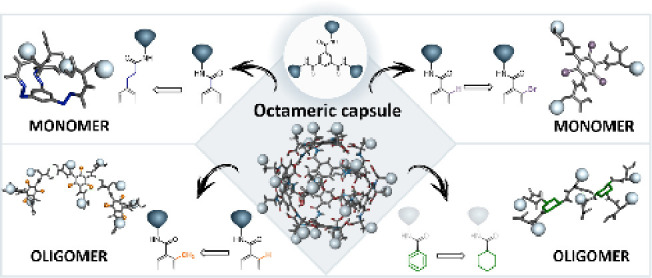

## Introduction

Biological structures often arise from intricate molecular self-assembly processes, including the construction of cell membranes, DNA base pair recognition, β-sheet formation, polypeptide chain folding, and enzyme catalysis.^1,2^ In these systems, hydrogen bonding plays a pivotal role in determining the structure and function of biological molecules, as it is one of the most effective types of supramolecular interactions for shaping both inter- and intramolecular orientations.^3,4^ These orientations can be readily modified through structural alterations of the monomers involved.^5,6^ The pattern and nature of the hydrogen bonding network dictate the self-assembly mechanism, ultimately influencing the morphology and function of resulting supramolecular aggregates.^4,7–10^

Nature provides numerous elegant examples of hydrogen bonding interactions, offering a rich source of inspiration.^11^ Despite significant progress in recent decades, fully harnessing these interactions to create functional organic materials remains a challenging task, with many questions still unanswered.^12–15^ Achieving the desired morphology of supramolecular assembly requires careful consideration of molecular design, particularly the selection of specific structural features or substituents that guide self-assembly toward the desired architecture.^16^ Such considerations provide valuable insights into how changes in molecular structure impact self-assembly behavior, which is crucial for the design and creation of functional organic materials. Among the molecules well-suited for constructing various functional supramolecular aggregates are derivatives of benzene-1,3,5-tricarboxamide (BTA).^7,16–21^

Their facile synthesis, versatility for modification, and ability to incorporate various substituents on a side chains make a wide range of derivatives accessible.^22^ These structural variations are not merely a cosmetic but play a crucial role in how these molecules interact with one another.^23^ The aggregation of BTA derivatives into larger structures is heavily influenced by their molecular structure. Specific structural features can either promote or hinder aggregation, leading to a variety of outcomes. The aggregation behavior of BTA derivatives can be finely tuned by modifying both the periphery and the central core of the monomer.^24^ These factors have been studied to understand the structural variety and self-assembly mechanisms of various supramolecular assemblies.^25–28^ For example, grafting amino acids onto the periphery promotes the formation of hydrogen bonds, significantly enhancing the strength of the intermolecular interactions.^29–32^ Modifications to the central core provide insights into how structural changes can influence self-assembly behavior.^22,33,34^ Importantly, increasing steric hindrance around the BTA core can induce a greater dihedral angle between the amide group and the aromatic ring, thereby promoting stronger intermolecular hydrogen bonding and favoring aggregation.^35,36^ At the same time, an expanded aromatic surface in BTA derivatives enhances π–π stacking and solvophobic interactions, which may, counterintuitively, limit the length of the resulting aggregates by stabilizing monomeric species in certain solvents.^36^ These competing effects underscore the need for a systematic dissection of structure–assembly relationships, particularly for the rationale of supramolecular systems with targeted functions.

The self-assembly of suitably modified BTA molecules has yielded dimeric, columnar, and capsular structures with potential applications as nucleating agents for polymeric materials,^37^ noncovalent cross-linkers in thermoplastic elastomers,^18^ metallogels,^38,39^ organogelators,^40^ porous organic materials,^41^ and for the reversible and selective storage of fullerenes.^17^ Furthermore, the simple molecular design and broad availability of BTA derivatives, combined with a well-established understanding of their self-assembly behavior, have paved the way for their application across diverse fields, including nanotechnology and biomedical sciences. Reported applications include their use as MRI contrast agents,^42^ metal ion coordination,^43^ drug delivery via microcapsules,^44^ and nanoreactors for efficient catalysis.^45,46^

Among the many BTA monomers examined for these purposes, derivatives modified with *S*-trityl-L-cysteine (*S*-Tr-Cys) have garnered considerable attention due to their ability to form octameric nanocapsules.^17^ While this research has provided valuable insights into self-assembly and guest-binding events, a detailed molecular-level understanding remains limited. Further exploration is needed to understand how monomer structural features influence self-assembly behavior through both intra- and intermolecular interactions and how they modulate the morphology of the resulting supramolecular architectures.

In this work, we focus on the benzene-1,3,5-tricarboxamide (BTA) derivative with *S*-Tr-Cys arms (**3a**), where amino-acid moieties act as H-bond donors and acceptors, yielding a well-defined octameric capsule held together by 48 hydrogen bonds in both the solid state and solution.^17^ Herein, we evaluate the effects of introducing a flexible linker (**3b**), the steric effects of substituents at the 2, 4, and 6 positions (bromo (**3c**) and methyl (**3d**) moieties), and the switch to an aliphatic core (**3e**) on the self-assembly outcome in chlorinated solvents (Figure 1). Our research demonstrates that the structural modifications introduced into the originally studied monomer (**3a**) significantly influence the nature of the resulting supramolecular aggregates. Depending on the molecular structure and the functional groups present, these molecules may assemble into morphologically distinct oligomeric products (**3d**, **3e**) or remain at the monomeric level, forming intramolecularly bonded structures (**3b**, **3c**). Furthermore, these structural variations within the molecular components not only lead to significant differences in aggregation behavior but also result in distinct morphologies of the assemblies.

**Figure 1:**
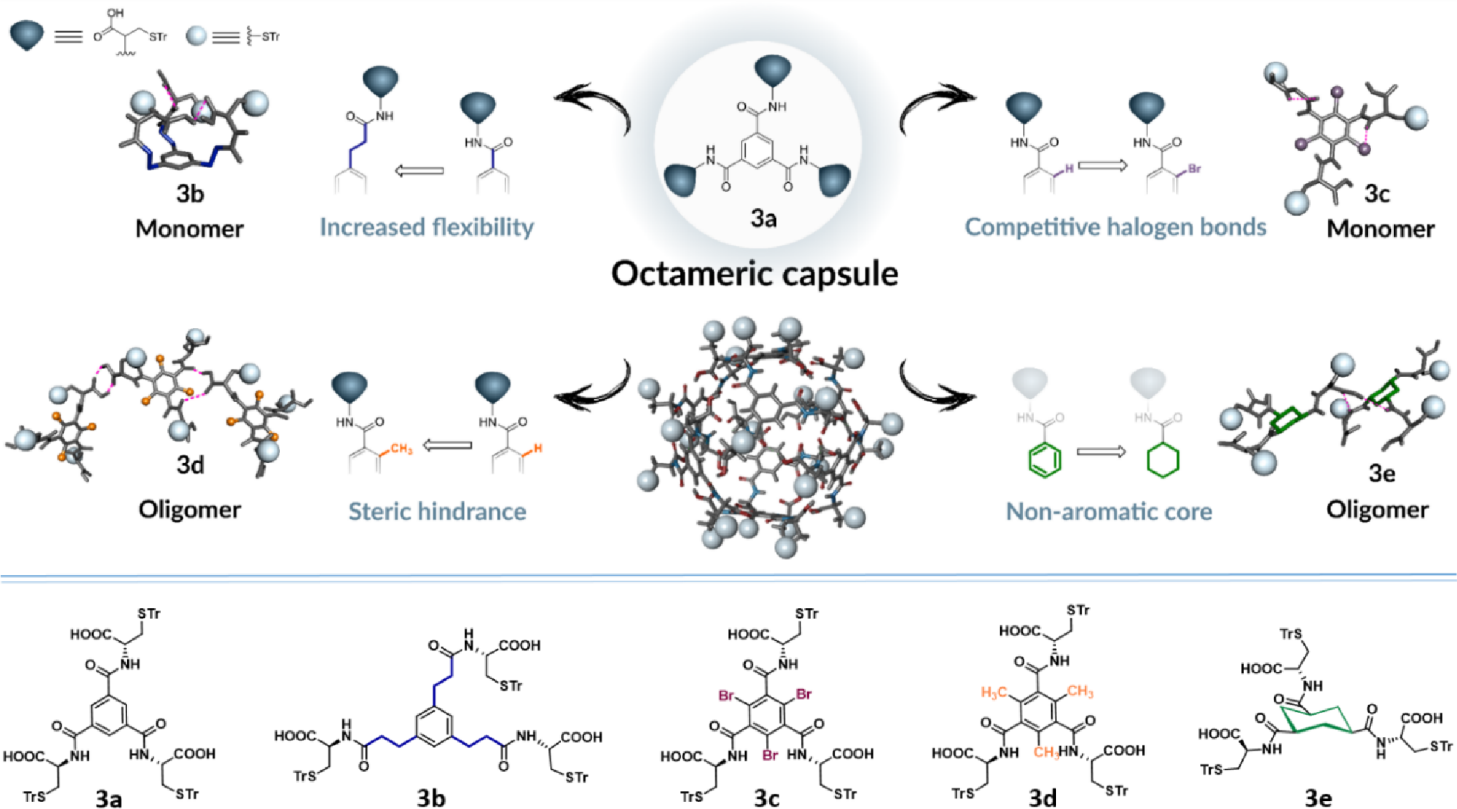
Schematic representation of the self-assembly of **3a**–**3e** in chlorinated solvents.

## Results and Discussion

### Synthesis

The synthesis of components **3a**–**3e** began from the appropriate tricarboxylic acids, either commercially available (**1a**, **1e**) or obtained according to literature procedures (**1b**–**1d**).^20,47–49^ Detailed descriptions of the synthetic protocols and complete characterization data for all new compounds are available in the Supporting Information. The synthesis of **3a** and **3e** followed a known literature method involving the reaction of *S*-Tr-protected L-cysteine with the NHS-activated esters of benzene-1,3,5-tricarboxylic and *cis*,*cis-*cyclohexane-1,3,5-tricarboxylic acids, respectively.^17^ Compounds **3b**–**3d** were synthesized by the condensation of *S*-Tr-protected L-cysteine with acyl trichorides generated from the corresponding triacids. The detailed synthesis is described in the SI. All components were purified by recrystallization from DCM/*n*-hexane mixtures, yielding the final materials as white solids, confirmed by ^1^H and ^13^C NMR, FT-IR, ESI-MS, and LC-MS analyses (see the SI for details).

### Self-Assembly of **3b**: Role of a Flexible Linker

The first modification of the original building block **3a** ^17^ involved extending the functional arms by two carbon atoms to distance the amino-acid moieties away from the aromatic core (Figure 1). We hypothesized that the increased flexibility might significantly affect the hydrogen bonding pattern observed in the octameric capsule, potentially changing the assembly outcome.

Initial evidence of different behaviors in solution was observed through the comparison of the ^1^H NMR spectra of **3a** and **3b** in chlorinated solvents conducive to hydrogen bonded assemblies, such as CDCl_3_ (Figure 2). As previously reported,^17^ the formation of an octameric nanocapsule from **3a** is indicated by the splitting of ^1^H NMR resonances into three sets of signals, each corresponding to a different arm of the tripodal building block. This splitting occurs due to the loss of the monomer’s *C*_3_ symmetry upon incorporation into the octamer, rendering the three arms inequivalent. In contrast, the ^1^H NMR spectrum of **3b** recorded in CDCl_3_ retained the 3-fold symmetry observed in DMSO-*d*_6_ solution (Figure 2c), with sharp and well separated resonances. To determine whether these supramolecular structural differences were concentration-dependent, we conducted experiments in chloroform across a concentration range from 1 × 10^–2^ to 1 × 10^–5^ M (Figure S49 in the Supporting Information). No discernible changes were detected in the ^1^H NMR spectrum, with the signal remaining well-separated and sharp, suggesting the presence of a monomeric form in the chlorinated solvent.

**Figure 2:**
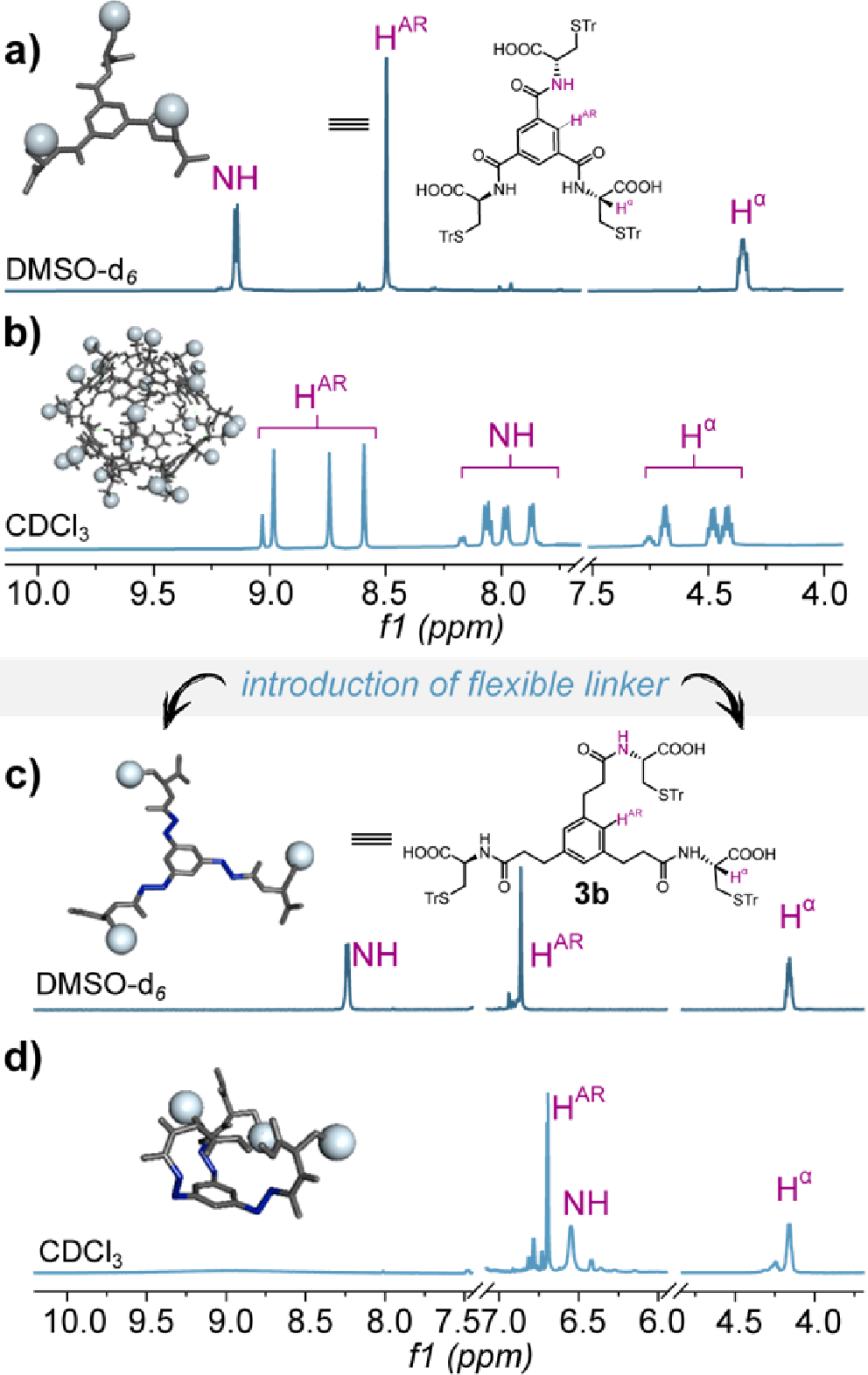
^1^H NMR (600 MHz, *T* = 298 K, *C* = 1.0 × 10^–2^ M) spectra of (a) **3a** in DMSO-*d*_6_, (b) **3a** in CDCl_3_, (c) **3b** in DMSO-*d*_6_, and (d) **3b** in CDCl_3_.

To further validate these findings, we employed diffusion-ordered NMR spectroscopy (DOSY NMR) to assess the size of the supramolecular species of **3b** in CDCl_3_ compared to the solvated monomer observed in the hydrogen-bond-disrupting solvent, i.e., DMSO-*d*_6_ (Table 1, Figures S35 and S36 in the Supporting Information). The experiments revealed that the solvodynamic radii of **3b** in both solvents were nearly identical, being 9.2 Å for DMSO-*d*_6_ (observed diffusion coefficient 1.07 × 10^–10^ m^2^ s^–1^) and 9.3 Å for CDCl_3_ solution (observed diffusion coefficient 4.50 × 10^–10^ m^2^ s^–1^). These results suggest that **3b**, due to its elongated and flexible arms, engages primarily in intramolecular rather than intermolecular H-bonding, leading to the formation of monomeric species instead of noncovalent assemblies in chlorinated solvents. To further investigate the structure of **3b**, we applied FT-IR spectroscopy to both **3a** and **3b** in solution (CHCl_3_ and THF; Figures S43 and S44 in the Supporting Information) and in the solid state.

**Table 1:** ^1^H DOSY NMR (600 MHz, *T* = 298 K, *C* = 1.0 × 10^–2^ M) Data for **3a**–**3e** in Different Solvents

solvent	compound	*D* ^c^ × 10^–10^ [m^2^ s^–1^]	*r*_sol_ ^d^ [Å]	***V***_**sph**_ ^e^ **[Å** ^3^ **]**
DMSO-*d*_6_	**3a**	1.09	9.1	3155
	**3b**	1.07	9.3	3380
	**3c**	1.13	8.8	2870
	**3d**	1.10	9.1	3155
	**3e**	1.11	8.9	3020
CDCl_3_ or TCE-*d*_2_	**3a**	2.90^a^	14.3	12,240
	**3b**	4.50^a^	9.2	3260
	**3c**	7.50^a^	5.5	700
	**3d**	2.35^a^	17.7	23,220
	**3e**	0.80^b^	18.7	13,570

a CDCl_3_.

b TCE-*d*_2_.

c Diffusion coefficient.

d Solvodynamic radius.

e Spherical volume.

The most indicative FT-IR bands to study the structures of **3a** and **3b** are the N–H and C=O stretches, as these are significantly influenced by hydrogen bond formation and do not overlap with the solvent IR cutoffs (Figure 3). The FT-IR spectra of **3a** recorded in CHCl_3_ and in KBr pellets displayed a fully symmetrical pattern, with a single νN–H band at 3370 cm^–1^ and the corresponding νC=O at 1720, along with amide I and II bands at 1650 and 1600 cm^–1^, respectively, characteristic of H-bonded amido and carboxylic groups.^16,50^ This pattern aligns with the structure of the octameric nanocapsule, whose self-assembly is driven by cooperative N–H···O=C and O–H···O=C hydrogen bonds. In contrast, the FT-IR spectrum of **3b** in CHCl_3_ showed a different stretching pattern, with three νN–H bands at 3420 and 3325 cm^–1^, νC=O bands at 1740 and 1720 cm^–1^, and amide I and II bands at 1670, 1635, 1605, and 1595 cm^–1^. The stretches correspond well to the mixture of free (unbound) and H-bonded (NH···O=C, OH···O=C) centers,^16,50^ as illustrated in Figures 4b and S44 in the Supporting Information. Notably, esterification of the carboxylic centers with ethyl units (**3b-OEt**) prohibited the formation of direct intramolecular hydrogen bonding interactions (Figure S45 in the Supporting Information).

**Figure 3:**
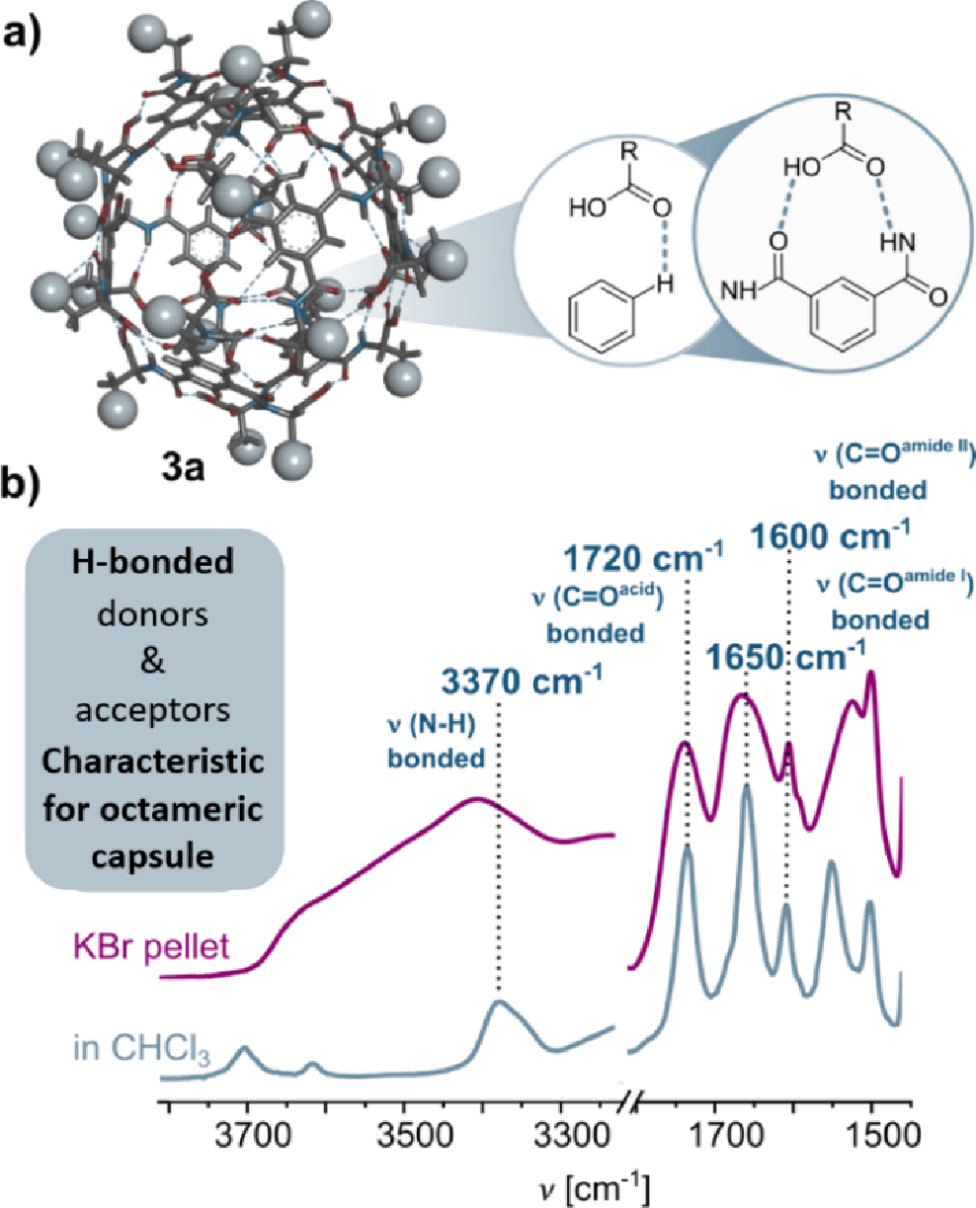
(a) Crystal structure of the octameric capsule (**3a**) with marked hydrogen bonds. Some of the H atoms have been omitted for the sake of clarity. (b) FT-IR characterization of the octameric capsule (**3a**) in CHCl_3_ solution (*C* = 1.0 × 10^–2^ M and *T* = 298 K) and in the KBr pellet with assigned bands. Zoom in on the N–H and C=O regions.

**Figure 4:**
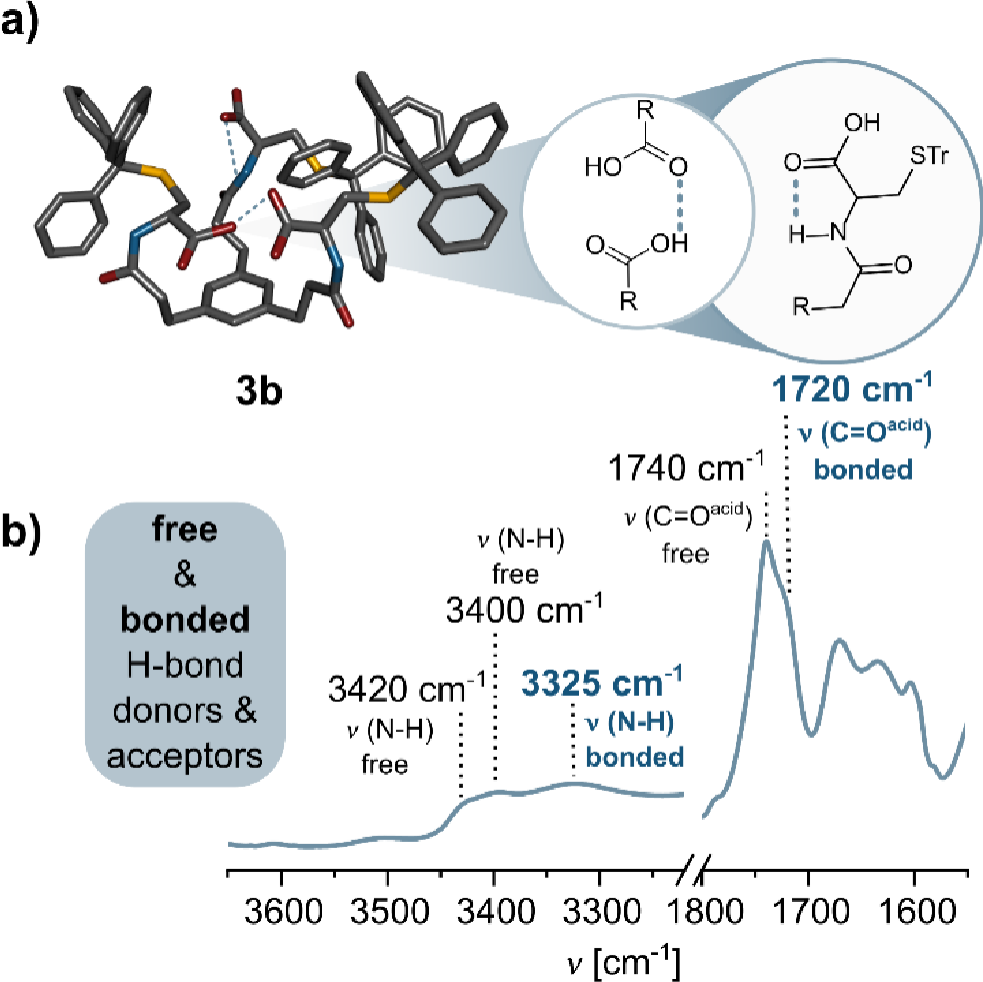
(a) MM2 model of **3b** with marked hydrogen bonds. Some of the H atoms have been omitted for clarity. (b) FT-IR characterization of **3b** in CHCl_3_ solution (*C* = 1.0 × 10^–2^ M and *T* = 298 K) with assigned bands. Zoom on the N–H and C=O regions.

The combined results from NMR and FT-IR analyses confirm that **3b** does not assemble into octameric capsules nor columnar polymer-structures commonly observed for *C*_3_ symmetric BTA derivatives.^30^ ,^40^ ,^51^ Instead, **3b** forms an intramolecularly H-bonded monomeric podand-type species, as depicted in the MM2 model in Figure 4.

### Self-Assembly of **3c** and **3d**: The Effect of the Competitive Hydrogen/Halogen Bonding and Bulky Substituents on the 2,4,6-Positions

In component **3a**, the 2,4,6-positions on the central benzene core are unsubstituted. After self-assembly, these positions occupy significant space within the nanocapsule, directly facing the hydrogen bonding array. A careful examination of the X-ray crystal structure of the octameric nanocapsule (Figure 3a) reveals that the 2,4,6-hydrogen atoms in **3a** play two key roles: (a) they form supplementary C–H···O hydrogen bonds, which positively influence the overall thermodynamic stability, and (b) due to minimal hindrance and the absence of any significant repulsive forces, they do not disturb the self-assembly process. These roles may contribute to the remarkable stability of the capsular assembly.^17^ To explore the influence of 2,4,6-substituents on the self-assembly outcome, two new building blocks were designed and synthesized: (a) **3c**, in which the hydrogen atoms were replaced with bromine (−Br) substituents, and (b) **3d**, where methyl (−CH_3_) groups were introduced. The bromine atoms were expected to influence both the electronic environment of other ring substituents and potentially participate in halogen bonding, while methyl groups were anticipated to introduce steric effects.

For **3c**, its ^1^H NMR spectrum recorded in CDCl_3_ displayed very broad peaks, with only the trityl resonances clearly resolved (Figure 5a). The signals sharpened upon heating or the addition of H-bond acceptor solvents, such as DMSO-*d*_6_ or acetone-*d*_6_ (Figure 5b and Figure S53 in the Supporting Information). The broad and unresolved ^1^H NMR spectrum observed for **3c** was found to be concentration-independent (Figure S51 in the Supporting Information), suggesting an intramolecular rather than intermolecular self-assembly process.^7^ DOSY NMR measurements in CDCl_3_ confirmed this, revealing a single species with an estimated solvodynamic radius of only 5.5 Å (diffusion coefficient 7.5 × 10^–10^ m^2^ s^–1^, Table 1, Figures S37 and S38 in the Supporting Information). This solvodynamic size is significantly smaller than that observed for the solvated monomers of **3a**–**3c** (≈9 Å, Table 1), indicating that **3c**, due to intramolecular bonding, undergoes structural wrapping instead of aggregation in noncompetitive solvents.

**Figure 5:**
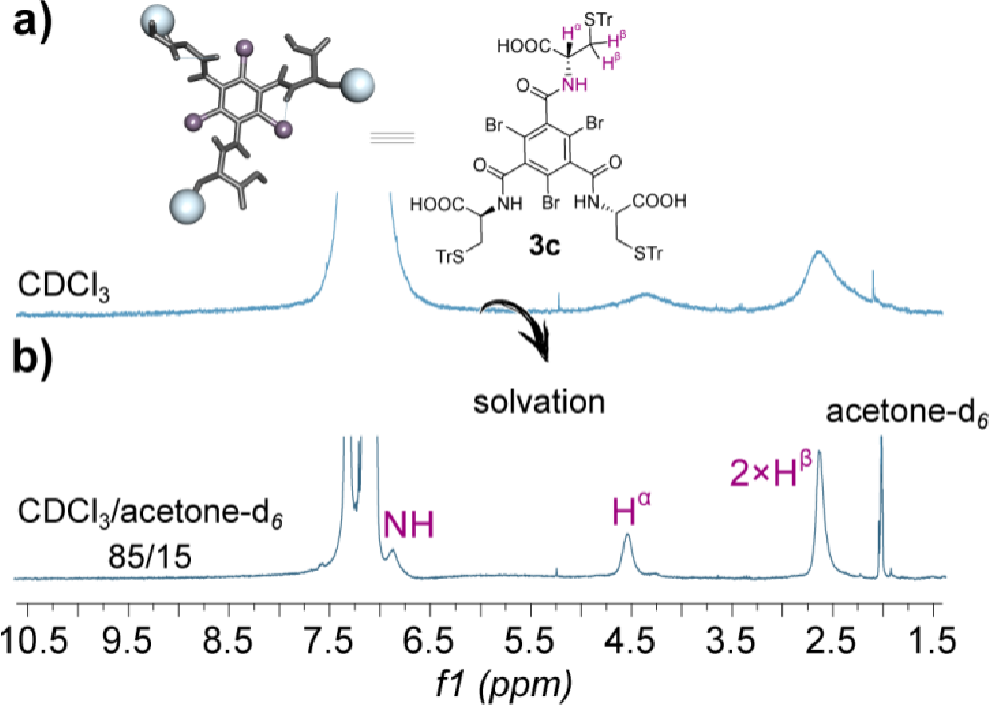
^1^H NMR (600 MHz, *T* = 298 K, *C* = 1.0 × 10^–2^ M) spectra of **3c** in (a) CDCl_3_ and (b) CDCl_3_/acetone-*d*_6_ mixture (85:15 v/v).

FT-IR spectroscopy, combined with MM2 modeling (Figure S54 in the Supporting Information), provided further insights into **3c**’s behavior in noncompetitive media. The bulky −Br substituents at the 2,4,6-positions induce off-plane twisting of the electron-rich 1,3,5-amide units (Figure S54a in the Supporting Information), displacing the hydrogen bonding centers and inhibiting the self-assembly process. The FT-IR spectra of **3c** in CDCl_3_, KBr pellets, and THF showed that upon dissolution in noncompetitive media, **3c** loses the symmetric interaction pattern observed for **3a** (Figure 3). Instead, several bathochromically shifted bands appear, with νN–H stretches at 3400, 3355, 3330, and 3270 cm^–1^, νC=O bands at 1754 and 1725 cm^–1^, and amide I and II bands at 1675, 1655, and 1595 cm^–1^ (Figures S46 and S55 in the Supporting Information).^50^ These findings suggest that the monomeric form of **3c** possesses a disordered array of intramolecular hydrogen (OH···O=C, NH···O=C, NH···Br)^50,52^ and halogen bonds (C=O···Br) between N–H, C=O, COOH, and −Br subunits, as shown in Figure S54 in the Supporting Information. This array is easily disrupted in competitive media, e.g., THF, where strong solvent–solute hydrogen bonds stabilize the molecule (Figure S46 in the Supporting Information). The FT-IR results align with the NMR study, where strong solvation effects in DMSO-*d*_6_ and acetone-*d*_6_ were also observed (Figure 5b).

To further investigate the morphology of the aggregates, additional analyses were performed using scanning electron microscopy (SEM) and atomic force microscopy (AFM) (Figures S57a and S58a in the Supporting Information). The samples were prepared by spin-coating solutions of **3c** (*C* = 2.5 × 10^–^^4^ M in TCE), which formed thin, amorphous films upon solvent evaporation. However, the results did not reveal interpretable or consistent nanostructural features that would significantly complement the solution-phase data.

For **3d**, the results above demonstrate that the 2,4,6-positions significantly influence the interactions involved in aggregation. While the CH_3_ groups in **3d** are more spatially demanding than bromine atoms, they were not expected to introduce repulsive forces in a highly polar environment with competitive supramolecular interactions. The ^1^H NMR spectrum of **3d** in CDCl_3_ is similar to that of **3c**, with broad and unresolved peaks (Figure 6b). These features could result from either the self-assembly of **3d** or the intramolecular wrapping observed for **3b** and **3c**. To resolve this ambiguity, DOSY NMR was employed, revealing that the signal broadening in **3d** is indeed due to self-assembly in noncompetitive solvents, rather than structural collapse. The solvodynamic radius of **3d** was found to be 17.7 Å (observed diffusion coefficient 2.35 × 10^–10^ m^2^ s^–1^, Figure S40 in the Supporting Information), nearly double the size of its solvated monomer in DMSO-*d*_6_ (Table 1, 9.1 Å, Figure S39 in the Supporting Information). To further confirm the self-assembly of **3d**, variable temperature circular dichroism (VT-CD) was employed to provide detailed information about the mechanism of supramolecular self-assembly. Changes in the CD effect at λ = 224 nm were monitored while cooling the solutions of **3d** at three different concentrations (*C* = 1.0–5.0 × 10^–4^ M) in DCE (1,2-dichloroethane) from 350 to 250 K (Figure 6c). VT-CD data showed a sigmoidal response to temperature, a transition characteristic of either dimerization or an equal-K isodesmic polymerization process. Although both processes are indistinguishable in this analysis,^7,53^ the overall symmetry of the ^1^H NMR spectrum and the apparent DOSY size (exceeding not only the dimer^54^ but even the octameric assembly **3a**) clearly point toward an isodesmic polymerization, with Δ*H*_m_ = −16.2 kJ mol^–1^ and *T*_m_ = 275 K at 5.0 × 10^–4^ M (Figure 6d). The negative enthalpy change confirms that the assembly is enthalpy-driven, but the relatively low association energy translates into the low thermal stability of the resulting aggregate. Notably, **3d** shows also a minor CD effect at the molecularly dissolved state as a result of a direct connection of the chiral centers to the chromophore. This effect is pronounced not only at elevated temperatures (Figure 6c) but also in spectra recorded in a highly polar solvent (THF, Figure S60 in the Supporting Information) and has been observed previously for amino acid-derived supramolecular synthons.^8,10,13^

**Figure 6:**
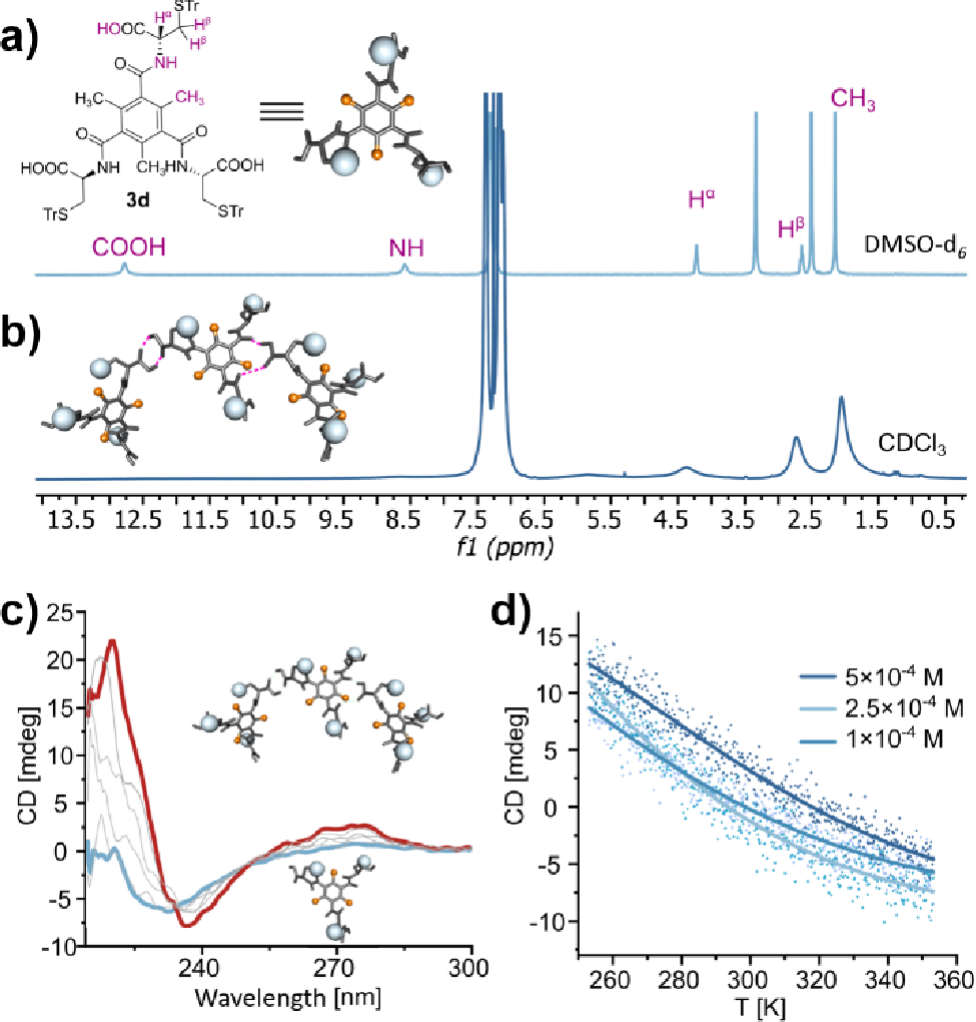
(a) ^1^H NMR (600 MHz *T* = 298 K, *C* = 1.0 × 10^–2^ M) spectrum of **3d** in DMSO-*d*_6_. (b) ^1^H NMR (600 MHz *T* = 298 K, *C* = 1.0 × 10^–2^ M) spectrum of **3d** in CDCl_3_. (c) VT-CD spectra recorded during cooling of a solution of **3d** in DCE (*C* = 5.0 × 10^–4^ M, *T* = 350–250 K, cooling rate −1 K min^–1^). Spectra for the highest and lowest temperatures are colored red and blue, respectively. (d) Changes in the CD intensity (λ = 225 nm) recorded during cooling of the solutions of **3d** in DCE at various concentrations (*T* = 350–250 K, cooling rate −1 K min^–1^).

To identify the noncovalent forces responsible for the polymerization, FT-IR spectroscopy was conducted. The spectra provided evidence of intramolecular COOH···HOOC and CONH···HOOC hydrogen bonds,^16,50^ along with some unoccupied hydrogen bonding centers, with νN–H bands at 3410, 3375, and 3275 cm^–1^, νC=O bands at 1750 and 1725 cm^–1^, and amide I and II bands at 1660 (broad) 1620, 1595, and 1580 cm^–1^ (Figure S47 in the Supporting Information). Notably, one of those interactions, between amide C=O and amide −NH groups from two tripod arms, bridged by two hydrogen bonds with COOH units, is similar to the interaction considered the main driving force for the self-assembly of the octameric nanocapsule **3a**. It appears that while the steric demand of CH_3_ groups of the benzene ring is not high enough to prevent the formation of hydrogen bonded bridges between the tripod arms, it does disturb the overall geometry of the **3d** molecules. This disturbance prevents the formation of a closed and highly confined capsular nanostructure, as seen in **3a**. Instead, **3d** molecules in noncompetitive media exhibit marginal thermodynamic stability, forming a poorly ordered oligomeric assembly similar to the MM2 model shown in Figure S55a in the Supporting Information.

In line with the analysis of **3c**, SEM and AFM imaging were also performed for compound **3d** using a similar sample preparation protocol (Figures S57b and S58b in the Supporting Information). Unfortunately, **3d** also formed an amorphous film upon solvent evaporation, with no evidence of gelation or higher-order aggregate formation, rendering these data of limited utility for the structural characterization of **3d**.

### Self-Assembly of **3e**: Role of an Aliphatic Core

1,3,5-Cyclohexyltrisamide-based scaffolds represent a class of *C*_3_-symmetric amino acid ligands well-known for forming one-dimensional columnar structures, organogelators, and thickeners,^33,55,56^ although most studies have focused on their behavior in the solid state or aqueous solutions.^29^ Given the success with building blocks **3a**–**3d**, we extended our investigation to the study of the aggregation of **3e** in organic solvents suitable for hydrogen bonding formation. Due to the poor solubility of **3e** in CHCl_3_, TCE was selected as the solvent, as it provides higher solubility while maintaining low polarity and a noncompetitive environment favorable to studying hydrogen bonded assemblies. However, the aliphatic nature of **3e**, lacking a UV chromophore, precluded analysis using the VT-CD technique in TCE due to a solvent cutoff.^33^ The ^1^H NMR spectrum of **3e** in TCE-*d*_2_ displayed very broad signals, preventing the exact assignment of signals. Nonetheless, DOSY NMR analysis revealed a solvodynamic radius of 18.7 Å (observed diffusion coefficient 0.80 × 10^–10^ m^2^ s^–1^, Figure S42 in the Supporting Information), which is consistent with the formation of an oligomeric assembly product, similar to the one observed for **3d**. FT-IR spectra in TCE, THF, and KBr pellets (Figure S48 in the Supporting Information) indicated that both amide and carboxylic acid units of **3e** participate in hydrogen bond formation in noncompetitive media,^50^ with observed vibrations of νN–H 3335 cm^–1^, νC=O at 1725 cm^–1^, and amide I and II bands at 1630 and 1595 cm^–1^ (Figure 7). Considering the possible conformations of the cyclohexane ring with three bulky *S*-Tr-Cys arms in the 1,3,5-positions, we anticipate that **3e** forms an oligomer in which bulky substituents occupy equatorial positions in a chair conformation, held together by intramolecular hydrogen bonds, as shown in Figure 7. As with compounds **3c** and **3d**, SEM and AFM imaging of the **3e** oligomer (Figures S57c and S58c in the Supporting Information) did not provide interpretable or consistent nanostructural features that could meaningfully support the proposed assembly mode in solution.

**Figure 7:**
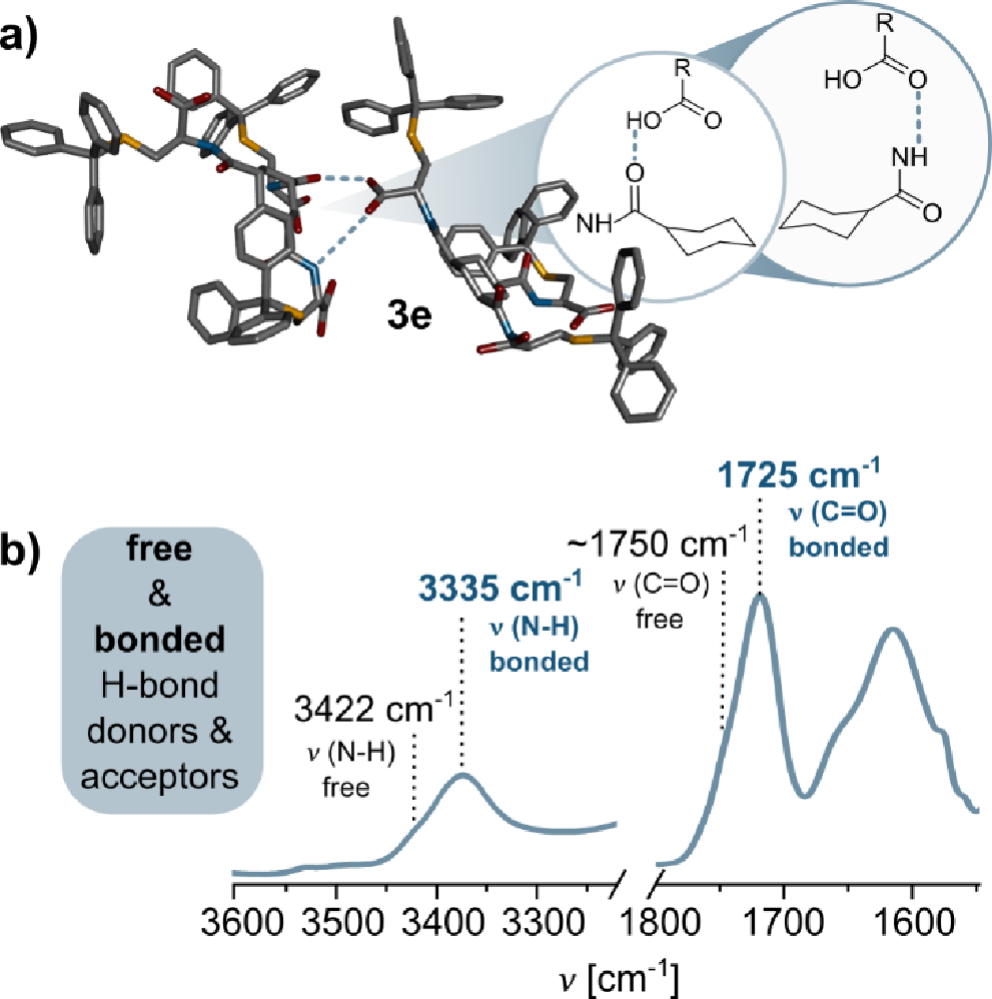
(a) MM2 model of **3e** with marked hydrogen bonds. Some of the H atoms were omitted for clarity. (b) FT-IR characterization of **3e** in CHCl_3_ solution (*C* = 1.0 × 10^–2^ M and *T* = 298 K) with assigned bands. Zoom on the N–H and C=O regions.

## Conclusions

This study highlights the transformative impact of structural modifications on the self-assembly behavior of benzene-1,3,5-tricarboxamide (BTA) derivatives. By systematically exploring derivatives **3a**–**3e**, we reveal how linker flexibility, steric effects, and core structure govern supramolecular aggregation and morphology. For example, a flexible linker in **3b** disrupts capsule formation, favoring monomeric structures through intramolecular hydrogen bonding. Similarly, steric hindrance and competitive supramolecular interactions in **3c** prevent formation of larger assembly, while methyl substitution in **3d** achieves an optimal interplay of forces, enabling oligomer formation. An aliphatic core in **3e** further demonstrates the essential role of the core rigidity in forming nanocapsules.

This work is among the first to systematically demonstrate how structural changes in amino acid-functionalized BTA derivatives dictate a wide range of supramolecular assemblies. The study provides new insights into the role of competitive intra- and intermolecular interactions, particularly in systems like **3b** and **3c**, where hydrogen bonding is modulated by steric and electronic effects. An important takeaway from this study is that when designing building blocks for the self-assembly of supramolecular capsules or polymers, careful attention must be paid to the flexibility of the arms bearing the binding groups and the steric effects within the molecules. Both insufficient and excessive conformational flexibility at the molecular level can completely disrupt the supramolecular self-assembly process, particularly when competitive intramolecular interactions are possible. This research underscores the importance of precise molecular design in supramolecular chemistry, offering a comprehensive exploration of how structural modifications at the molecular level translate to macroscopic assembly behaviors. The insights gained here may facilitate the rational design of supramolecular carriers, nanocapsules, or soft materials for applications in drug delivery, diagnostics, and molecular recognition.

## Supplementary Material



## Data Availability

The data underlying this study are available in the published article and its Supporting Information.

## Notes

Experimental procedures, characterization data for all compounds, and NMR, IR, CD, UV–vis, LC-MS, and ESI-MS spectra of the products, and ORD and SEM experiments (PDF)
