# Supplementary material for: From Monomers to Nanocapsules: The Role of Structural Features in Amino-Acid-Derived BTA Self-Assembly
Source: J Org Chem. 2025 Oct 8;90(41):14557–64. doi: 10.1021/acs.joc.5c01500 (PMC12538580; doi:10.1021/acs.joc.5c01500)
Supplement: Supplementary file 1 [file jo5c01500_si_001.pdf]

Supporting Information for

From Monomers to Nanocapsules: The Role of Structural Features in Amino-Acid-Derived BTA Self-Assembly

Anna Walczak, Grzegorz Markiewicz, Michał Gliński, Miroslava Čonková, and Artur R. Stefankiewicz*

Table of contents

1. Materials and Methods S2
2. Synthetic procedures and molecular characterization S3
   2.1. Synthesis of 3b S3
   2.2. Synthesis of 3b-OEt S9
   2.3. Synthesis of 3c S13
   2.4. Synthesis of 3d S19
   2.5. Synthesis of 3e S25

2.6. DOSY NMR S29

2.7. FT-IR S33

3. Study self-assembly S36
4. Literature: S44

1. Materials and Methods

The following abbreviations were used in this file: DMSO: dimethyl sulfoxide; THF: tetrahydrofuran; DCE; 1, 2-Dichloroethane. Chemicals were purchased from commercial suppliers, mainly Merck KGaA (Germany) and Fluorochem Ltd (UK) and used as received.

NMR spectra were recorded on Bruker Fourier 300 MHz, or Bruker Avance IIIHD 600 MHz spectrometers, and referenced on solvent residual peaks. Sample temperature was controlled with internal probe heaters and Bruker Co. (USA) BCU II variable-temperature unit. NMR deuterated solvents were purchased from Deutero GmbH (Germany), at min. >99.9% isotopic enrichment (or higher) and used as received. The NMR data were processed using Mestrelab Research MNova software. DOSY NMR spectra were recorded using 2D LEDbp-pulse sequence (ledbpgp2s). Δ and δ were optimised with 1D sequence (ledbpgp2s1d) to achieve sufficient signal attenuation at 95% gradient strength. All DOSY experiments were recorded at T = 298 K.

ESI-MS spectra were recorded on Bruker Impact HD Q-TOF or Bruker qTOF Compact spectrometers operating in positive ion mode. Samples were dissolved in MS-grade acetonitrile, and the spectra were recorded accordingly. Theoretical MS spectra were predicted using the Mestrelab Research MNova software.

IR spectra were obtained with Jasco Co. (Japan) 4000 FTIR spectrophotometer, operating with 4 cm^{-1} resolution, with 128 scans/spectrum, in the airtight CaF_2 cuvette (Specac, UK) of 0.2 mm pathlength. Spectra of the pure solvents were used for subtraction.

CD spectra were recorded on Jasco Co. (Japan) *J*-1500 CD spectropolarimeter, operating at 1 nm bandwidth and 400 nm/min scanning speed. Sample temperature was controlled with the Peltier-type variable-temperature unit (Jasco Co., PTC-510). Measurements were performed in DCE solutions using quartz cuvettes (Hellma GMbH, Germany) with 0.5 - 1 mm optical path. Spectra of the pure solvents were used as the baselines.

ORD spectra were recorded using a Jasco J-1500 CD spectropolarimeter (Jasco Co., Japan) equipped with an ORDM-520 optical rotatory dispersion unit, operating over a wide angular range (90°).

SEM (Scanning Electron Microscopy) analyses were performed using an FEI Quanta 250 FEG microscope. All samples were prepared using the slow evaporation method on silicon wafers. Each Samples were prepared as $2,5 \times 10^{-4}$ M solutions in TCE, filtered through a 0.22 µm syringe filter, and deposited onto the surface of a silicon wafer pre-cleaned with acetone. The deposition process was repeated the following day, after the initial solvent droplets had evaporated. Samples were left to dry in a fume hood for three days and further dried under vacuum one day prior to measurements. SEM imaging was conducted under the mildest conditions that still ensured sufficient resolution: a chamber pressure of 70 Pa and an accelerating voltage of 5 kV.

AFM (Atomic Force Microscopy) analyses were carried out using an Agilent Technologies 5500 atomic force microscope (USA) equipped with All-In-One-Al probes (Budget Sensors), cantilever type C (nominal force constant: 7.4 N/m; resonance frequency: 150 kHz). All measurements were performed in tapping mode under ambient air conditions, with a typical scan speed of 0.3 lines/s. Samples were prepared as $2,5 \times 10^{-4}$ m solutions in TCE and deposited onto a thin layer of a silicon wafer via spin coating.

2. Synthetic procedures and molecular characterization

2.1. Synthesis of 3b

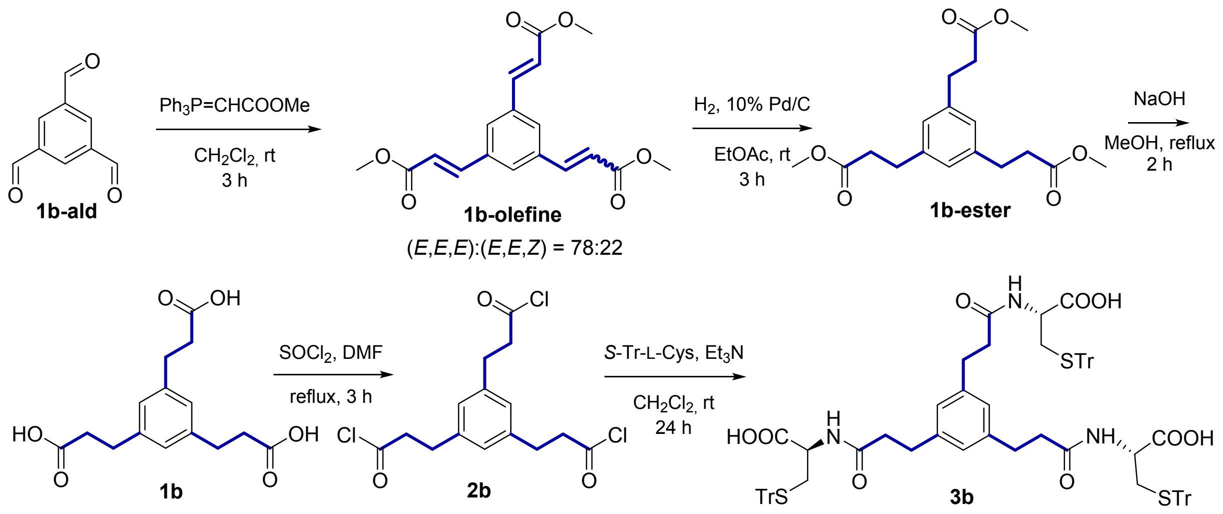

1b-olefine: To the solution of benzene-1,3,5-tricarbaldehyde (324 mg, 2 mmol) in dichloromethane (70 mL) $Ph_3P=CHCOOMe$ (2.97 g, 9 mmol) was added. Reaction mixture was stirred for 3 hours at ambient temperature. Solvent was removed under reduced pressure and the residue was dissolved in ethyl acetate (100 mL). Solution containing $ZnCl_2$ (272 mg) and ethyl acetate (5 mL) was added to precipitate Zn complex of $Ph_3P=O$[1]. After stirring overnight, precipitate was filtered and solvent was removed under reduced pressure. Product was purified by column chromatography using n-hexane:ethyl acetate (3:1) as eluent. Product was obtained in 66% yield (435 mg) as a white amorphous solid, in agreement with literature data.[2] ***(E,E,E)*-1b-olefine**: ^{1}H NMR (300 MHz, $CDCl_3$) δ 7.69 (d, J = 16.1 Hz, 1H), 7.65 (s, 1H), 6.50 (d, J = 16.0 Hz, 1H), 3.83 (s, 3H).

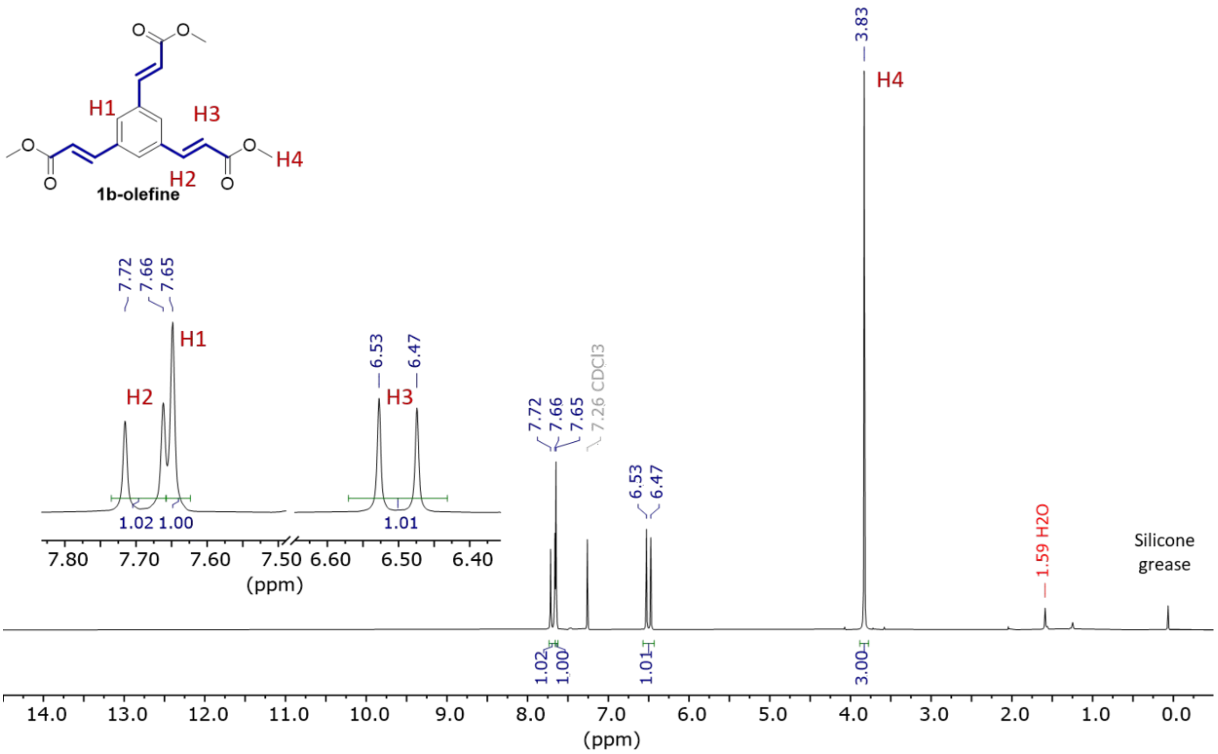

Fig. S1. ^{1}H NMR (300 MHz, $CDCl_3$) of ***(E,E,E)*-1b-olefine**.

1b-ester: The compound is known and was prepared according to a previously published procedure[2a]. To the solution of **1b-olefine** (330 mg, 1 mmol) in ethyl acetate (30 mL) 10% Pd/C (220 mg) were added. Reaction was stirred for 3 hours at ambient temperature and pressure in the H_2 atmosphere. After, Pd/C was filtered through silica gel plug and solvent was removed under reduced pressure. Product was obtained as light yellow oil in excellent yield 95% (320 mg). **1b-ester**: ^{1}H NMR (300 MHz, $CDCl_3$) δ 6.87 (s, 1H), 3.67 (s, 3H), 2.88 (t, *J* = 7.8 Hz, 2H), 2.59 (t, *J* = 8.6, 7.1 Hz, 2H).

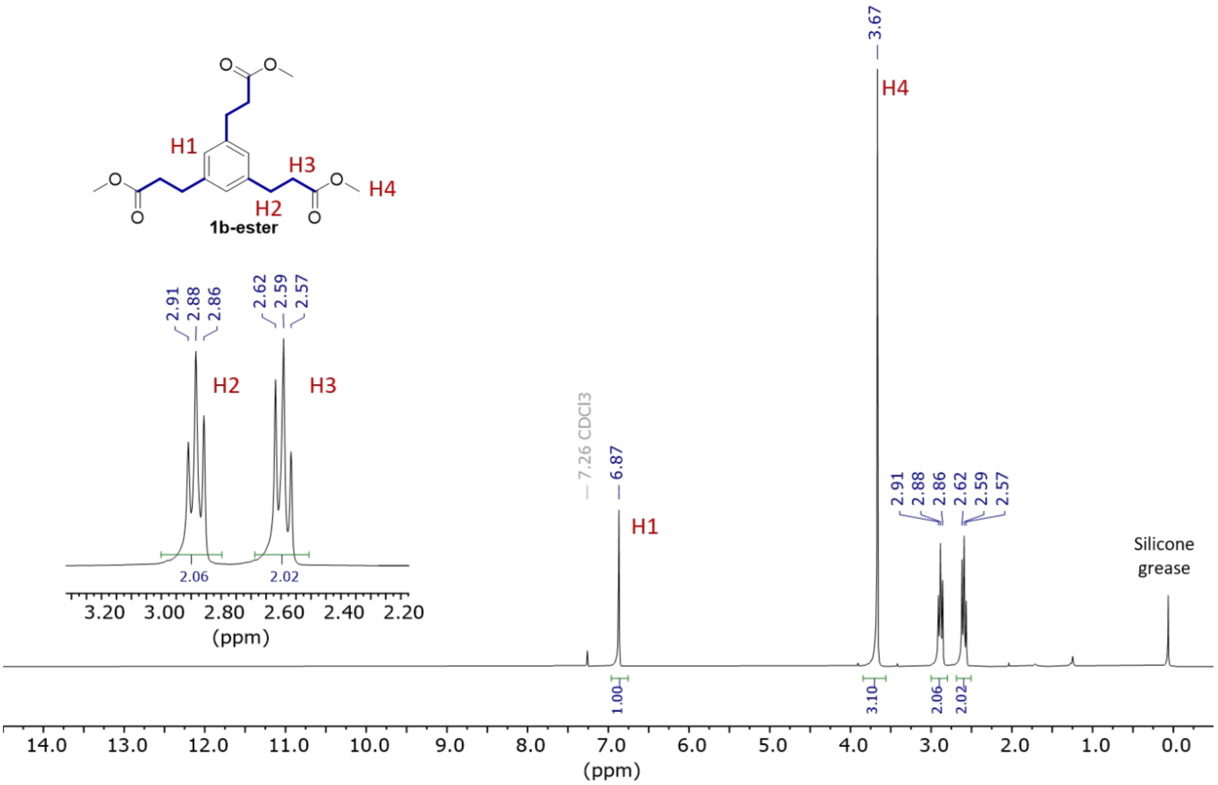

Fig. S2. ^{1}H NMR (300 MHz, $CDCl_3$) of **1b-ester**.

1b: The compound is known and was prepared according to a previously published procedure.[2a] To the solution of **1b-ester** (302 mg, 0.9 mmol) in methanol (45 mL) 4 M NaOH (30 mL) was added. Reaction mixture was heated to reflux for 2 hours in an oil bath at 70 °C. Methanol was removed under the reduced pressure, then water (60 mL) was added and pH was adjusted to pH = 1 with 1M HCl. Resulting solution was extracted with ethyl acetate (3 x 40 mL), combined organic layers were dried over Na_2SO_4 and filtered. Solvent was removed under reduced pressure and the residue was recrystallized from the dichloromethane/*n*-hexane mixture. Product was collected as a white solid in good 83% yield (220 mg). **1b**: ^{1}H NMR (300 MHz, MeCN-d_3) δ 8.93 (s, 1H), 6.93 (s, 1H), 2.81 (t, J = 7.7 Hz, 2H), 2.55 (t, J = 7.9 Hz, 2H), HRMS (TOF-MS) m/z: $[M+Na]^+$ calcd. for $C_{15}H_{18}O_6Na$ 317.0996, Found 317.1004.

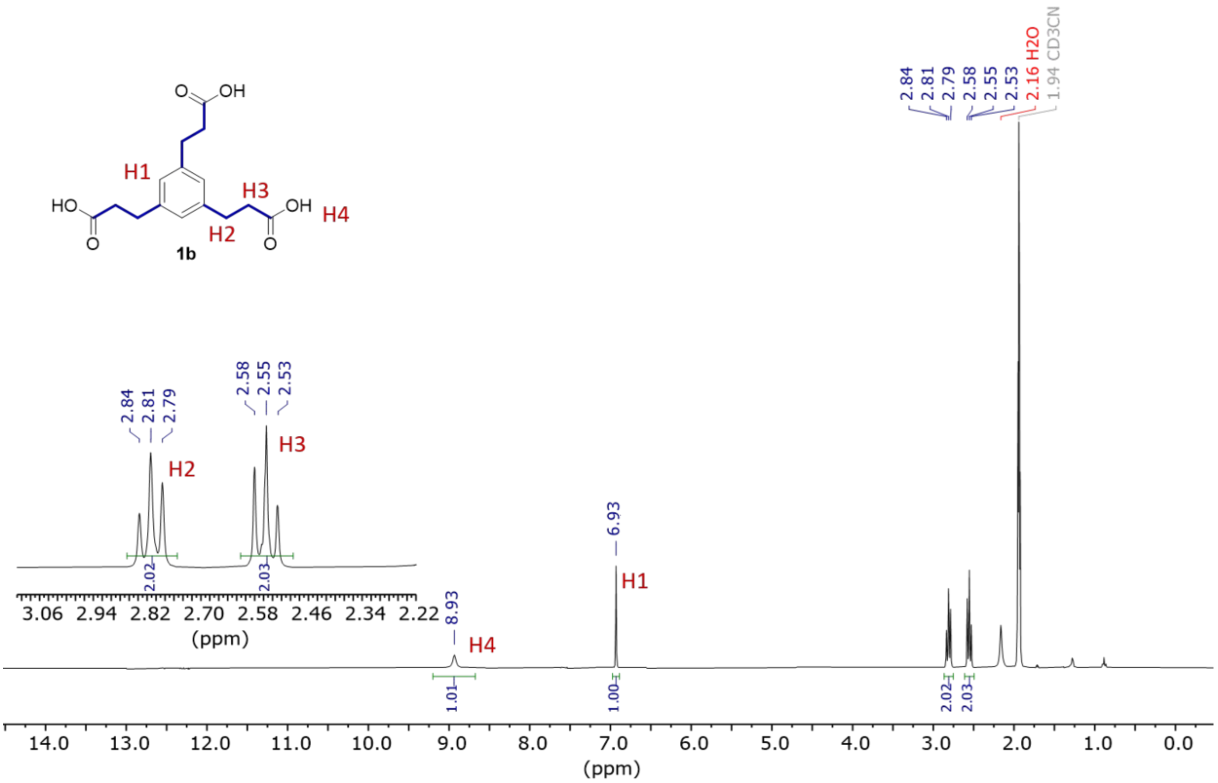

Fig. S3. ^{1}H NMR (300 MHz, MeCN-d_3) of **1b**.

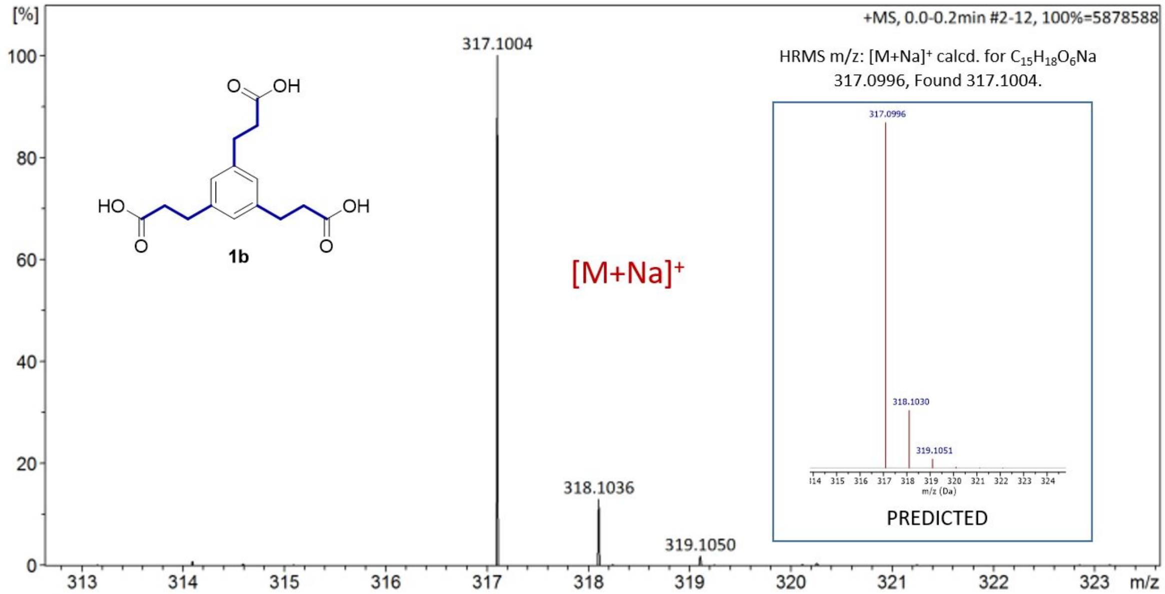

Fig. S4. ESI-MS of **1b**.

2b: To the suspension of **1b** (100 mg, 0.34 mmol) in $SOCl_2$ (15 mL) catalytic amount of DMF (*N*,*N*-dimethylformamide , 1 drop) was added. Reaction mixture was refluxed for 3 hours in an oil bath at 80 °C. After, $SOCl_2$ was removed under the reduced pressure and the crude product was used in the next reaction.

3b: To the suspension of *S*-Trityl-l-cysteine (727 mg, 2 mmol) in dichloromethane (15 mL) Et_3N (Triethylamine, 0.27 ml, 2 mmol) was added, followed by **2b** prepared by previous reaction. Resulting solution was stirred at ambient temperature in Ar atmosphere for 24 hours. Solvent was removed under reduced pressure and resulting oil was dissolved in acetone (10 mL), which was then added dropwise to vigorously stirring 1M HCl (100 mL).

White precipitate was collected by filtration, washed with water (3 x 15 mL) and dried in vacuum. Product was isolated in 80% yield (362 mg) as a white amorphous solid.

^{1}H NMR (600 MHz, DMSO-d_6) δ 12.76 (s, 1H), 8.24 (d, *J* = 8.1 Hz, 1H), 7.37 – 7.19 (m, 15H), 6.86 (s, 1H), 4.16 (q, *J* = 8.2 Hz, 1H), 2.70 (t, *J* = 7.8 Hz, 2H), 2.55 (d, *J* = 6.1 Hz, 2H), 2.41 – 2.33 (m, 2H).

^{13}C{^{1}H} NMR (151 MHz, DMSO-d_6) δ 171.8, 171.5, 144.2, 141.1, 129.1, 128.1, 126.8, 125.7, 66.1, 51.3, 36.7, 33.2, 31.0, 25.4.

HRMS (TOF-MS) m/z: [M+Na]$^+$ calcd. for $C_{81}H_{75}N_3O_9S_3Na$ 1352.4558, Found 1352.4523.

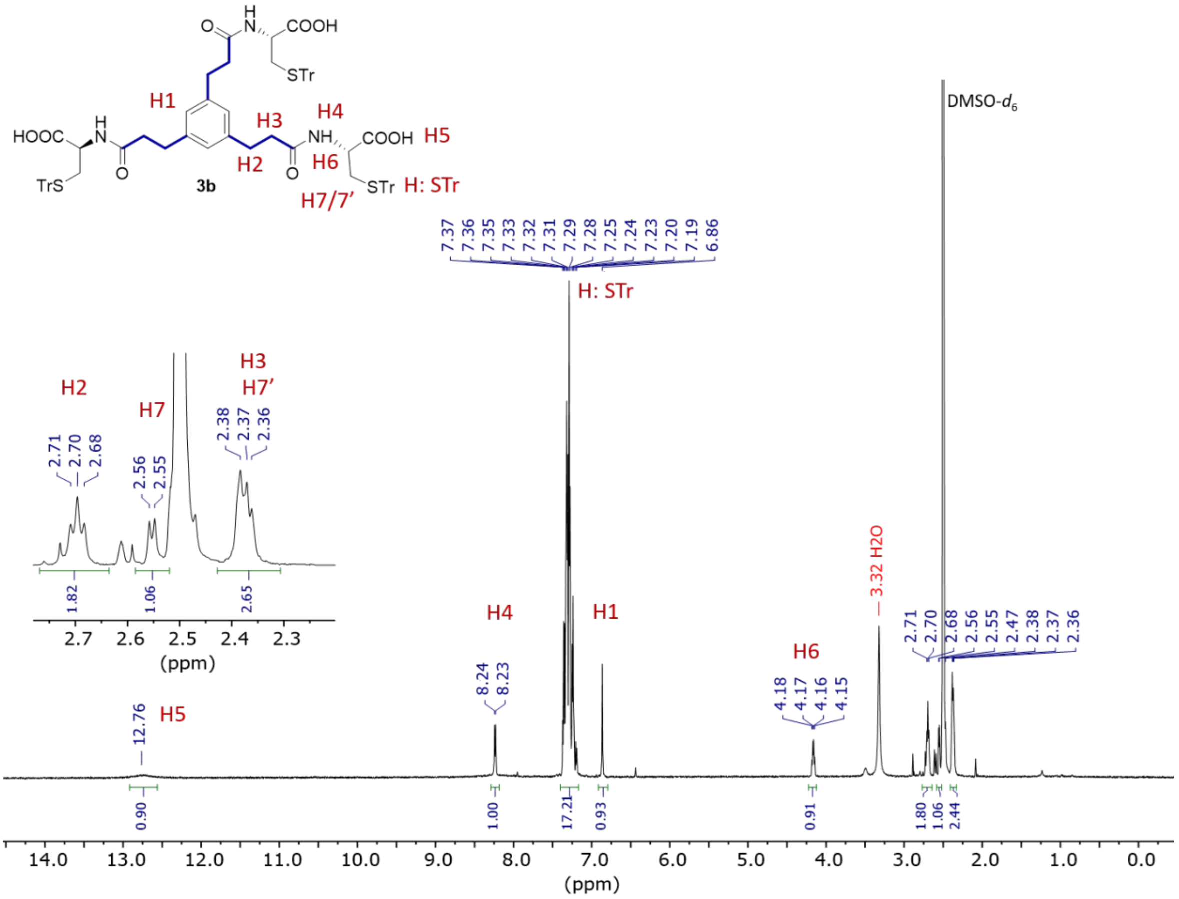

Fig. S5. ^{1}H NMR (600 MHz, DMSO-d_6) of **3b**.

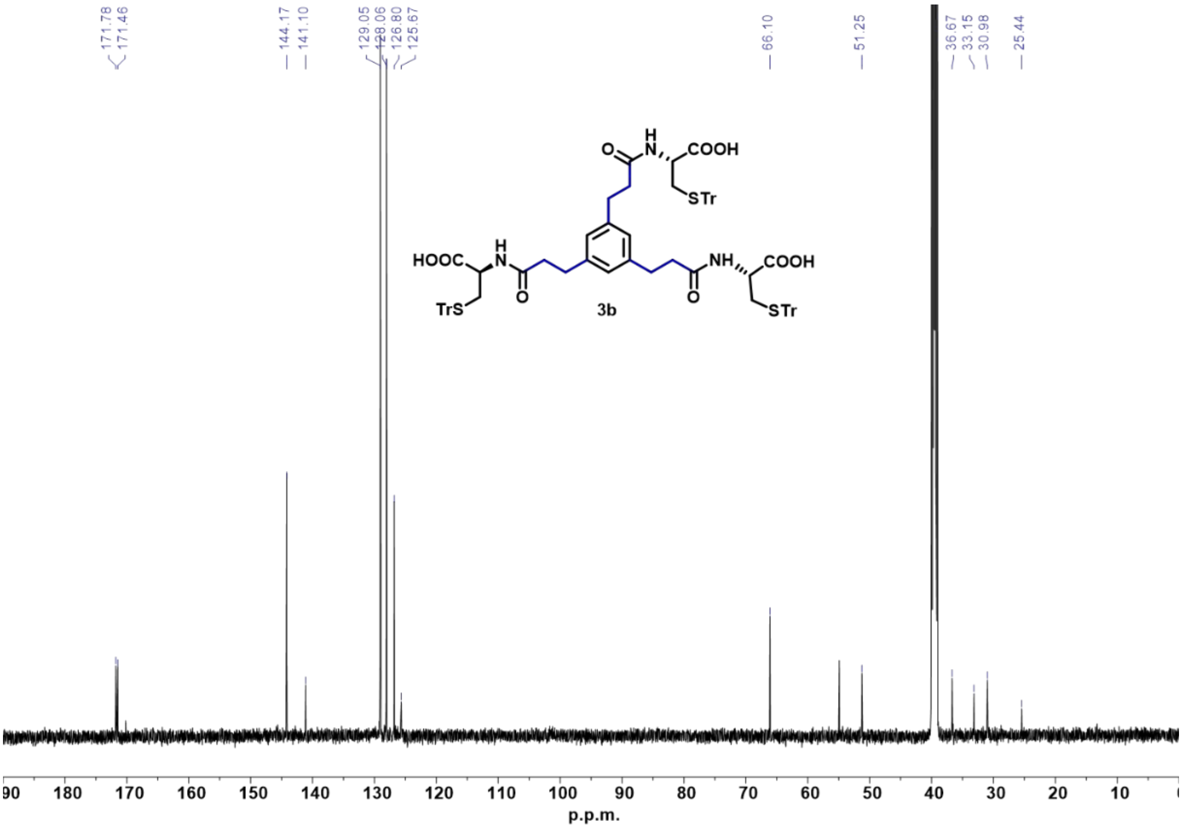

Fig. S6. $^{13}C\{^{1}H\}$ NMR (151 MHz, DMSO-d_6) of **3b**.

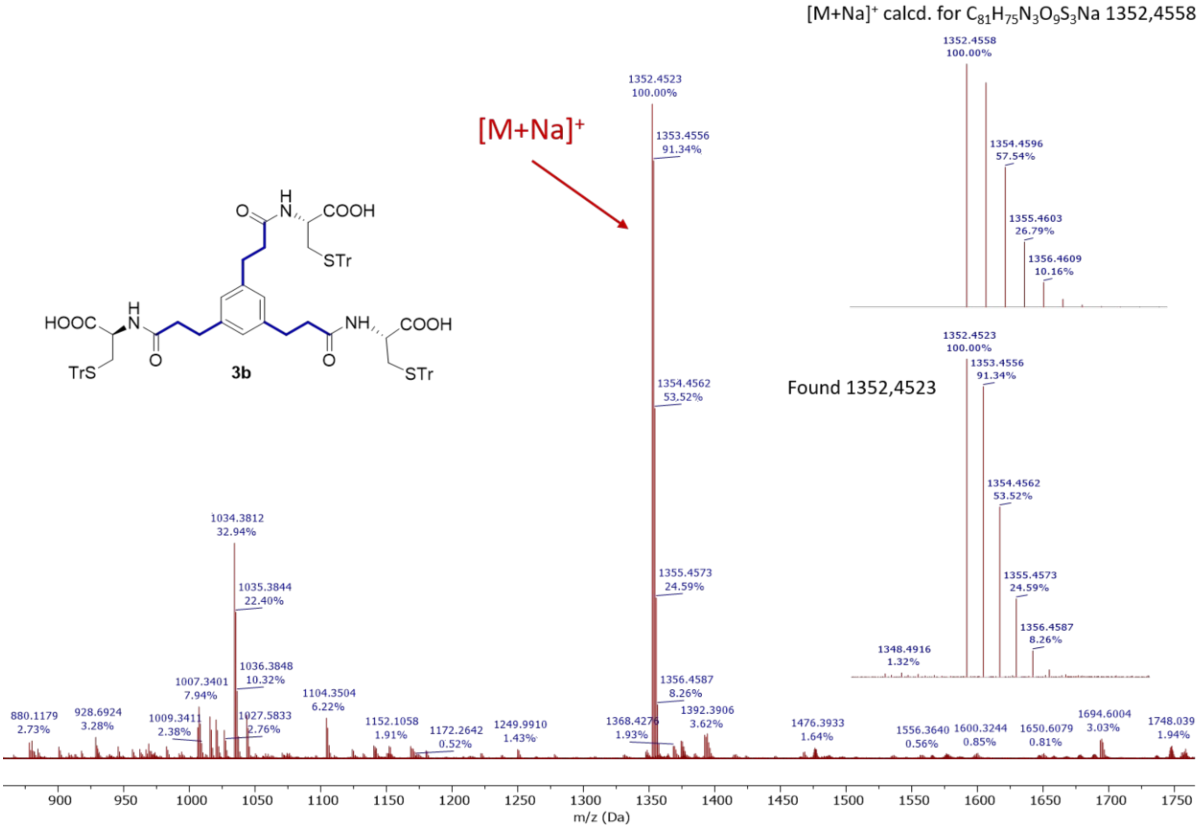

Fig. S7. ESI-MS of **3b**.

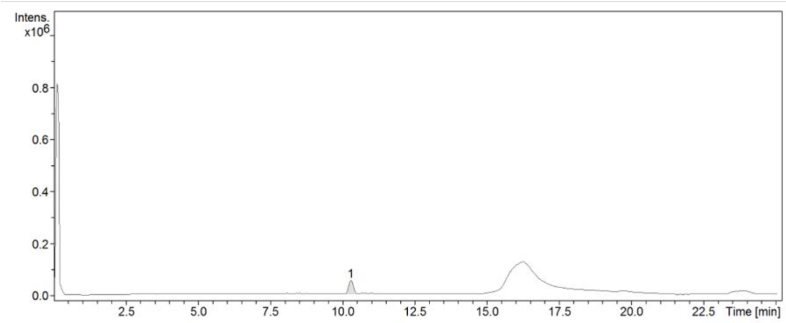

#	RT [min]	Area	Area %	Area Frac. %	I	Int. %	Chromatogram	Max. m/z
1	10.3	550284	100.00	100.00	60202	100.00	BPC +All MS	1330.4702

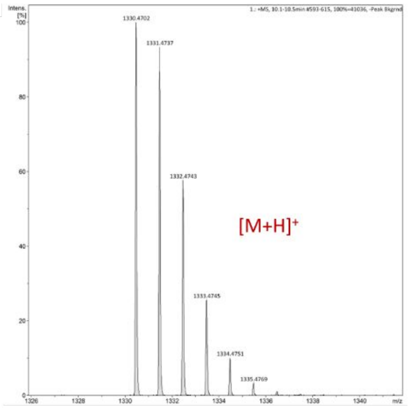

Fig. S8. LC-MS of **3b**.

2.2. Synthesis of 3b-OEt

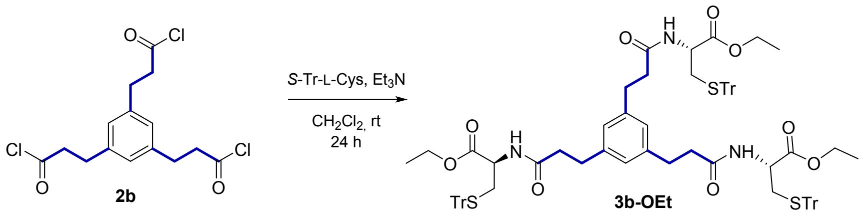

***S*-Tr-L-Cys-OEt:** The compound is known and was prepared according to a previously published procedure[3]. L-Cysteine ethyl ester hydrochloride (1.54 g, 8,7 mmol, 1 eq.) and triphenylmethanol (3.31 g, 12,7 mmol, 1.45 eq.) were dissolved in TFA (16 mL). The resulting orange solution was stirred at room temperature for 2 hours. Then, 10 mL of H_2O and 4 M NaOH solution were added to neutralize the mixture to pH 5.5. The solution was extracted with chloroform, and the organic layer was dried and evaporated to dryness under reduced pressure. The resulting residue was dissolved in diethyl ether, precipitated with n-hexane, and evaporated to dryness under reduced pressure. A white amorphous solid was obtained (2.81 g, 82%).

^{1}H NMR (300 MHz, DMSO-d_6) δ 8.49 (s, 2H), 7.47 – 7.05 (m, 15H), 4.17 (q, *J* = 7.1 Hz, 2H), 3.85 (t, *J* = 5.8 Hz, 1H), 2.57 (d, *J* = 5.9 Hz, 2H), 1.19 (t, *J* = 7.1 Hz, 3H).

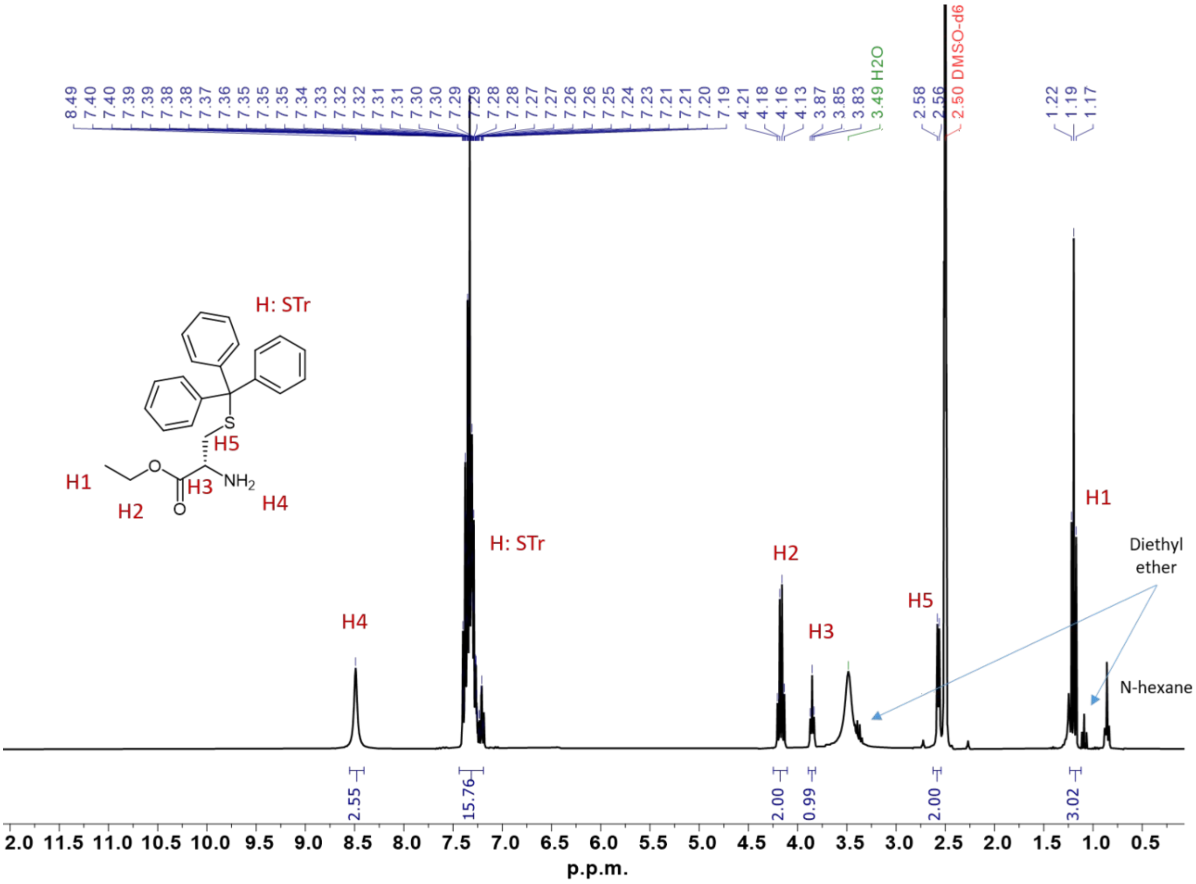

Fig. S9. ^{1}H NMR (400 MHz, DMSO-d_6) of ***S*-Tr-L -Cys-OEt**.

3b-OEt: To the suspension of *S*-Trityl-L-cysteine-OEt (727 mg, 2 mmol) in dichloromethane (15 mL) Et_3N (Triethylamine, 0.27 ml, 2 mmol) was added, followed by **2b**. Resulting solution was stirred at ambient temperature in Ar atmosphere for 24 hours. Solvent was removed under reduced pressure and resulting oil was dissolved in acetone (10 mL), which was then added dropwise to vigorously stirring 1M HCl (100 mL). White precipitate was collected by filtration, washed with water (3 x 15 mL) and dried in vacuum. The product was isolated by column chromatography (eluent: n-hexane/ethyl acetate, 1:1) as a white amorphous solid in 45% yield (203 mg).

^{1}H NMR (400 MHz, DMSO-d_6) δ 8.34 (d, *J* = 7.8 Hz, 1H), 7.45 – 7.13 (m, 15H), 6.86 (s, 1H), 4.09 (ddd, *J* = 8.1, 5.3 Hz, 1H), 4.05 – 3.91 (m, 2H), 2.73 – 2.66 (m, 2H), 2.57 – 2.55 (m, 1H), 2.42 – 2.32 (m, 3H), 1.08 (t, *J* = 7.1 Hz, 3H).

^{13}C{^{1}H} NMR (101 MHz, DMSO-d_6) δ 172.0, 170.7, 144.6, 141.5, 129.2, 128.6, 127.3, 126.2, 66.8, 61.2, 52.04, 39.9, 37.0, 33.3, 31.4, 14.4.

HRMS (TOF-MS) m/z: [M+Na]$^+$ calcd. for $C_{87}H_{86}D^*_1N_3O_9S_3Na$ 1436.5497, Found 1436.5550. *Note: This analysis was performed on NMR sample (DMSO-*d6*).

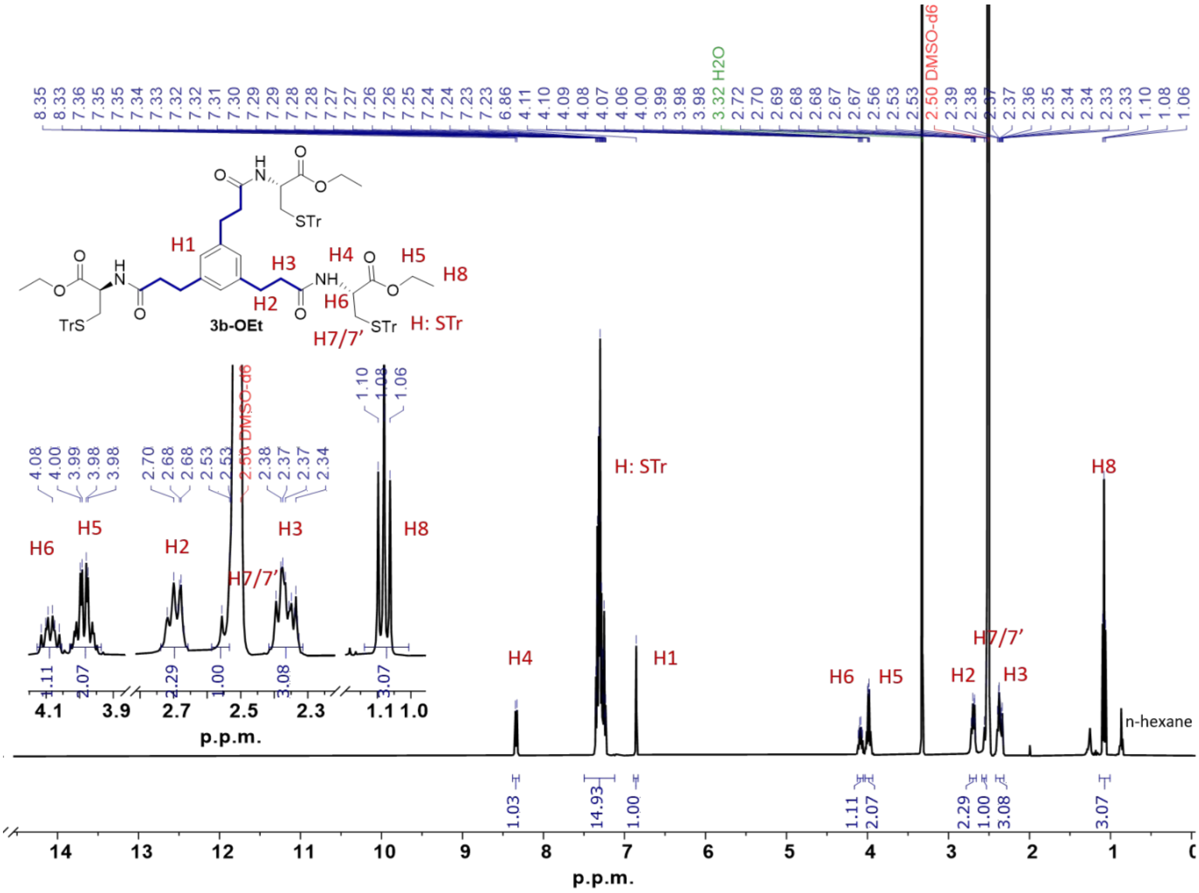

Fig. S10. ^{1}H NMR (400 MHz, DMSO-d_6) of **3b-OEt**.

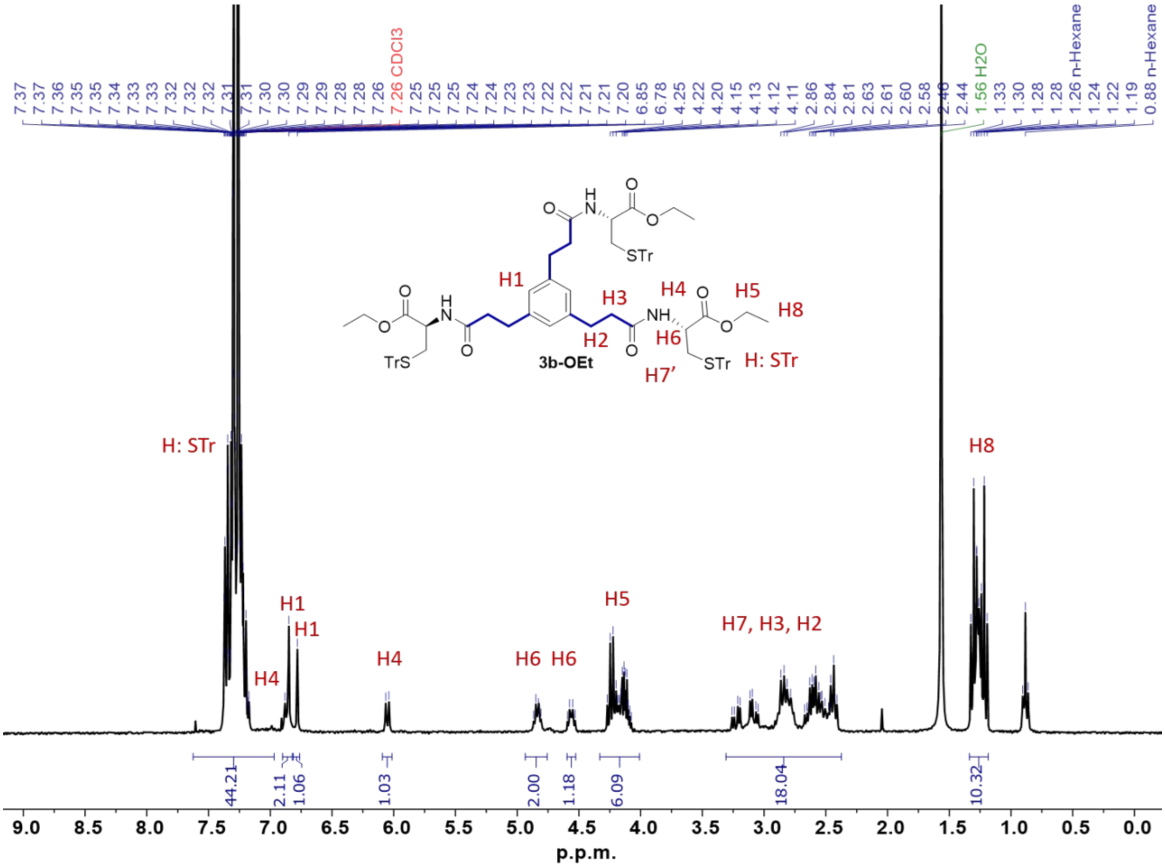

Fig. S11. ^{1}H NMR (400 MHz, $CDCl_3$) of **3b-OEt**.

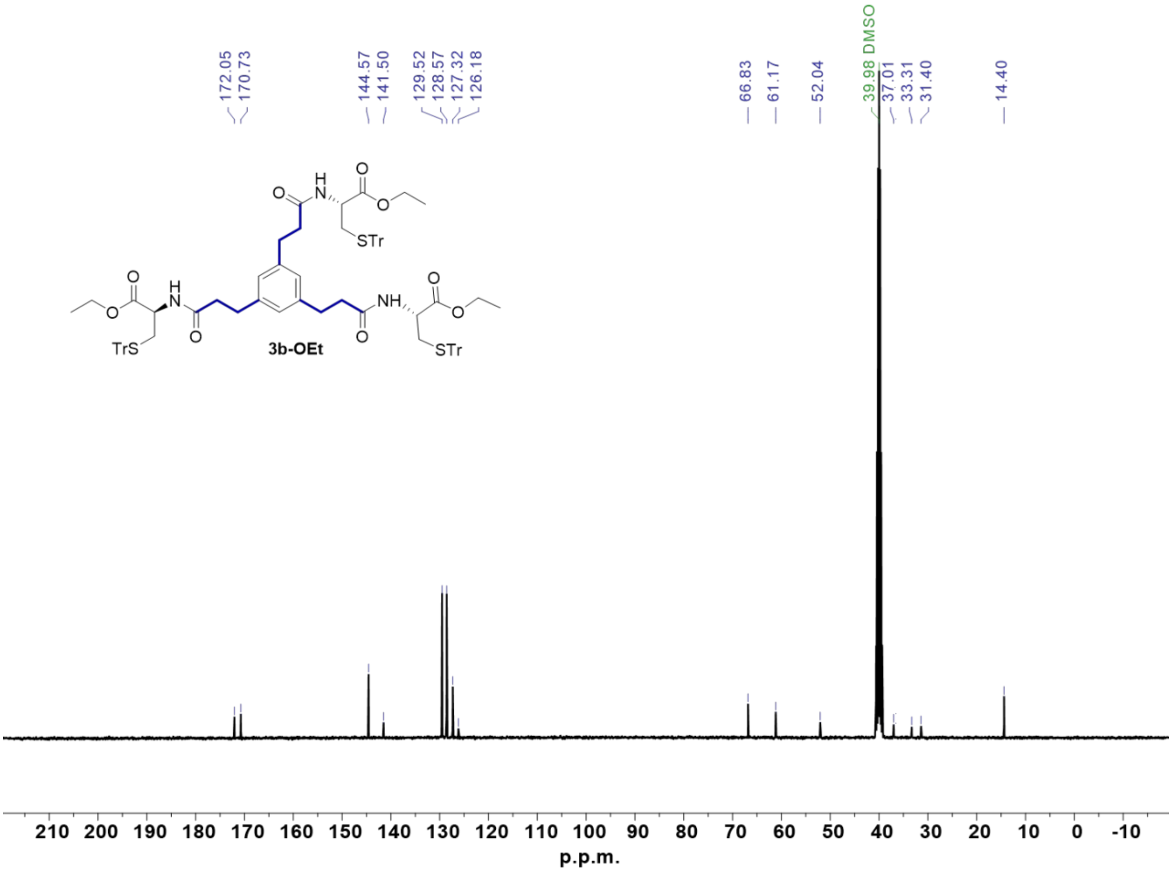

Fig. S12. $^{13}C\{^{1}H\}$ NMR (101 MHz, DMSO-d_6) of **3b-OEt**.

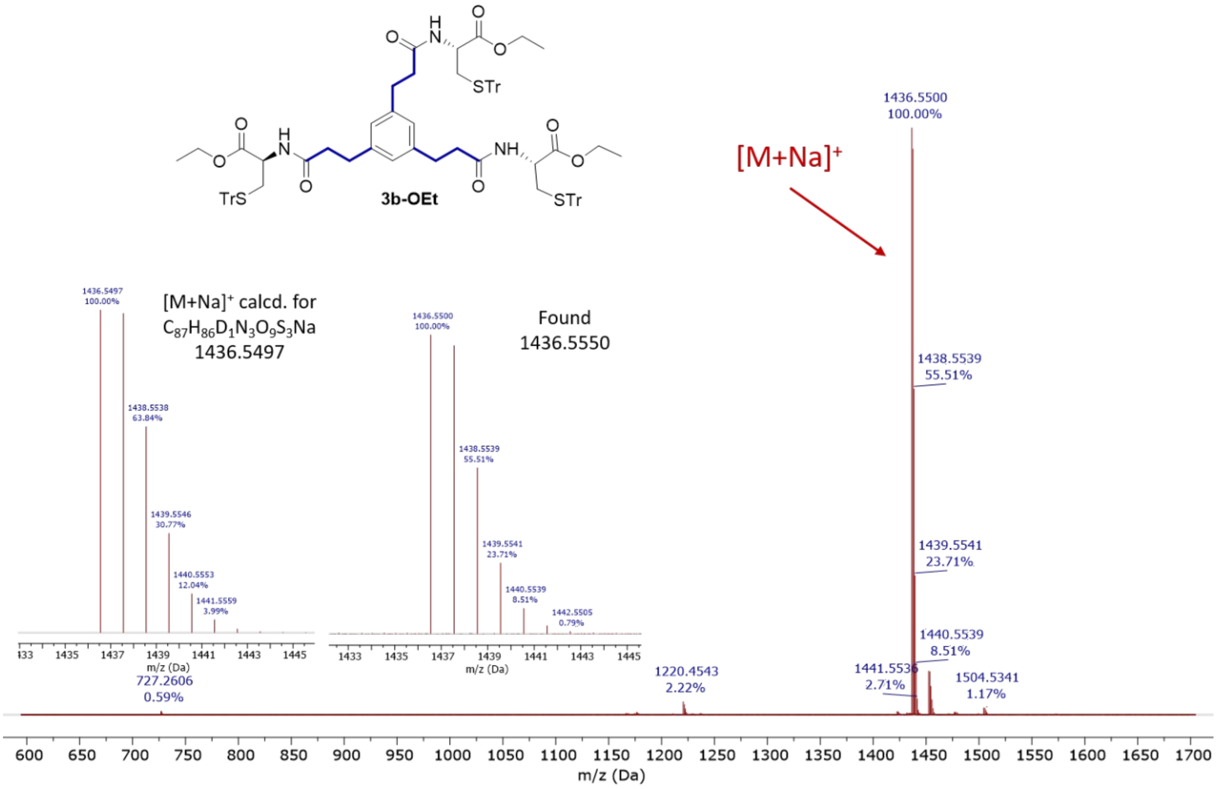

Fig. S13. ESI-MS of **3b-OEt**.

2.3. Synthesis of 3c

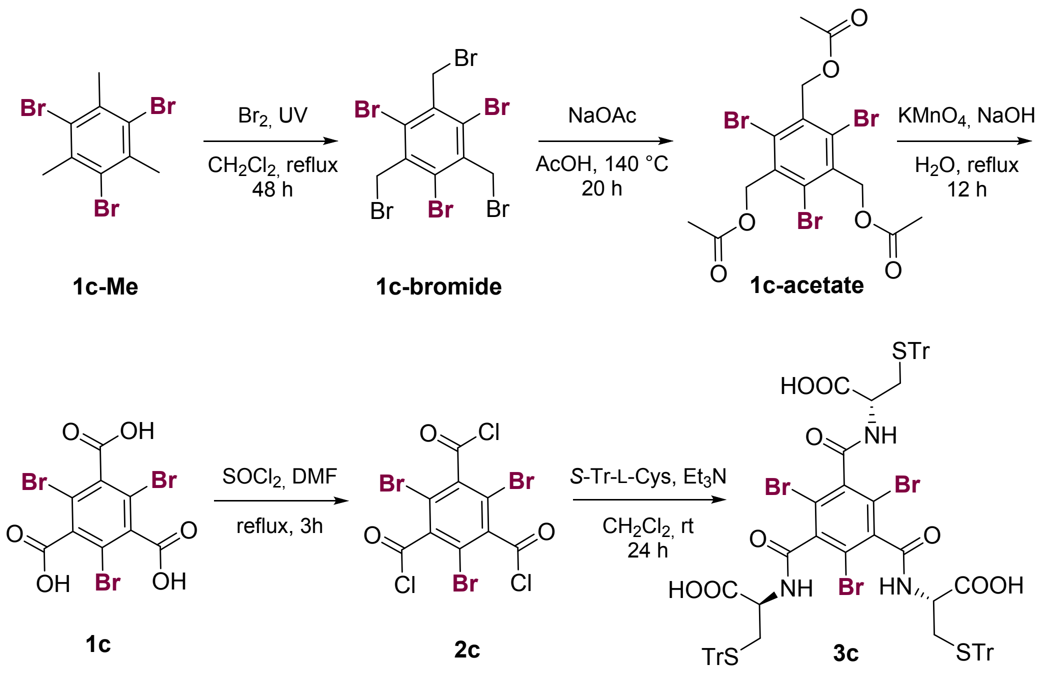

1c-bromide: The compound is known and was prepared according to a previously published procedure[4]. To the solution of 1,3,5-tribromo-2,4,6-trimethylbenzene (1.25 g, 3.5 mmol) in dichloromethane (30 mL) bromine (0.7 mL, 13.54 mmol) was added. Reaction mixture was refluxed for 48 hours in an oil bath at 40 °C while irradiating by UV lamp. After cooling the reaction mixture excess bromine and solvent were removed under the reduced pressure and crude product was dissolved in toluene-ethanol mixture (1:2) and filtered. Product was obtained as pale yellow crystals by slow crystallization in good 86% yield (1.8 g). **1c-bromide**: ^{1}H NMR (300 MHz, $CDCl_3$) δ 4.92 (s, 6H).

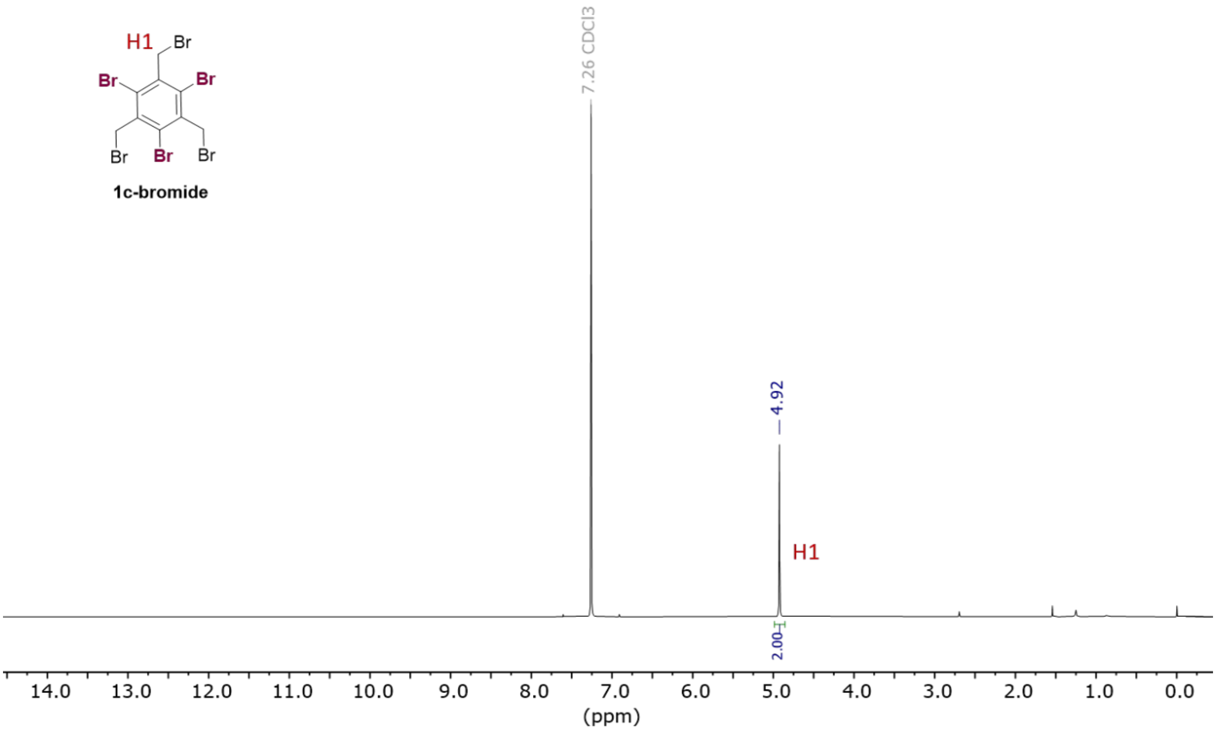

Fig. S14. ^{1}H NMR (300 MHz, $CDCl_3$) of **1c-bromide**.

1c-acetate: The compound is known and was prepared according to a previously published procedure[5]. To the solution of **1c-bromide** (890 mg, 1.5 mmol) in glacial acetic acid (20 mL) anhydrous sodium acetate (795 mg, 6.47 mmol) was added. Reaction mixture was stirred in Schlenk-type vessel at 140°C for 20 hours in an oil bath. Then, acetic acid was removed under the reduced pressure and the residue was dissolved in dichloromethane (50 mL). The solution was extracted by H_2O (50 mL), saturated sodium bicarbonate (50 mL) and brine (50 mL). Combined organic layers were dried over anhydrous Na_2SO_4, filtered and solvent was removed under reduced pressure to obtain crude product. Recrystallization from ethanol led to white crystals in good 88% yield (701 mg). **1c-acetate**: ^{1}H NMR (600 MHz, $CDCl_3$) δ 5.55 (s, 2H), 2.11 (s, 3H).

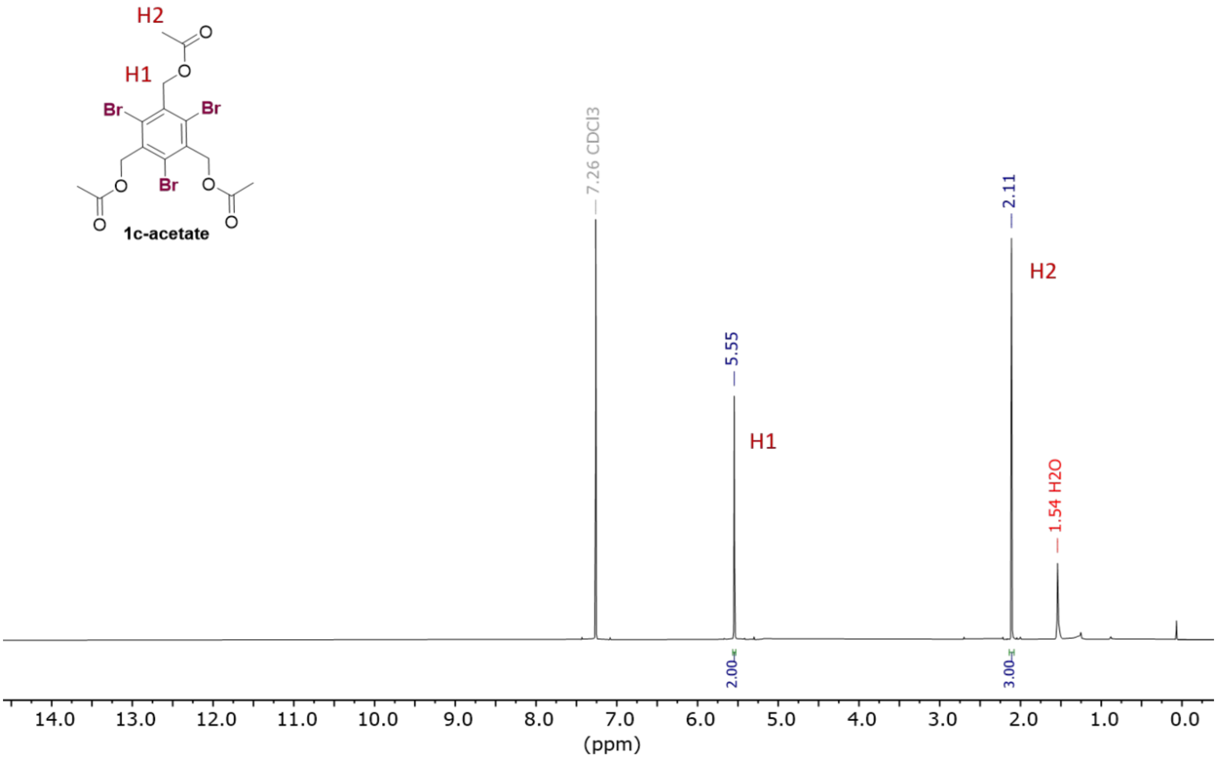

Fig. S15. ^{1}H NMR (600 MHz, $CDCl_3$) of **1c-acetate**.

1c: The compound is known and was prepared according to a previously published procedure[5]. To the suspension of **1c-acetate** (900 mg, 1.7 mmol) in H_2O (13.5 mL), potassium permanganate (1.2 g, 7.63 mmol) and 3M NaOH (4.5 mL) were added. Reaction mixture was refluxed for 12 hours in an oil bath at 100 °C. After cooling isopropanol was added (15 mL) to precipitate MnO_2, which was then filtered and washed with water (3 x 10 mL). Resulting solution was acidified to pH = 1 with 1M HCl and extracted with ethyl acetate (4 x 40 mL). Combined organic layers were washed with brine (80 mL), dried over anhydrous Na_2SO_4, filtered and concentrated. Product was obtained by precipitation with *n*-hexane as pale yellow solid in good 75% yield (570 mg). **1c**: ^{1}H NMR (600 MHz, DMSO-d_6) δ 14.51 (s, 1H), HRMS (TOF-MS) m/z: $[M+NH_4]^+$ calcd. for $C_9H_3O_6Br_3NH_4$ 463.7798, Found 463.7799.

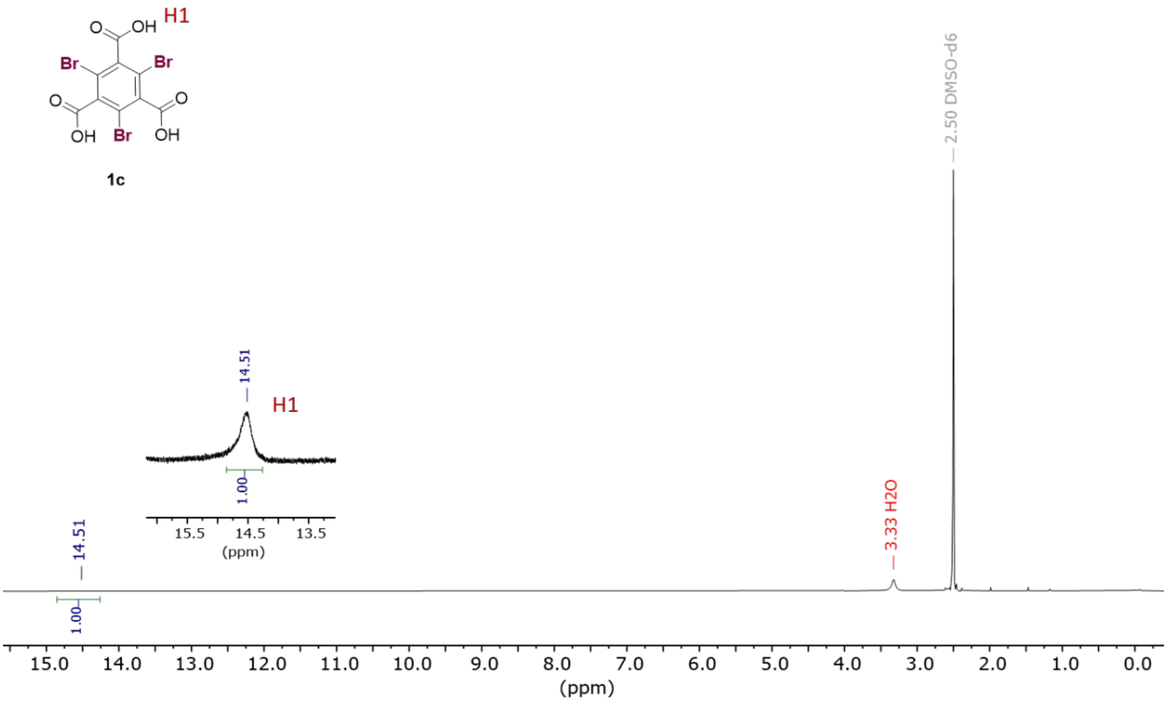

Fig. S16. ^{1}H NMR (600 MHz, DMSO-d_6) of **1c**.

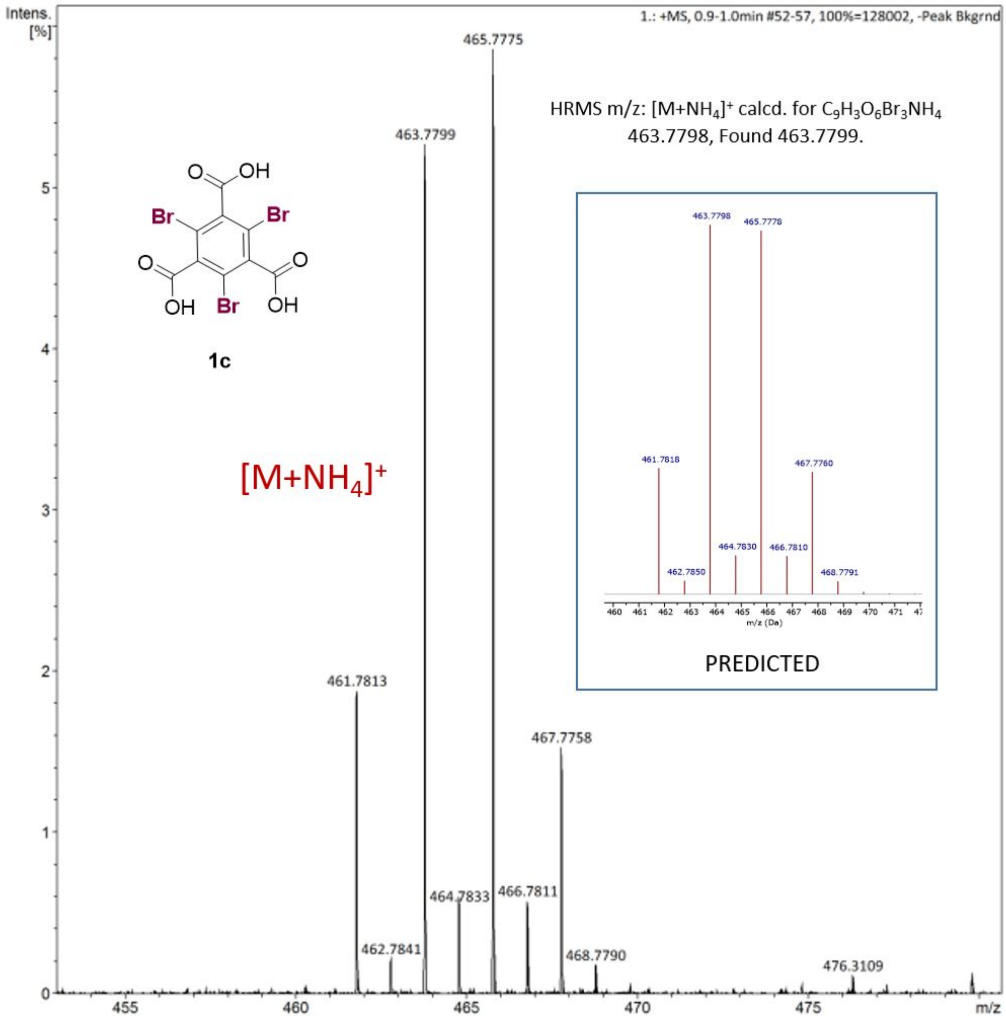

Fig. S17. ESI-MS of **1c**.

2c To the suspension of **1c** (150 mg, 0.34 mmol) in SOCl2 (15 mL) catalytic amount of DMF (*N,N-dimethylformamide* , 1 drop) was added. Reaction mixture was refluxed for 3 hours in an oil bath at 80 °C. After, $SOCl_2$ was removed under the reduced pressure and the crude product was used in the next reaction.

3c To the suspension of *S*-Trityl-l-cysteine (727 mg, 2 mmol) in dichloromethane (15 mL) Et_3N (Triethylamine, 0.27 ml, 2 mmol) was added, followed by **2c** prepared by previous reaction. Resulting solution was stirred at ambient temperature in Ar atmosphere for 24 hours. Solvent was removed under reduced pressure and resulting oil was dissolved in acetone (10 mL), which was then added dropwise to vigorously stirring 1M HCl (100 mL). White precipitate was collected by filtration, washed with water (3 x 15 mL) and dried in vacuum. Product was isolated in 80% yield (402 mg) as a white amorphous solid.

^{1}H NMR (600 MHz, DMSO-d_6) δ 12.94 (s, 1H), 9.07 (d, *J* = 36.3 Hz, 1H), 7.26 (d, *J* = 30.1 Hz, 15H), 4.31 (d, *J* = 6.0 Hz, 1H), 2.48 (m, 2H).

^{13}C{^{1}H} NMR (151 MHz, DMSO-d_6) δ 170.8, 164.2, 144.2, 139.8, 129.1, 128.1, 127.7, 126.8, 66.3, 51.6, 32.9.

HRMS (TOF-MS) m/z: $[M+Na]^+$ calcd. for $C_{75}H_{60}Br_3N_3O_9S_3Na$ 1506.0914, Found 1506.0918.

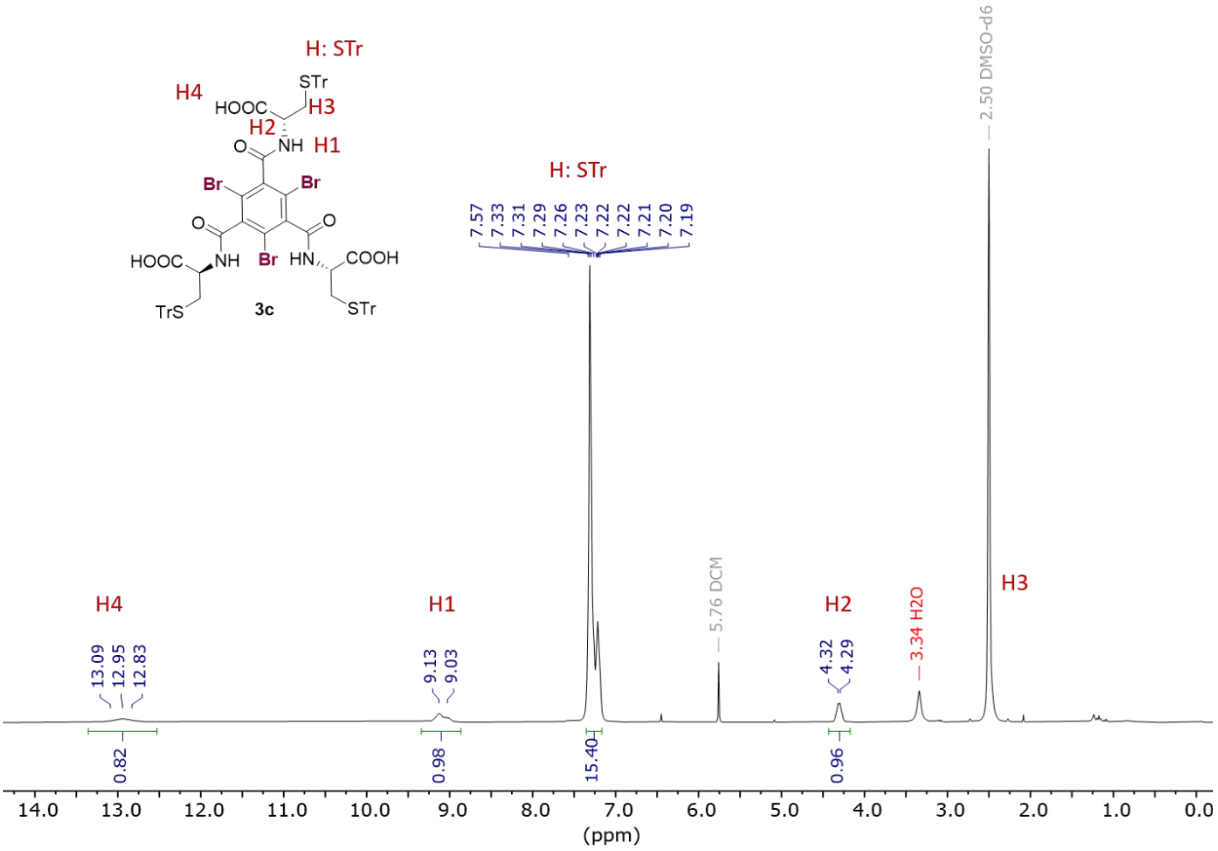

Fig. S18. ^{1}H NMR (600 MHz, DMSO-d_6) of **3c**.

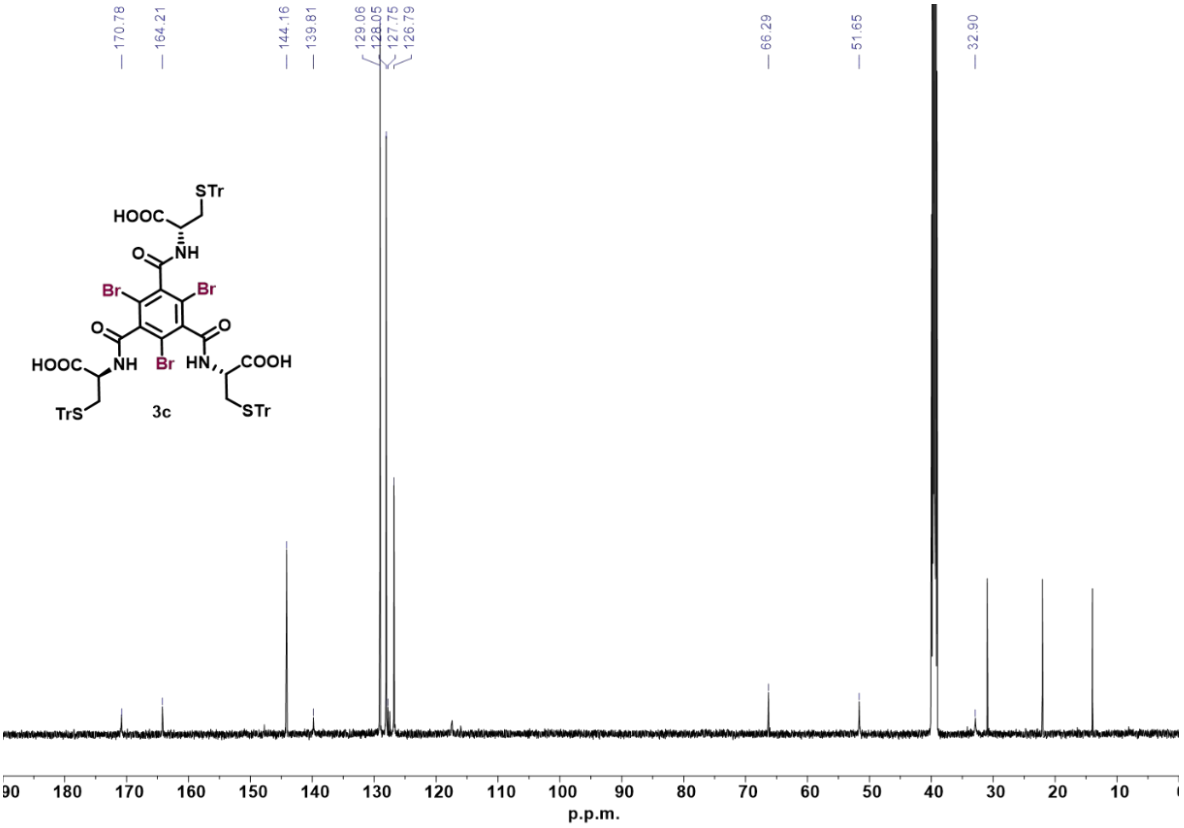

Fig. S19. $^{13}C\{^{1}H\}$ NMR (151 MHz, DMSO-d_6) of **3c**.

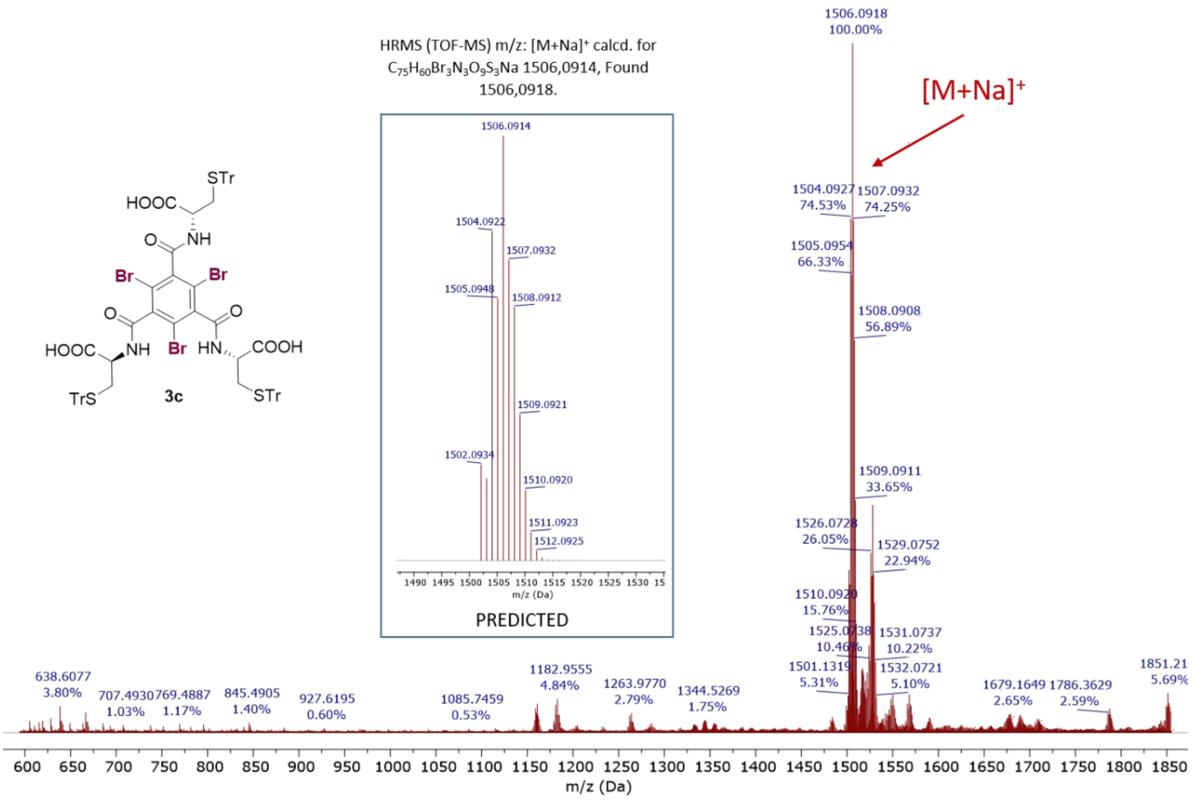

Fig. S20. ESI-MS of **3c**.

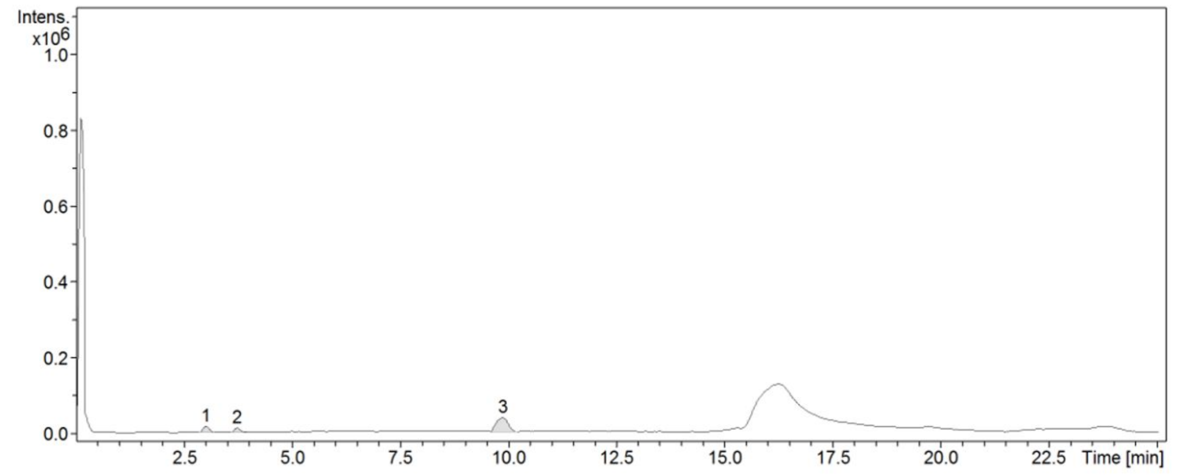

#	RT [min]	Area	Area %	Area Frac. %	I	Int. %	Max. m/z
1	3.0	155338	22.50	16.45	21359	48.87	1156.0128
2	3.7	98664	14.29	10.45	16427	37.58	1259.0219
3	9.9	690431	100.00	73.11	43707	100.00	1484.1055

$[M\text{-}Tr\text{-}2Ph+K]^+$ $C_{46}H_{42}Br_3N_3O_9S_3K$
$[M\text{-}Tr+NH_4]^+$ $C_{56}H_{46}Br_3N_3O_9S_3NH_4$
$[M+H]^+$ $C_{75}H_{60}Br_3N_3O_9S_3$

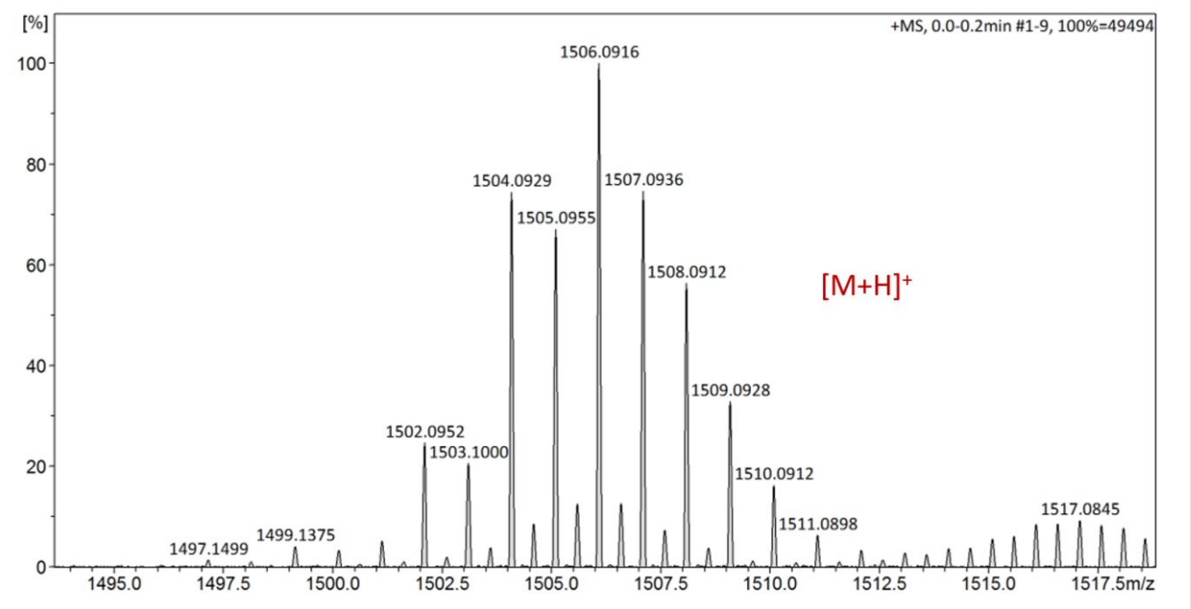

Fig. S21. LC-MS of **3c**. Under LC-MS conditions, compound **3c** undergoes fragmentation.

2.4. Synthesis of 3d

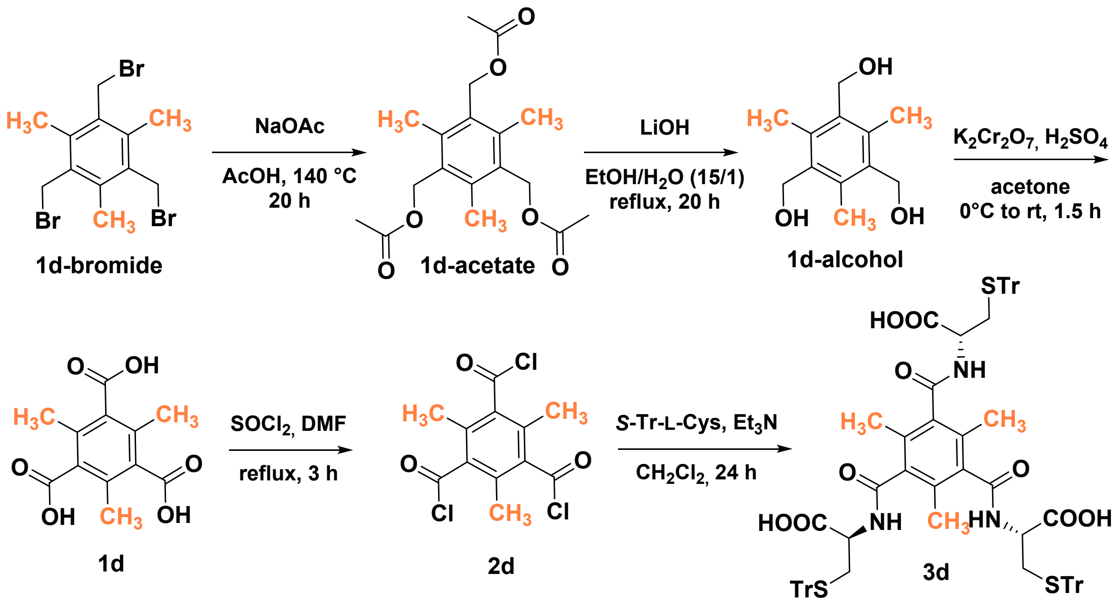

1d-acetate: The compound is known and was prepared according to a previously published procedure[6]. To the solution of **1d-bromide** (850 mg, 2.13 mmol) in glacial acetic acid (20 mL) anhydrous sodium acetate (1 g, 12.2 mmol) was added. Reaction mixture was heated to 140 °C for 20 hours in an oil bath. After, the mixture was concentated under the reduced pressure and water was added to precipitate the product. **1d-acetate** was collected by fitration as a white amorphous solid in good 91% yield (650 mg). **1d-acetate**: ^{1}H NMR (600 MHz, $CDCl_3$) δ 5.24 (s, 1H), 2.41 (s, 1H), 2.07 (s, 1H).

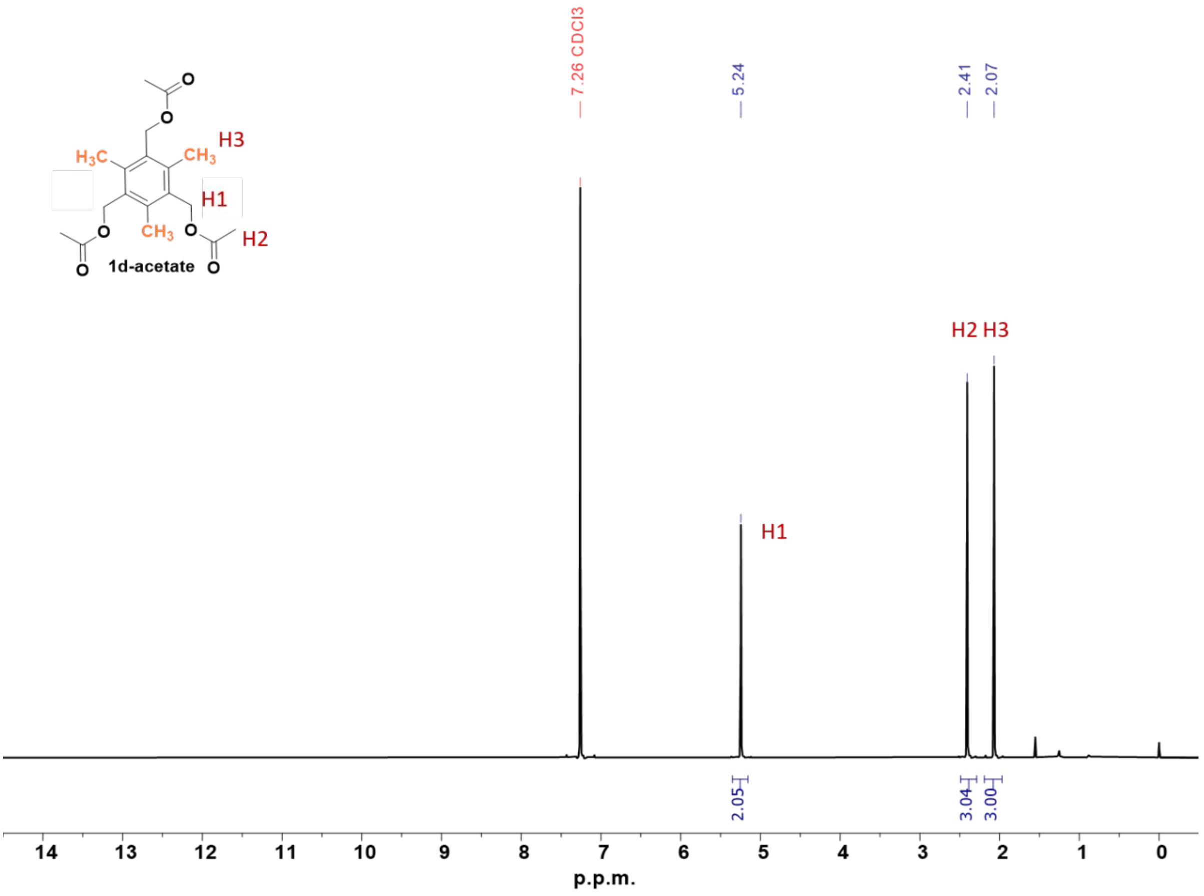

Fig. S22. ^{1}H NMR (600 MHz, $CDCl_3$) of **1d-acetate**.

1d-alcohol: The compound is known and was prepared according to a previously published procedure[7]. To the suspension of **1d-acetate** (1.2 g, 3.57 mmol) in EtOH (45 mL) LiOH solution in water (344 mg in 3 mL) was added. Reaction mixture was heated to reflux for 20 hours in an oil bath. After, EtOH was removed under reduced pressure and solid residue was filtered and washed thoroughly with water. Product was collected as a white amorphous solid in excellent 95% yield (600 mg). **1d-alcohol**: ^{1}H NMR (300 MHz, DMSO-d_6) δ 4.63 (t, *J* = 4.9 Hz, 1H), 4.50 (d, *J* = 4.9 Hz, 2H), 2.37 (s, 3H).

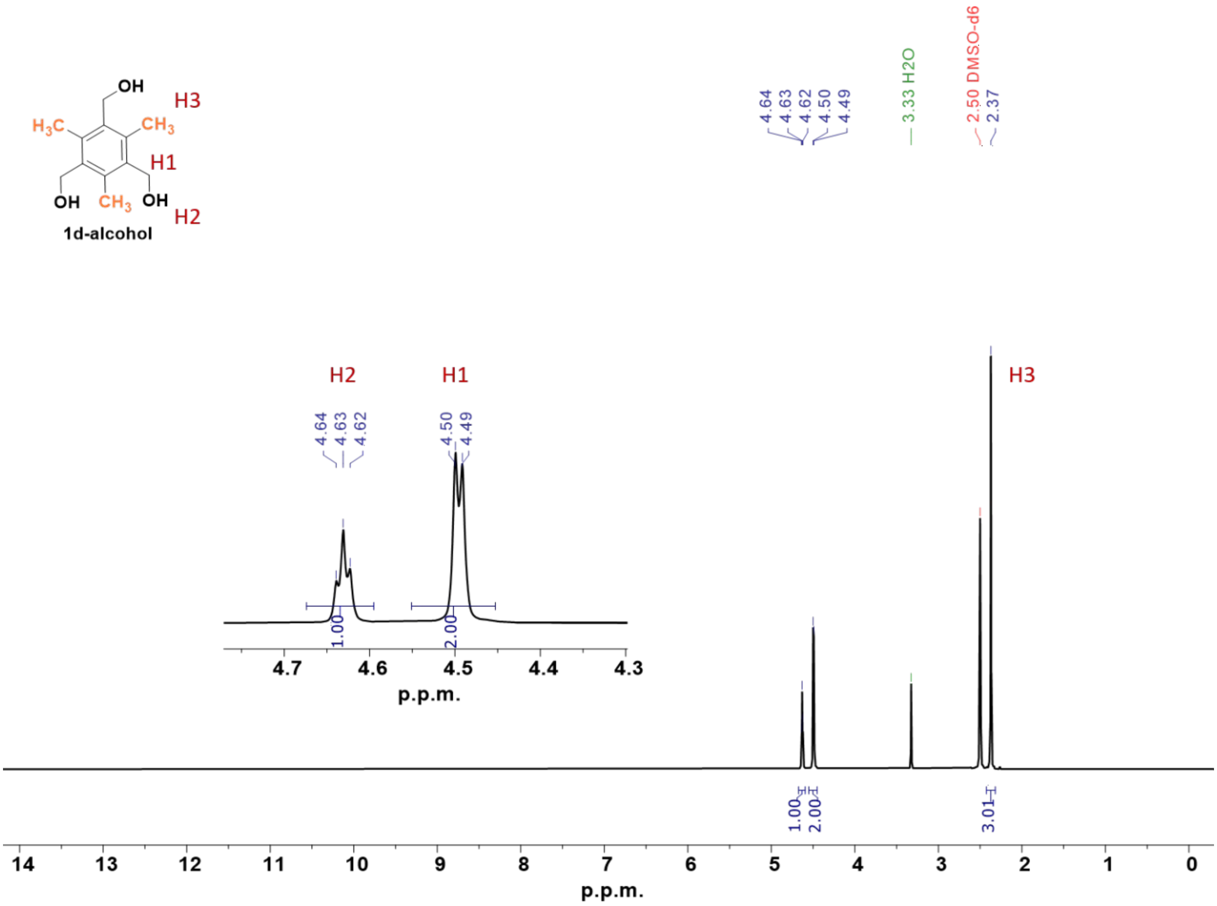

Fig. S23. ^{1}H NMR (300 MHz, DMSO-d_6) of **1d-alkohol**.

1d: The compound is known and was prepared according to a previously published procedure[6]. Solution of **1d-alcohol** (525 mg, 2.5 mmol) in acetone (30 mL) was cooled to 0 °C, at this temperature solution of CrO_3 (2 g) in H_2SO_4 (2 mL) and H_2O (6 mL) was added dropwise. Reaction mixture was stirred for 30 minutes at 0°C and 30 minutes at ambient temperature. After the reaction mixture was diluted with H_2O (75 mL) and extracted with Et2O (4 x 25 mL). Combined organic layers were dried over Na_2SO_4, solvent was removed under reduced pressure and the residue was recrystalized from hot acetonitrile. Product was collected as white needles in safisfactory 42% yield (265 mg). **1d**: ^{1}H NMR (300 MHz, DMSO-d_6) δ 13.49 (s, 1H), 2.20 (s, 3H), HRMS (TOF-MS) m/z: $[M+NH_4]^+$ calcd. for $C_{12}H_{12}O_6NH_4$ 270.0972, Found 270.0972.

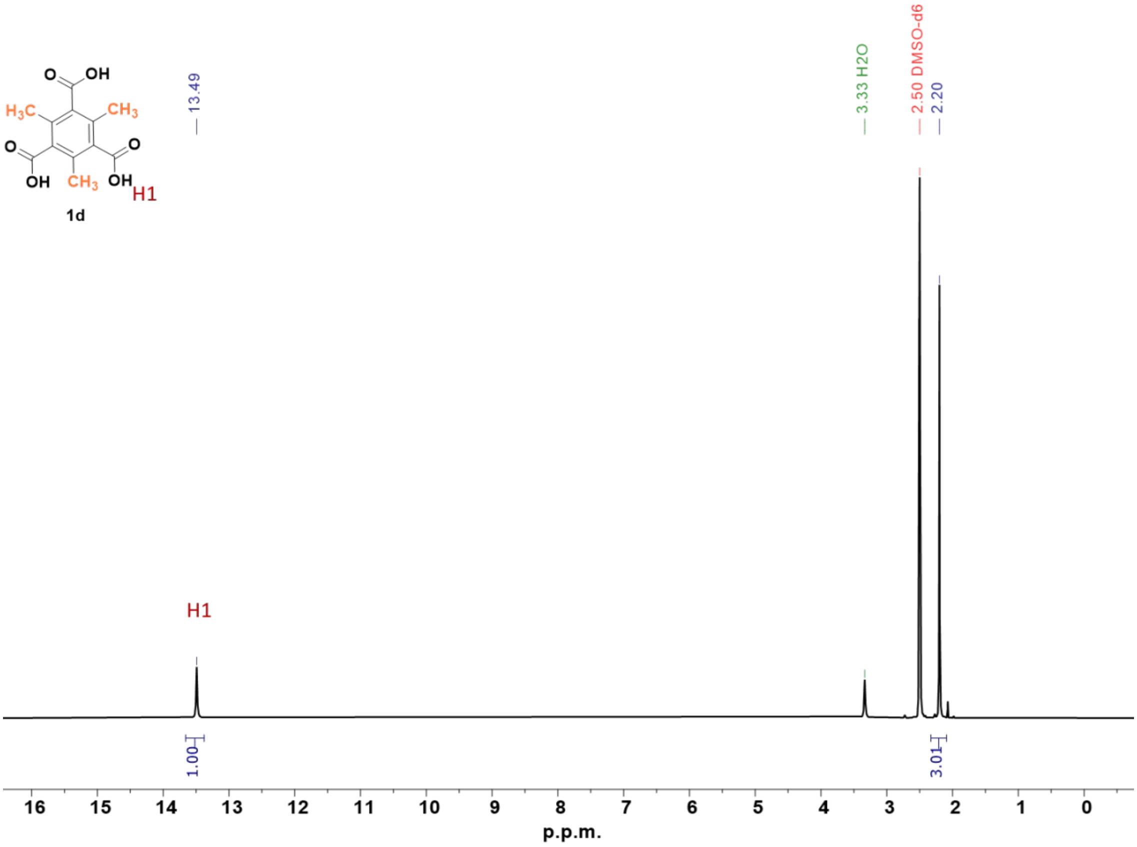

Fig. S24. ^{1}H NMR (300 MHz, DMSO-d_6) of **1d**.

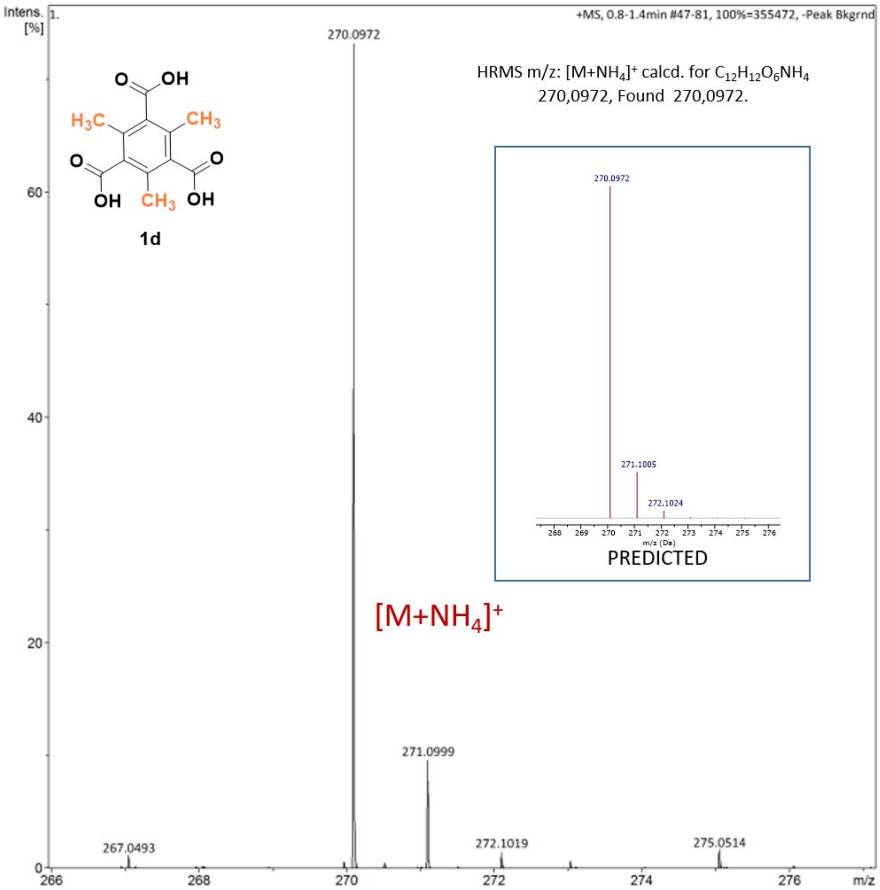

Fig. S25. ESI-MS of **1d**.

2d: To the suspension of **1d** (86 mg, 0.34 mmol) in SOCl2 (15 mL) catalytic amount of DMF (*N,N-dimethylformamide* , 1 drop) was added. Reaction mixture was refluxed for 3 hours in an oil bath at 80 °C. After, $SOCl_2$ was removed under the reduced pressure and the crude product was used in the next reaction.

3d: To the suspension of *S*-Trityl-l-cysteine (727 mg, 2 mmol) in dichloromethane (15 mL) Et_3N (Triethylamine, 0.27 ml, 2 mmol) was added, followed by **2d** prepared by previous reaction. Resulting solution was stirred at ambient temperature in Ar atmosphere for 24 hours. Solvent was removed under reduced pressure and resulting oil was dissolved in acetone (10 mL), which was then added dropwise to vigorously stirring 1M HCl (100 mL). White precipitate was collected by filtration, washed with water (3 x 15 mL) and dried in vacuum. Product was isolated in 62% yield (271 mg) as a white amorphous solid.

^{1}H NMR (600 MHz, DMSO-d_6) δ 12.76 (s, 1H), 8.57 (s, 1H), 7.56 – 7.00 (m, 18H), 4.22 (tt, *J* = 9.1, 4.3 Hz, 1H), 2.64 (q, *J* = 9.9, 8.0 Hz, 1H), 2.14 (d, *J* = 3.8 Hz, 3H).

^{13}C{1H} NMR (151 MHz, DMSO-d_6) δ 171.5, 168.5, 144.2, 136.0, 130.4, 129.0, 128.1, 126.8, 66.2, 51.4, 32.7, 16.1.

HRMS (TOF-MS) m/z: $[M+H]^+$ calcd. for $C_{78}H_{69}N_3O_9S_3H$ 1288.4269, Found 1288.4311.

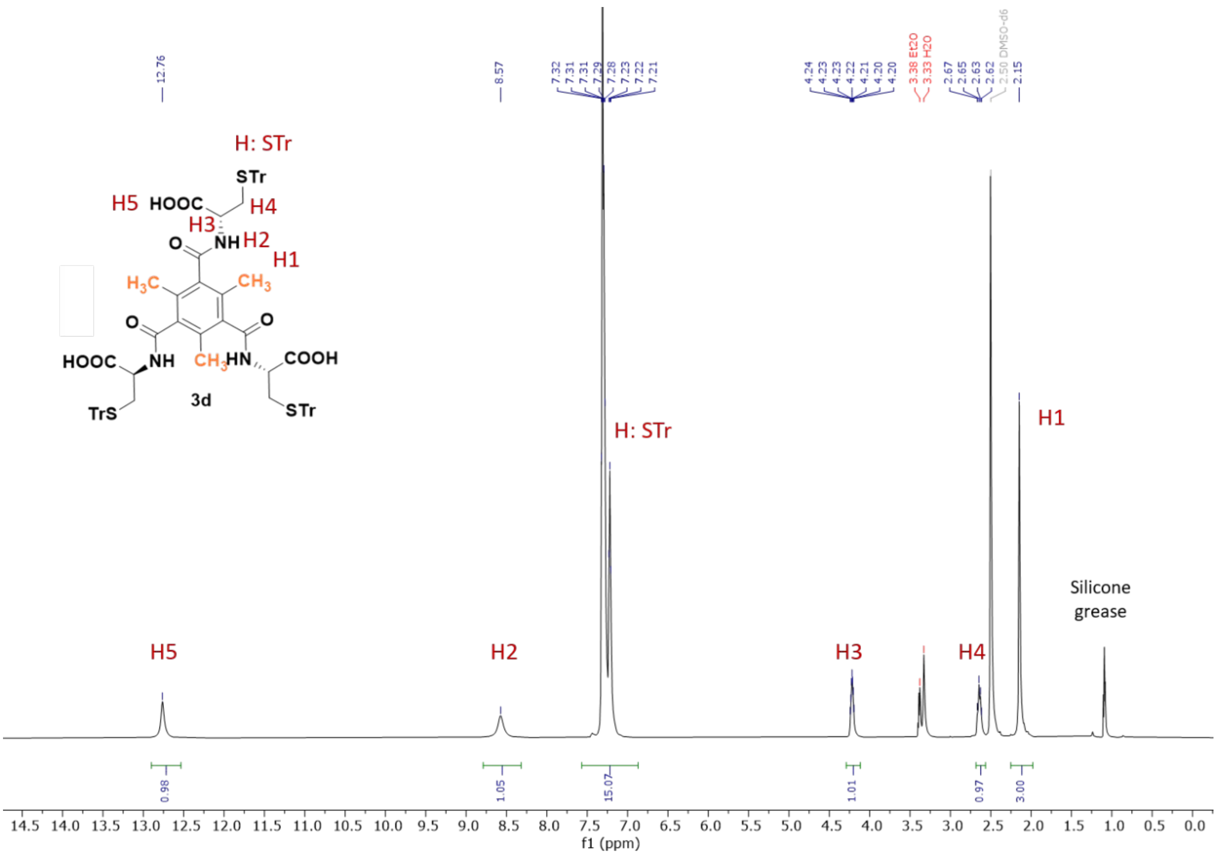

Fig. S26. ^{1}H NMR (600 MHz, DMSO-d_6) of **3d**.

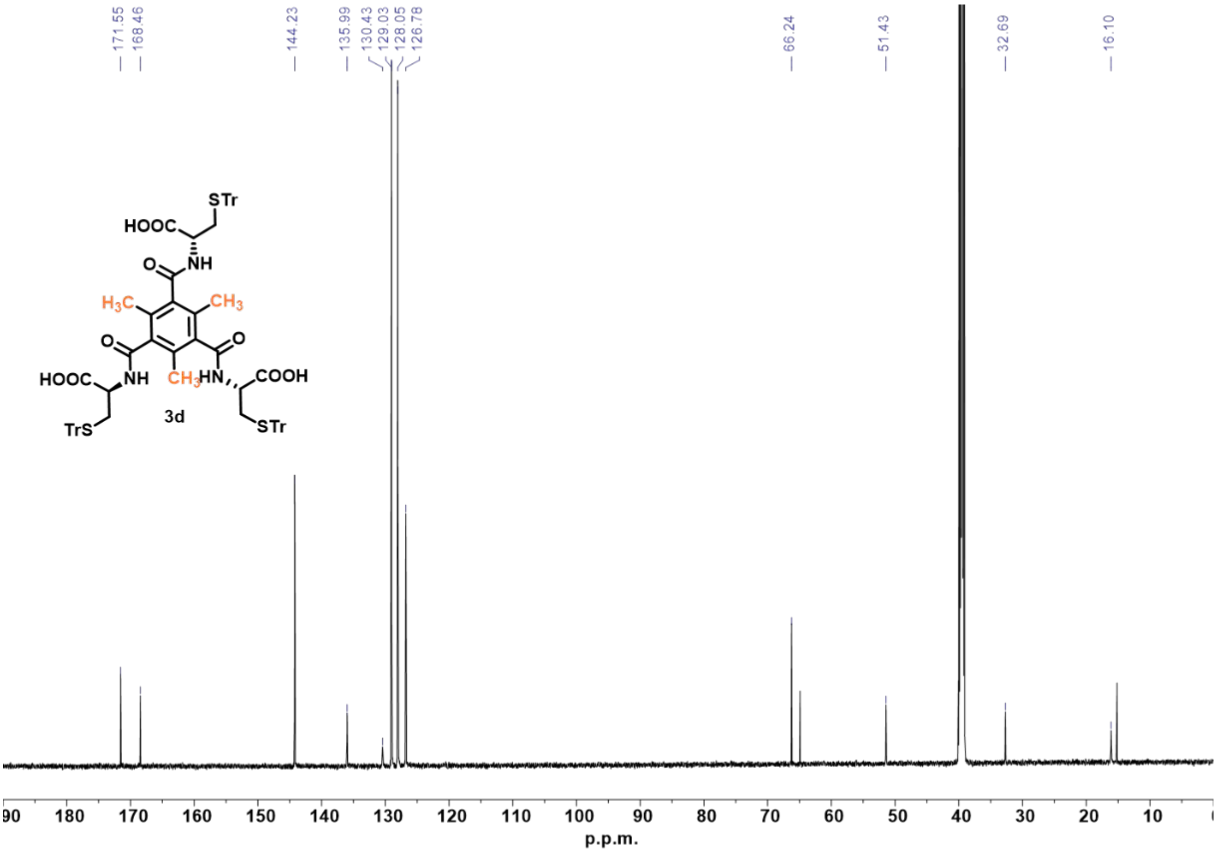

Fig. S27. $^{13}C\{^1H\}$ NMR (151 MHz, DMSO-d_6) of **3d**.

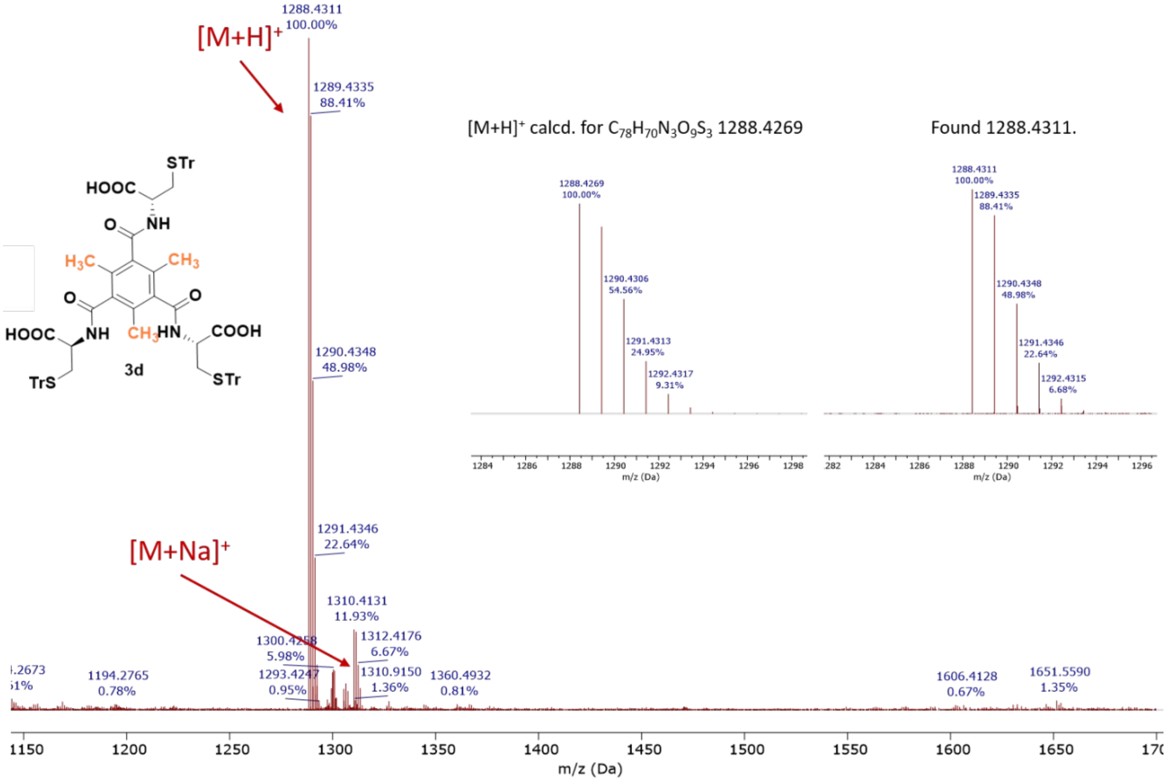

Fig. S28. ESI-MS of **3d**.

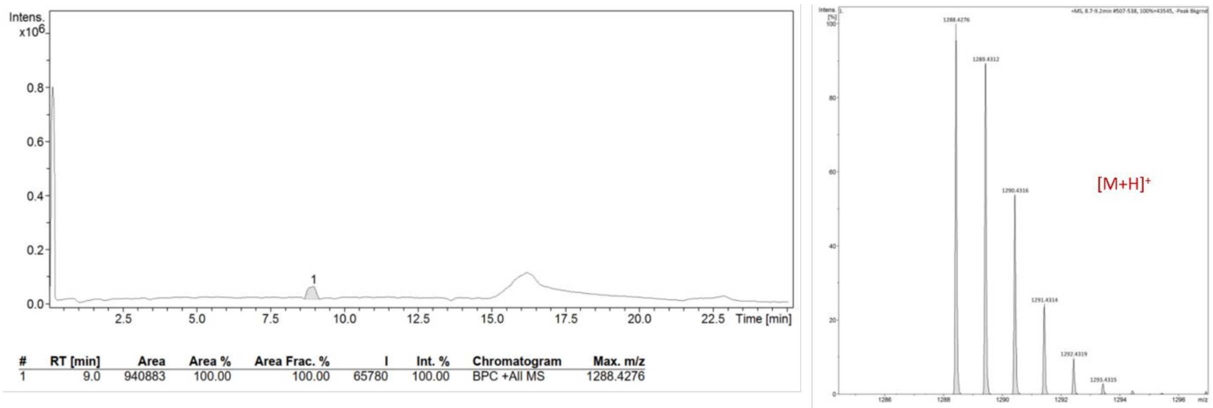

Fig. S29. LC-MS of **3d**.

2.5. Synthesis of 3e

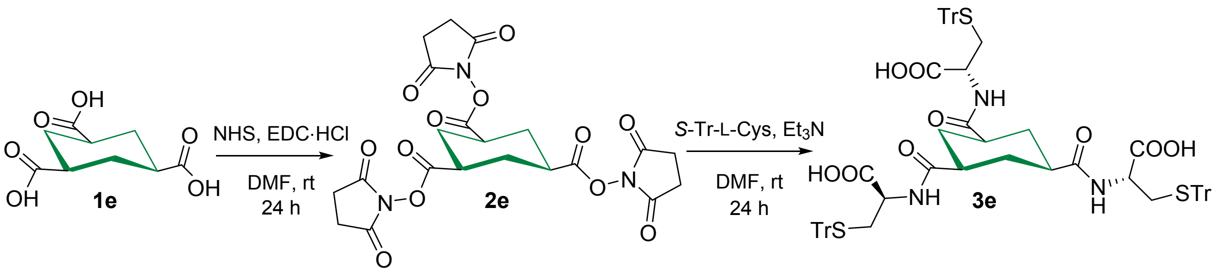

2e: The compound is known and was prepared according to a previously published procedure[8]. To the solution of cyclohexane-1,3,5-tricarboxylic acid (216 mg, 1 mmol) in anhydrous DMF (7 mL) 1-Ethyl-3-(3-dimethylaminopropyl)carbodiimide (1.15 g, 6 mmol) and *N*-hydroxysuccinimide (690 mg, 6 mmol) was added. Resulting solution was stirred for 24 hours at ambient temperature in Ar atmosphere. After, solvent was removed under reduced pressure and the resulting oil was dissolved in acetone (15 mL), which was then added dropwise to vigorously stirring 1M HCl (100 mL). White precipitate was collected by filtration, washed with water (3 x 15 mL) and hot isopropanol (15 mL) to obtain the product in good 85% yield (430 mg) as a white amorphous. **2e**: ^{1}H NMR (300 MHz, DMSO-d_6) δ 3.32 – 3.13 (m, 1H), 2.82 (s, 4H), 2.36 (d, J = 12.9 Hz, 1H), 1.73 (q, J = 12.6 Hz, 1H).

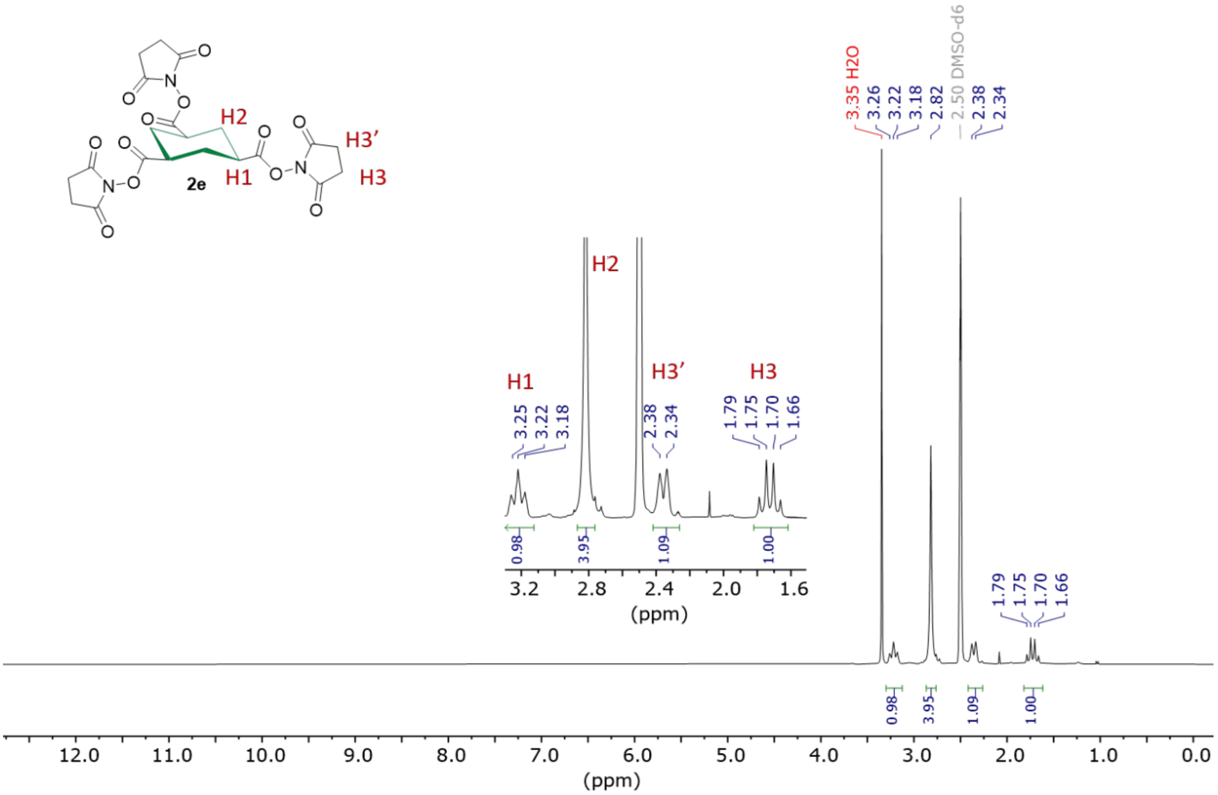

Fig. S30. ^{1}H NMR (300 MHz, DMSO-d_6) of **2e**

3e: To the suspension of *S*-Trityl-l-cysteine (1.09 mg, 3 mmol) in DMF (20 mL) Et_3N (0.41 ml, 3 mmol) was added, followed by **2e** (253 mg, 0.5 mmol). Resulting solution was stirred at ambient temperature in Ar atmosphere for 24 hours. Solvent was removed under reduced pressure and resulting oil was dissolved in acetone (15 mL), which was then added dropwise to vigorously stirring 1M HCl (100 mL). White precipitate was collected by filtration, washed with water (3 x 20 mL) and dried in vacuum. Product was isolated in 69% yield (430 mg) as a white amorphous.

^{1}H NMR (600 MHz, DMSO-d_6) δ 12.66 (s, 1H), 8.17 (d, *J* = 8.2 Hz, 1H), 7.57 – 6.97 (m, 15H), 4.13 (q, *J* = 7.4 Hz, 1H), 2.37 (d, *J* = 7.8 Hz, 2H), 2.30 (t, *J* = 12.9 Hz, 1H), 1.73 (d, *J* = 12.1 Hz, 1H), 1.39 (q, *J* = 12.6 Hz, 1H).

^{13}C{1H} NMR (151 MHz, DMSO-d_6) δ 174.2, 171.7, 144.2, 129.0, 128.1, 126.8, 66.1, 51.1, 42.2, 33.0, 31.2.

HRMS (TOF-MS) m/z: [M+Na]$^+$ calcd. for $C_{75}H_{69}N_3O_9S_3Na$ 1274.4088, Found 1274.4090.

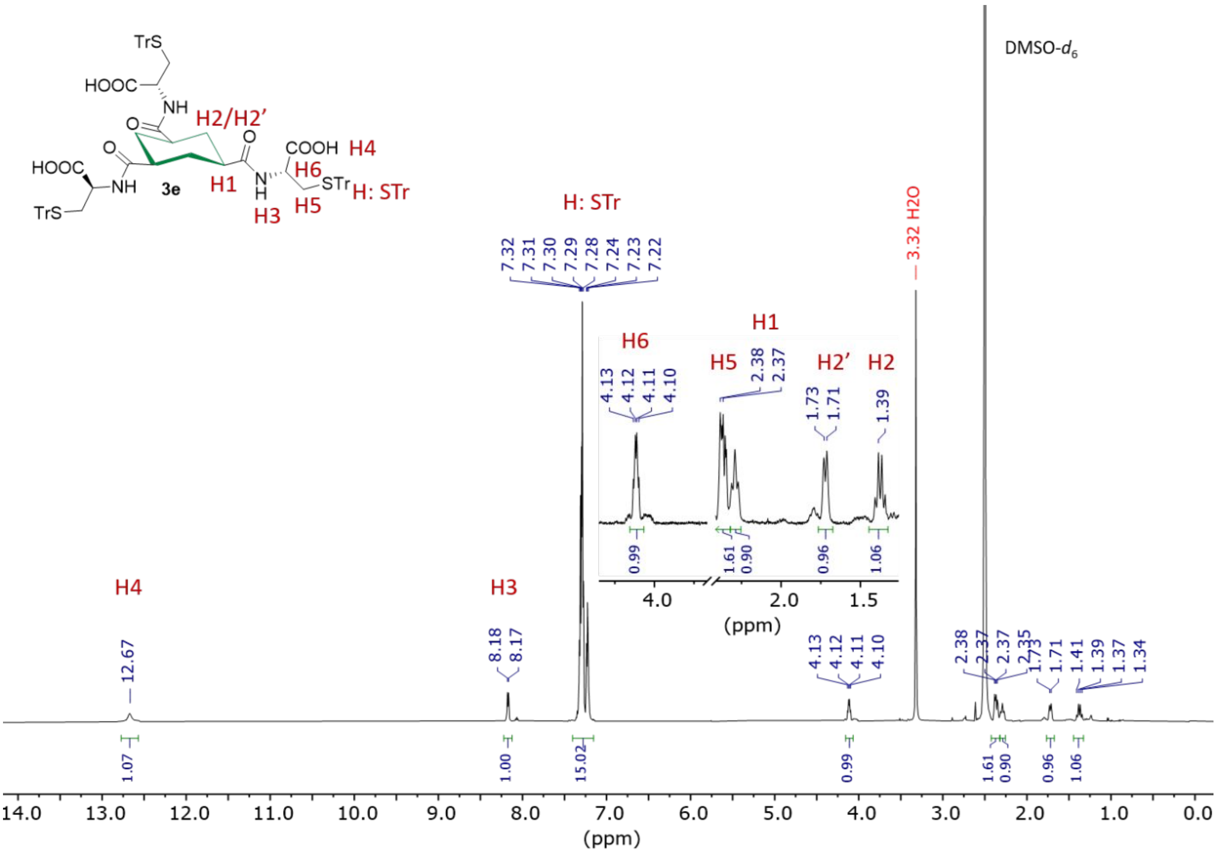

Fig. S31. ^{1}H NMR (600 MHz, DMSO-d_6) of **3e**.

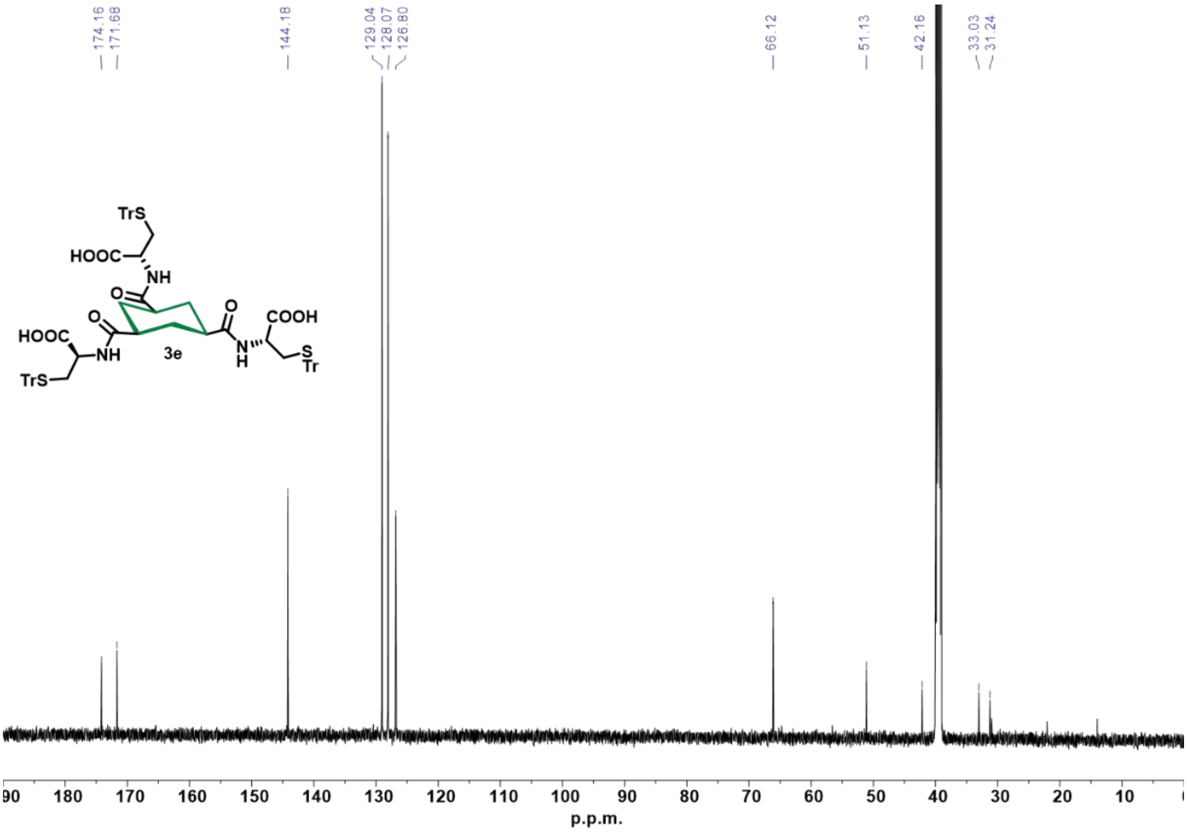

Fig. S32. $^{13}C\{^{1}H\}$ NMR (151 MHz, DMSO-d_6) of **3e**.

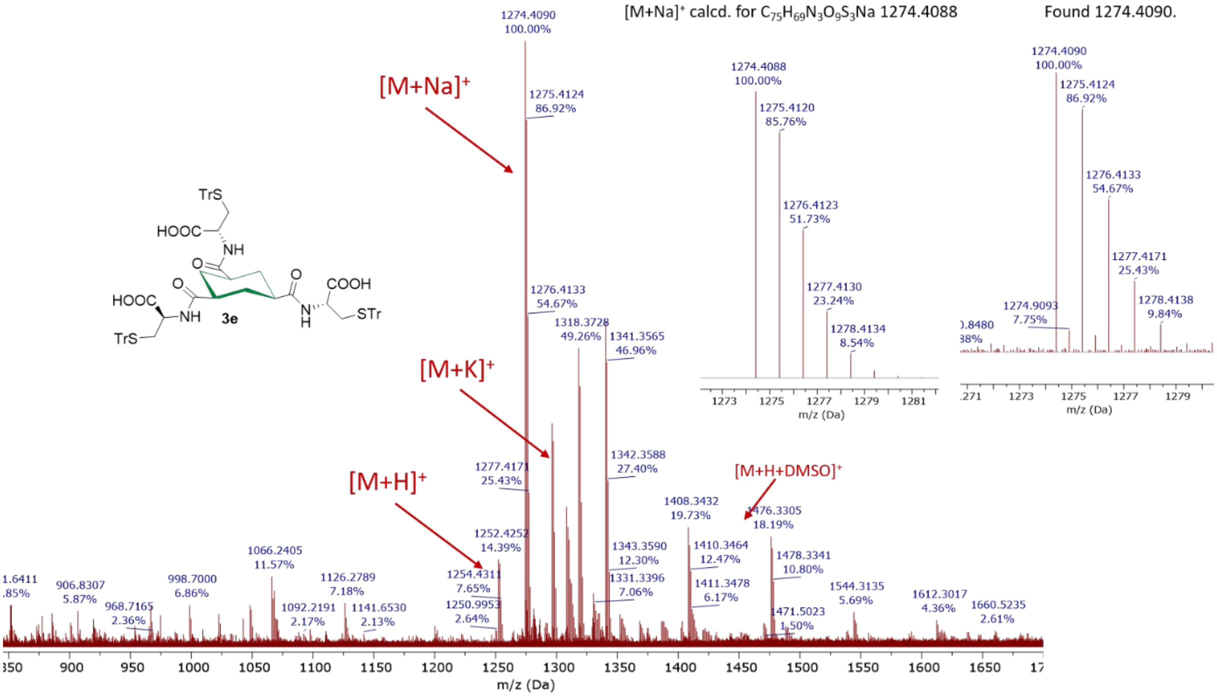

Fig. S33. ESI-MS of **3e**.

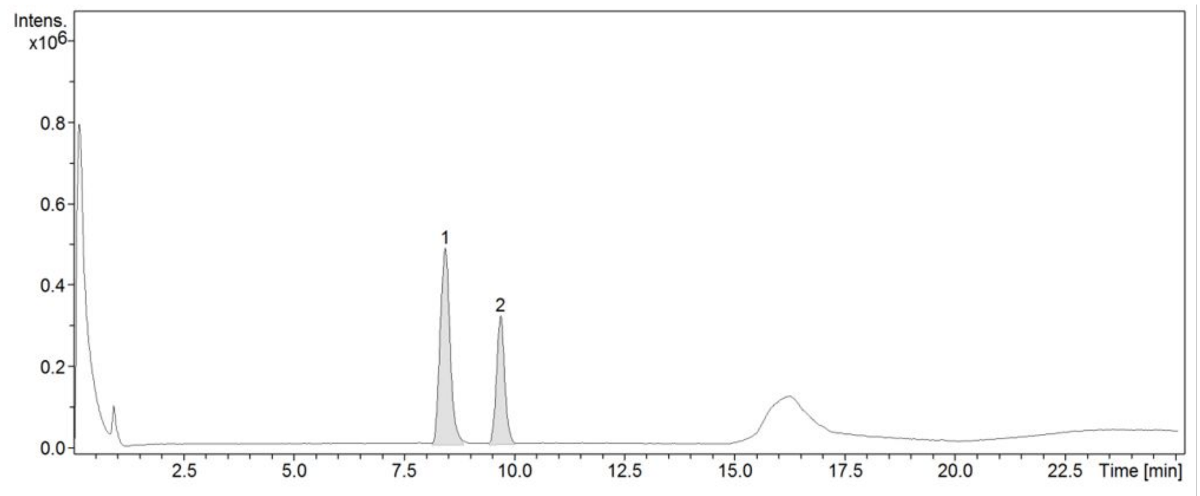

#	RT [min]	Area	Area %	Area Frac. %	I	Int. %	Chromatogram	Max. m/z
1	8.4	7479837	100.00	64.58	489725	100.00	BPC +All MS	1252.4246
2	9.7	4103067	54.86	35.42	324693	66.30	BPC +All MS	1252.4227

Two Isomeric forms

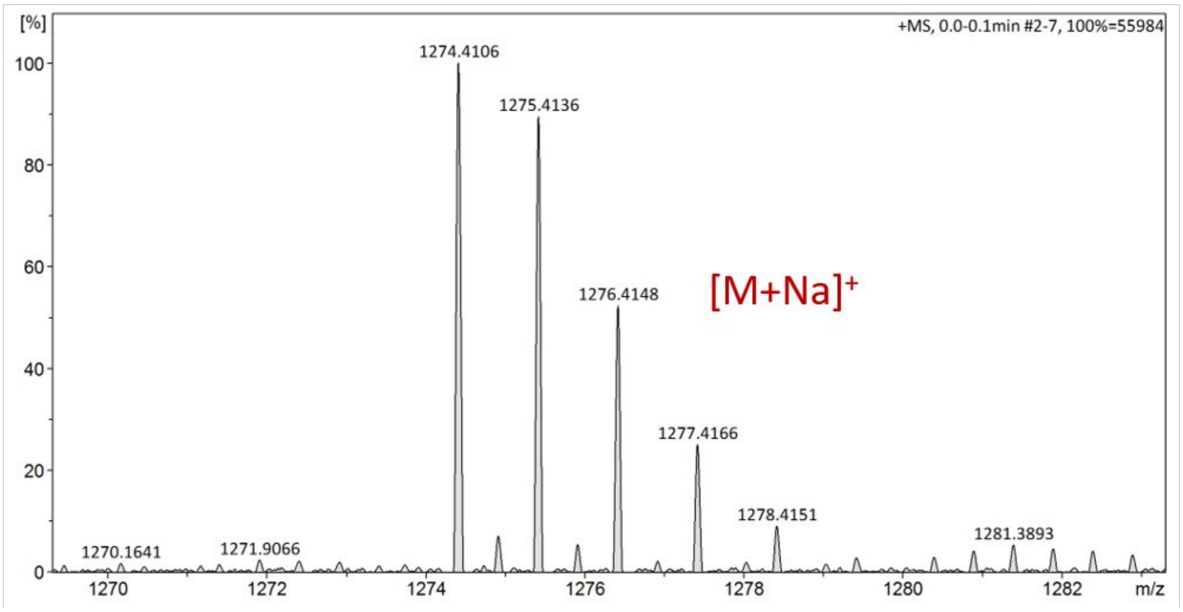

Fig. S34. LC-MS of **3e**. The LC-MS chromatogram of compound 3e shows two peaks corresponding to its isomeric forms.

2.6. DOSY NMR

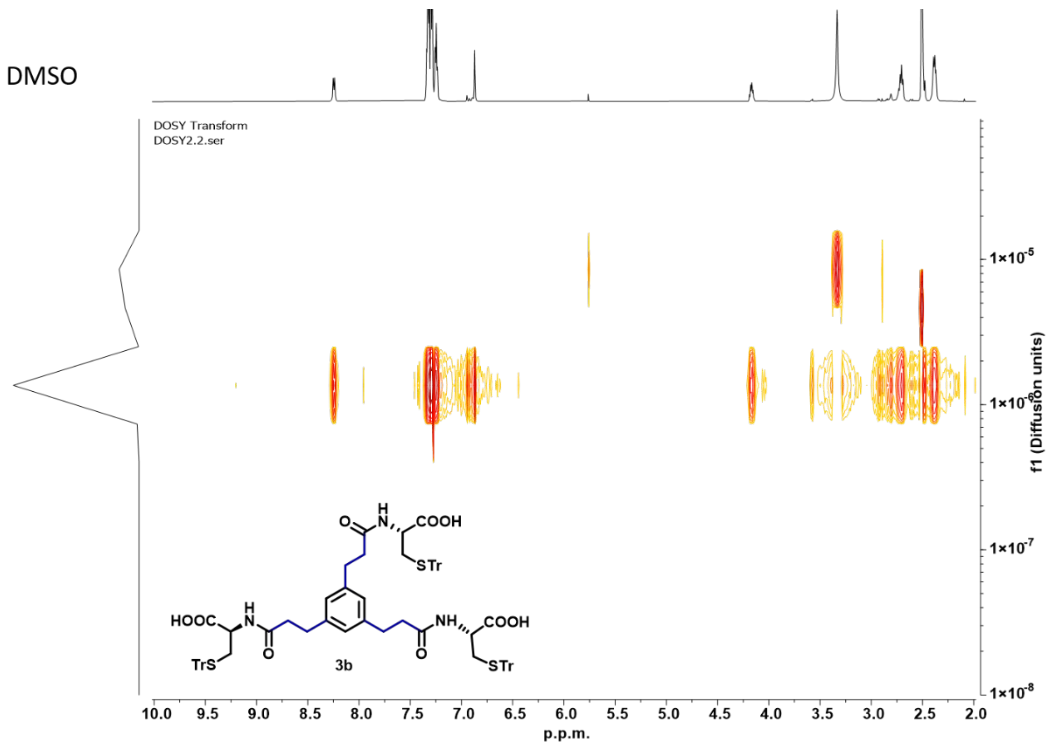

Fig. S35. ^{1}H DOSY NMR (600 MHz, DMSO-d$_6$) of **3b**.

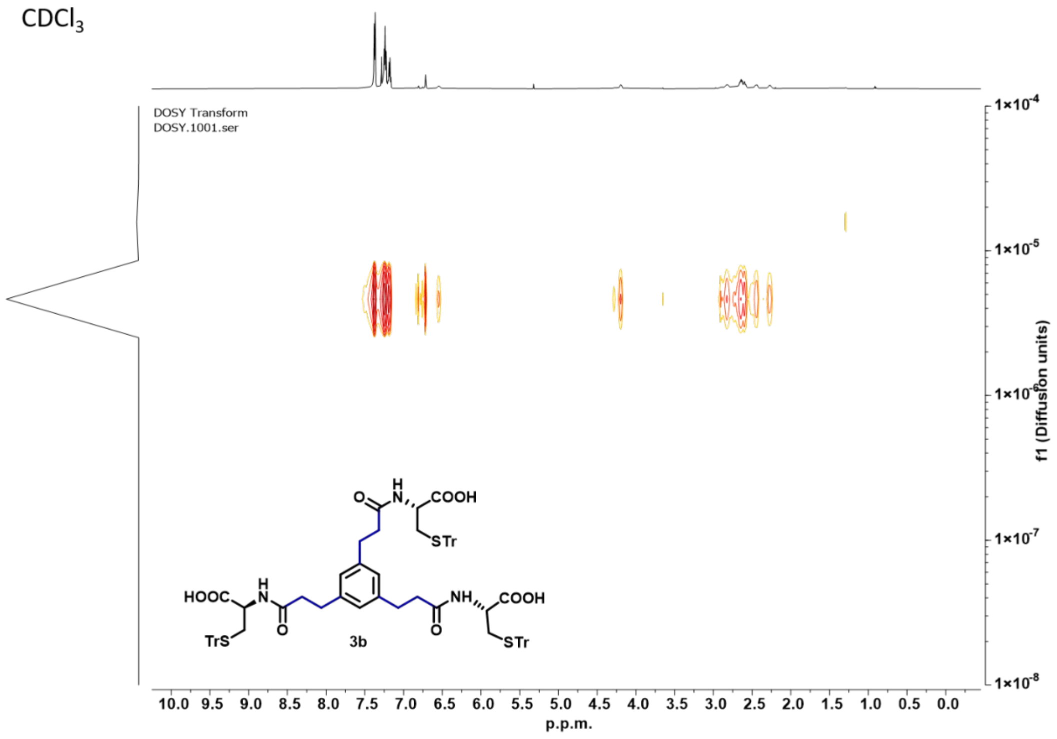

Fig. S36. ^{1}H DOSY NMR (600 MHz, $CDCl_3$) of **3b**.

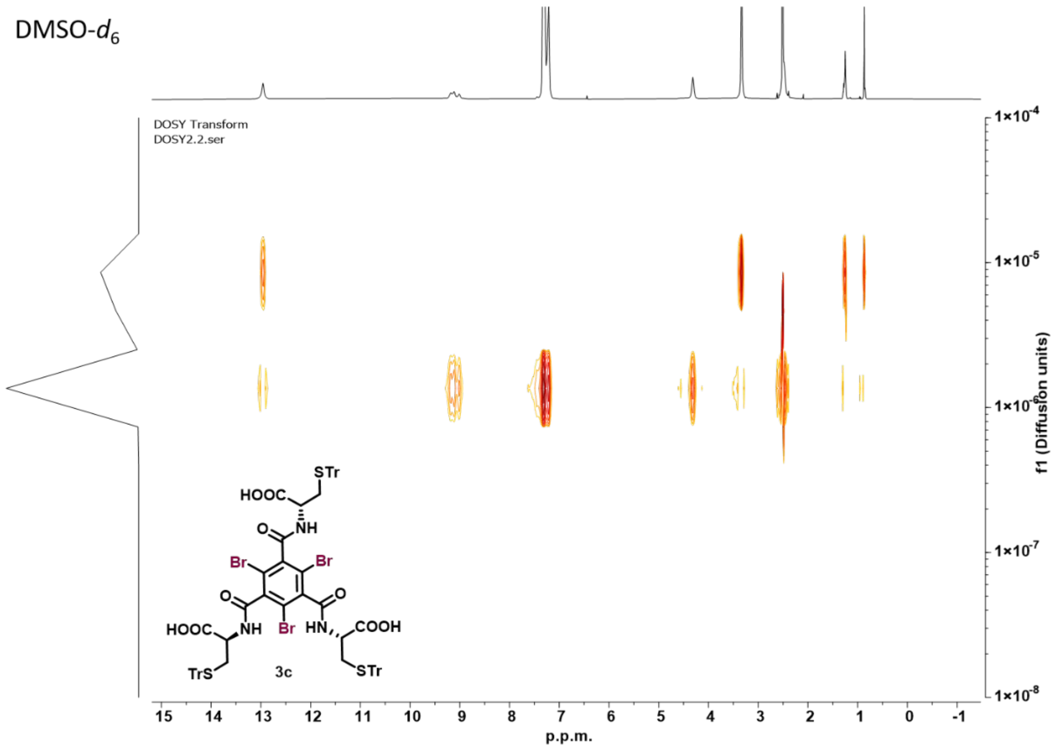

Fig. S37. ^{1}H DOSY NMR (600 MHz, DMSO-d_6) of **3c**.

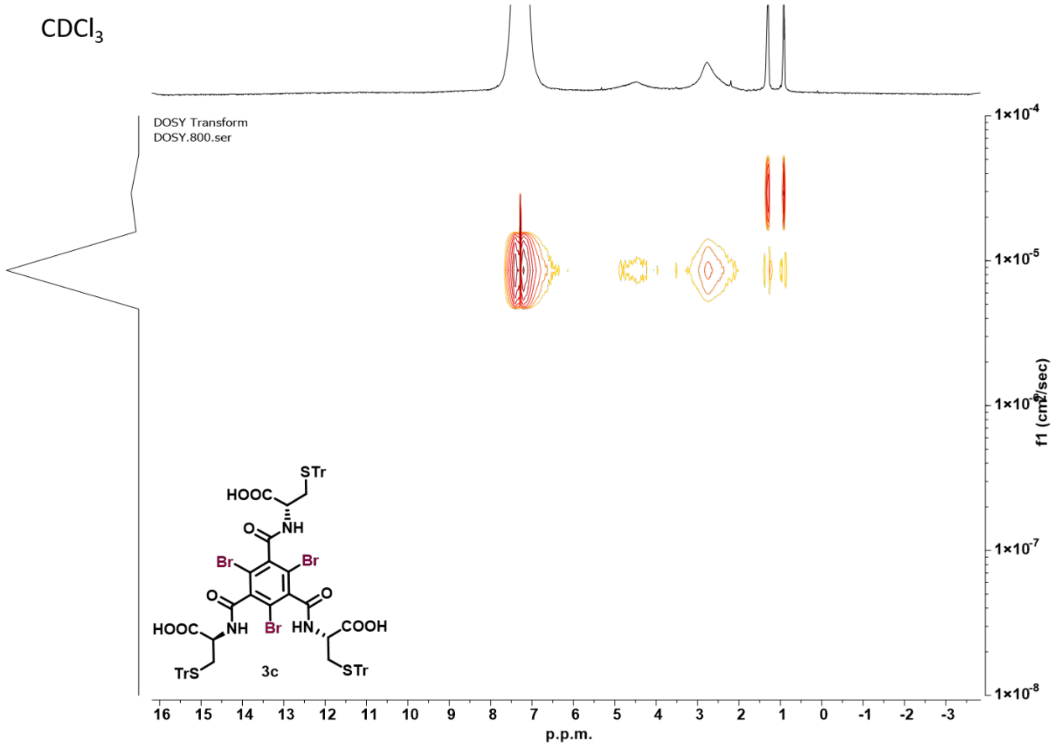

Fig. S38. ^{1}H DOSY NMR (600 MHz, $CDCl_3$) of **3c**.

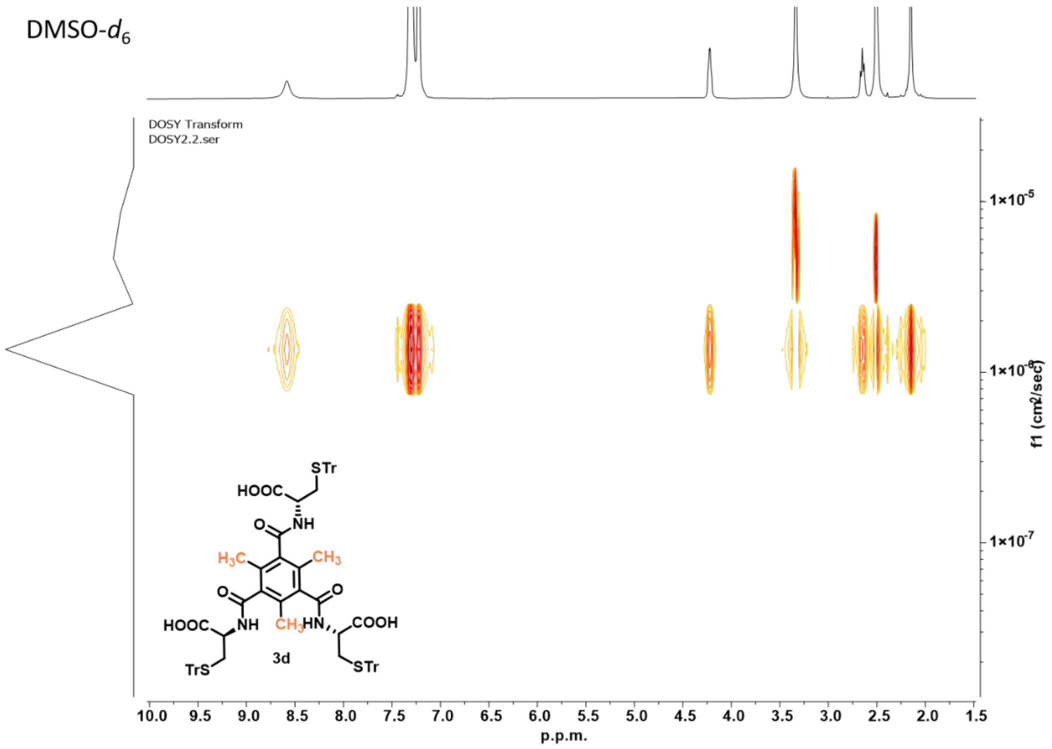

Fig. S39. ^{1}H DOSY NMR (600 MHz, DMSO-d_6) of **3d**.

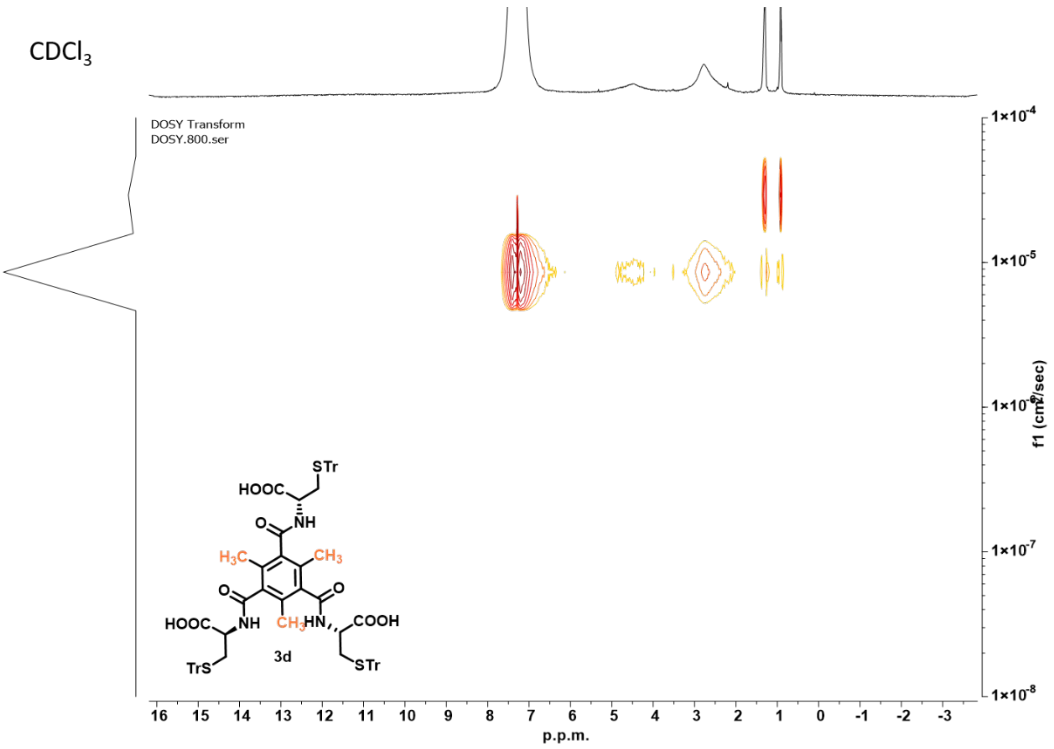

Fig. S40. ^{1}H DOSY NMR (600 MHz, $CDCl_3$) of **3d**.

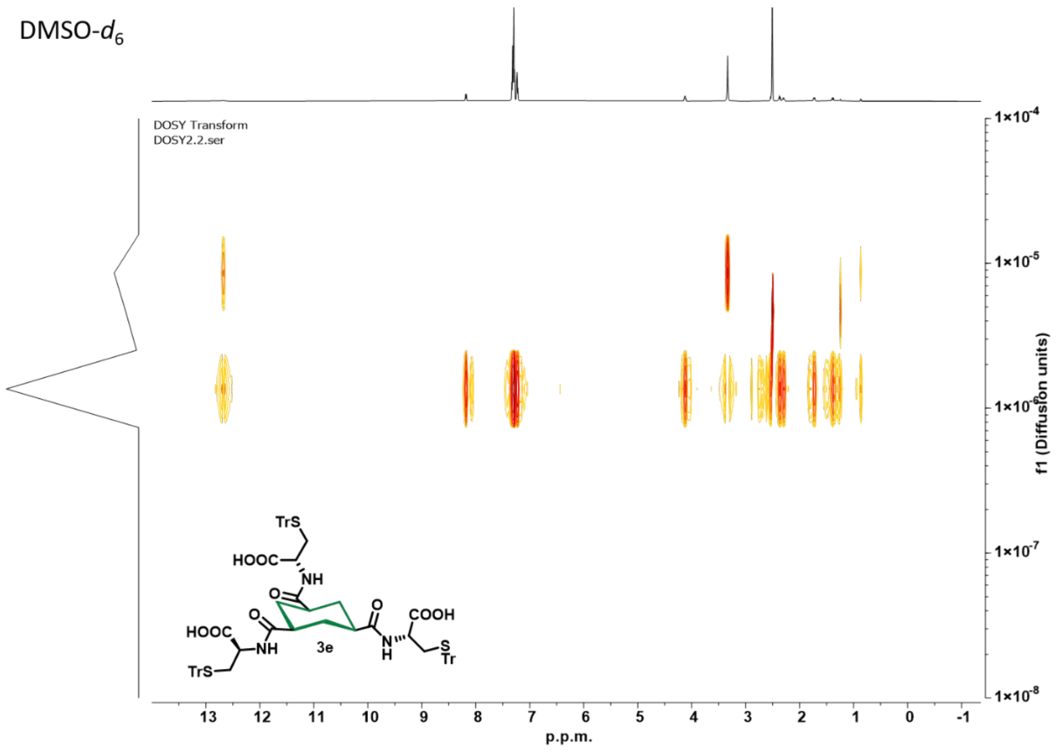

Fig. S41. ^{1}H DOSY NMR (600 MHz, DMSO-d_6) of **3e**.

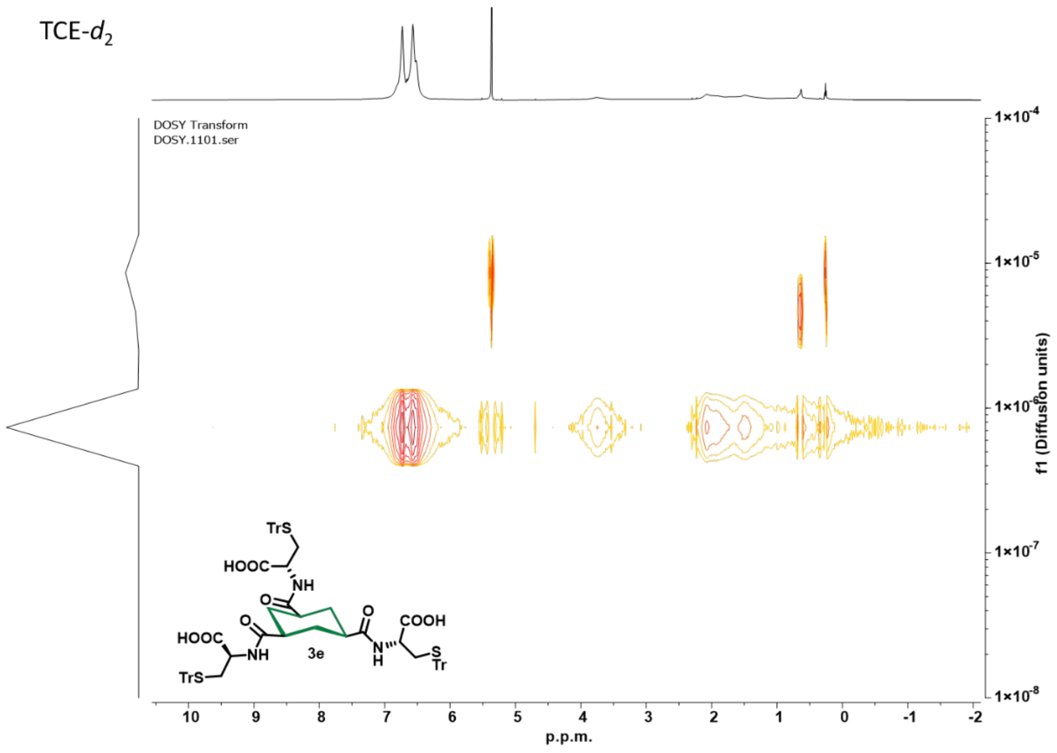

Fig. S42. ^{1}H DOSY NMR (600 MHz, TCE-d_2) of **3e**.

2.7. FT-IR

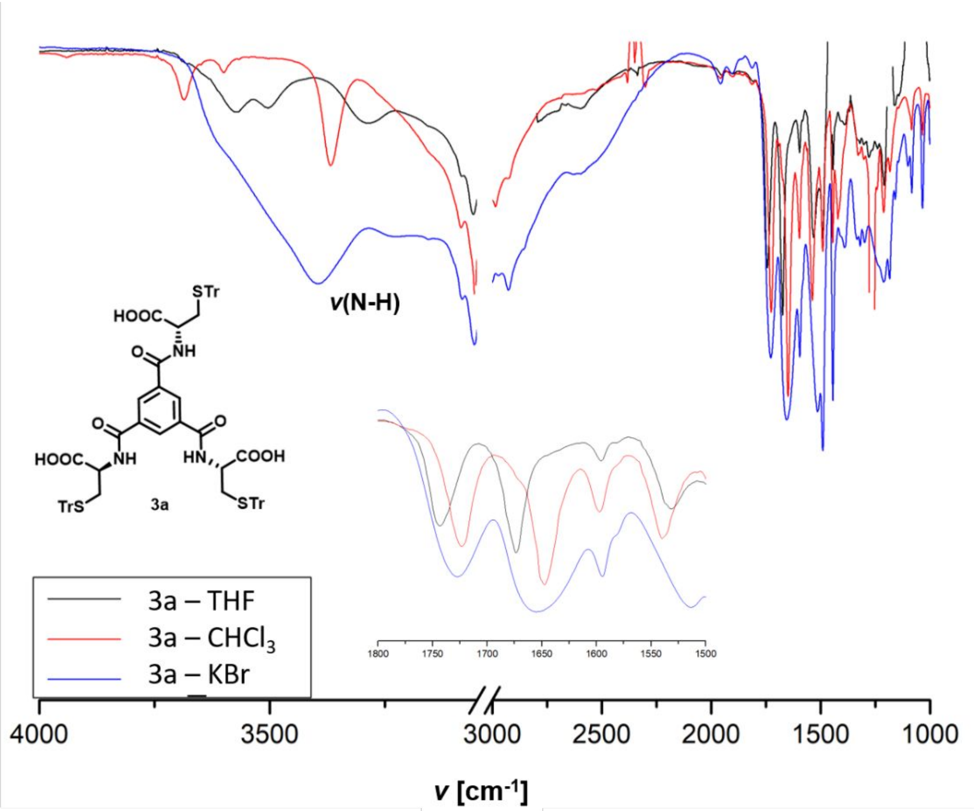

Fig. S43. FT-IR of **3a** in THF, $CHCl_3$ and KBr

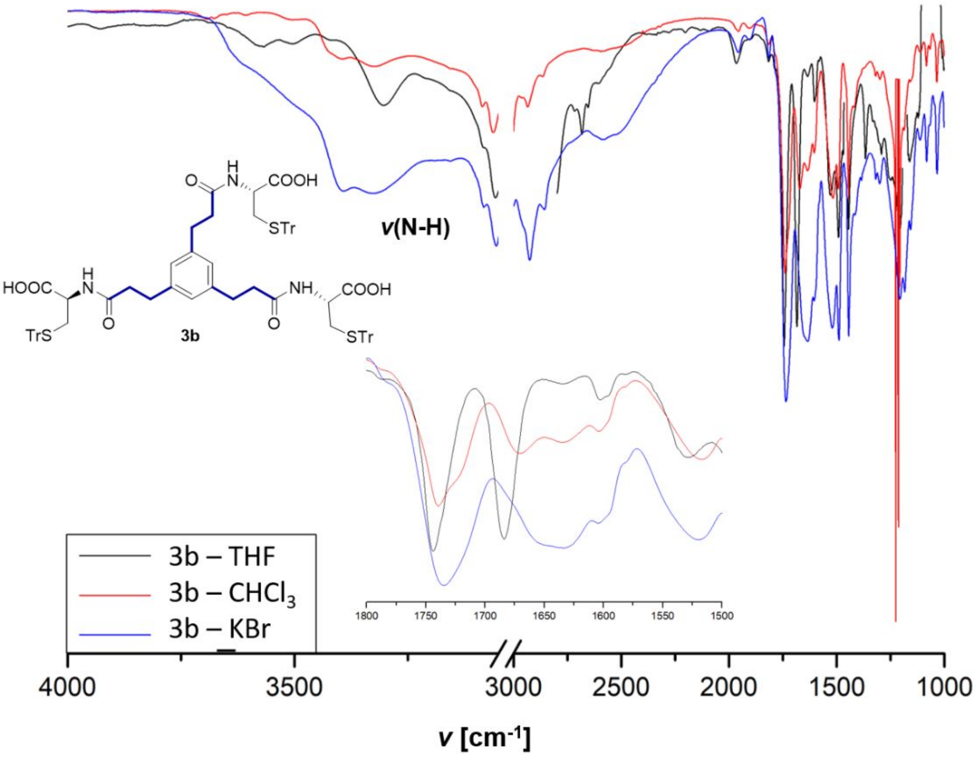

Fig. S44. FT-IR of **3b** in THF, $CHCl_3$ and KBr.

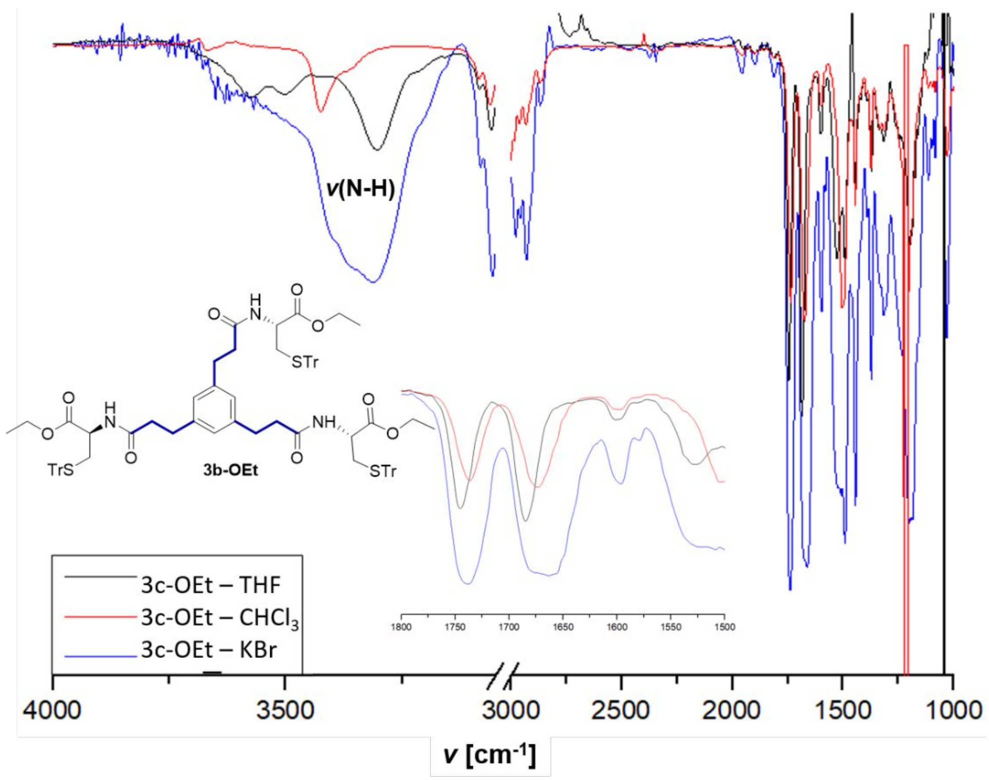

Fig. S45. FT-IR of **3b-OEt** in THF, $CHCl_3$ and KBr.

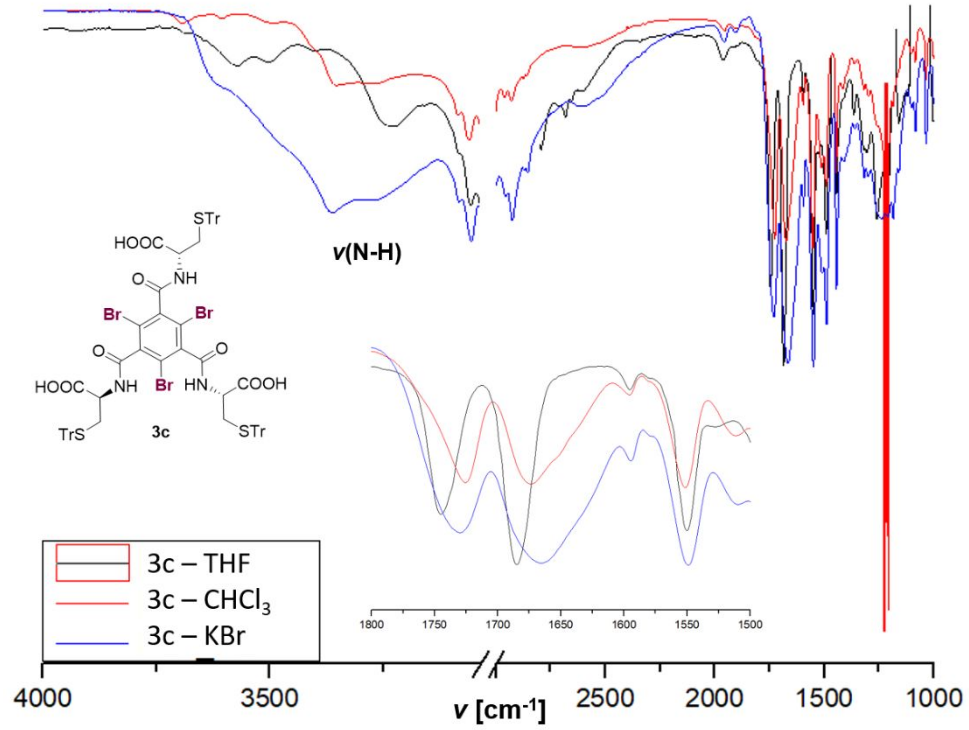

Fig. S46. FT-IR of **3c** in THF, $CHCl_3$ and KBr pellet.

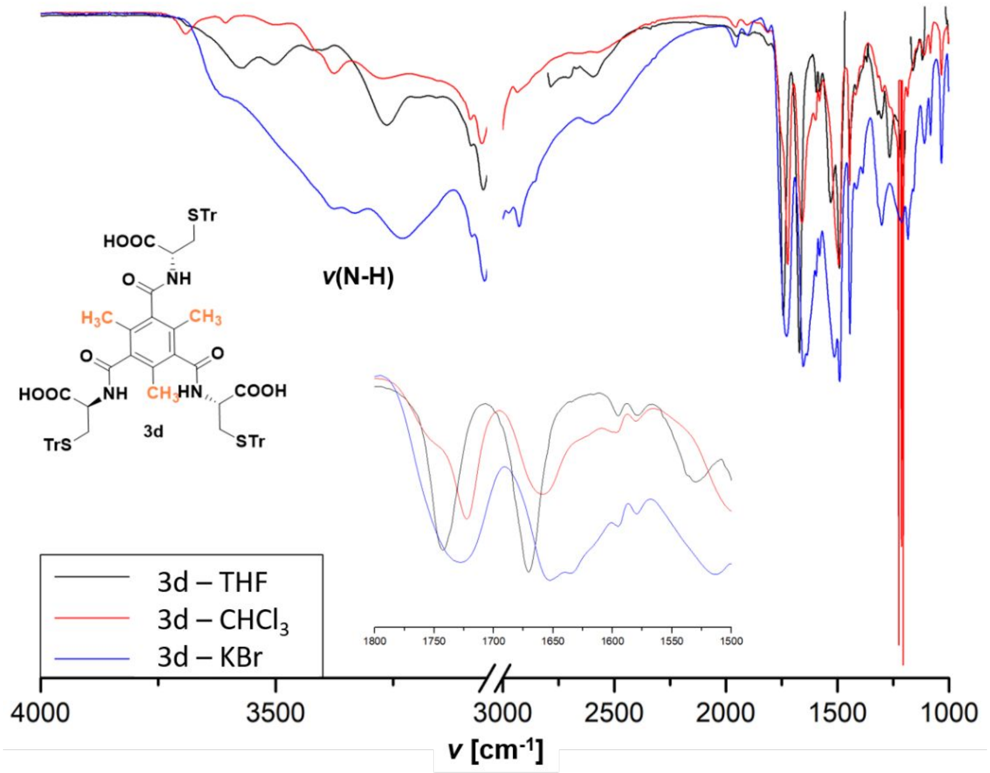

Fig. S47. FT-IR of **3d** in THF, $CHCl_3$ and KBr pellet.

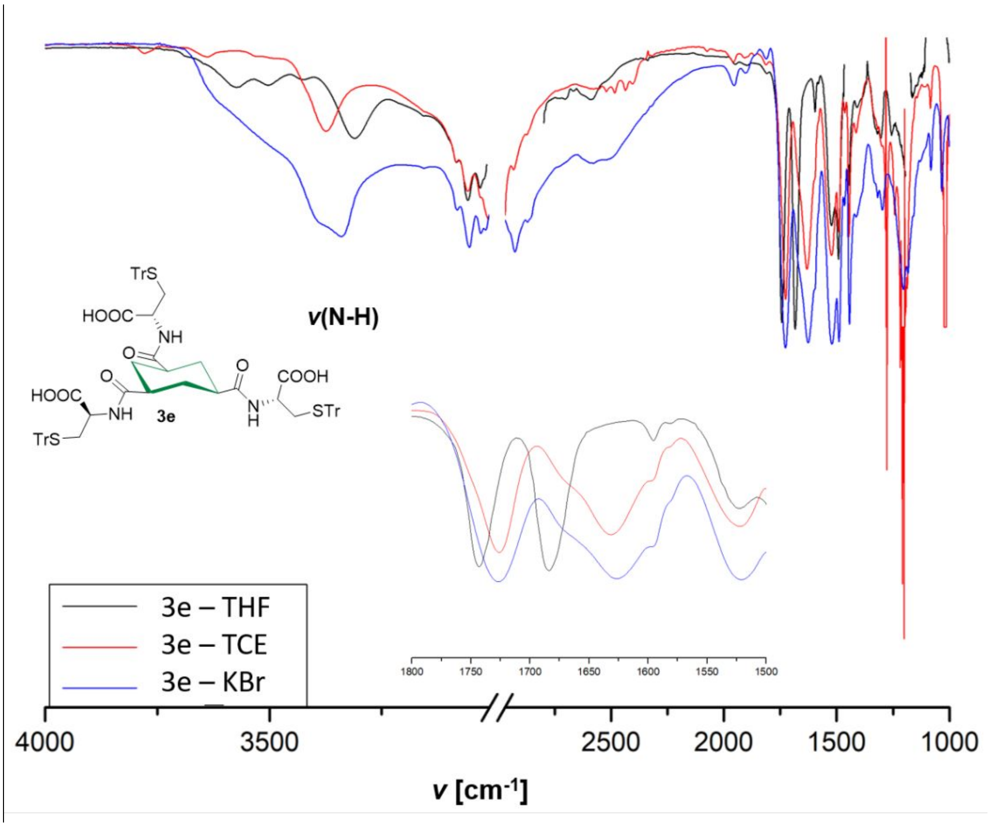

Fig. S48. FT-IR of **3e** in THF, TCE and KBr pellet.

3. Study self-assembly

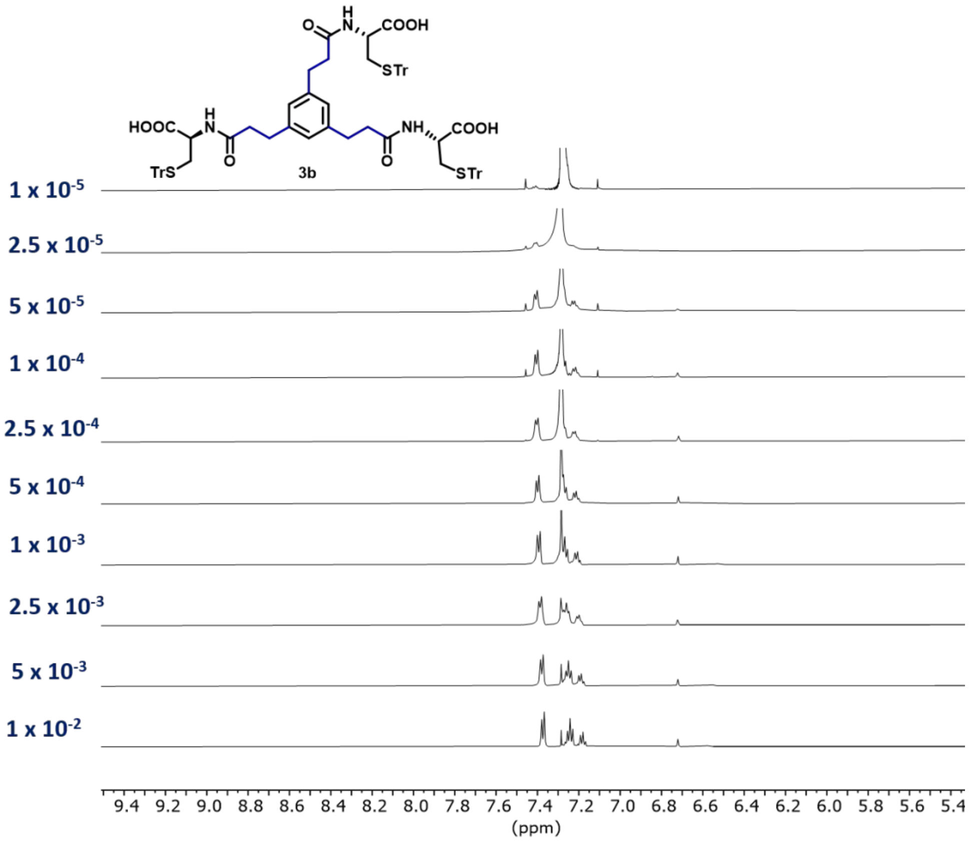

Fig. S49. Concentration-dependent ^{1}H NMR spectra of **3b** in $CDCl_3$.

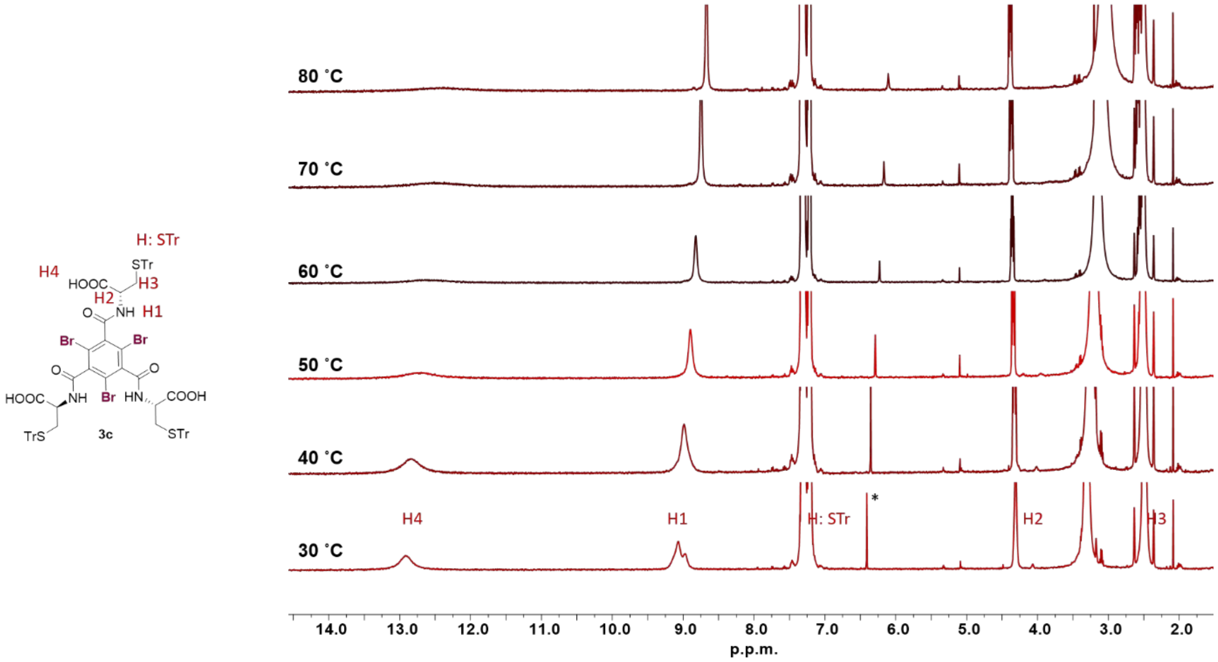

Fig. S50. Variable-temperature ^{1}H NMR spectra of **3c** in DMSO. *DCM

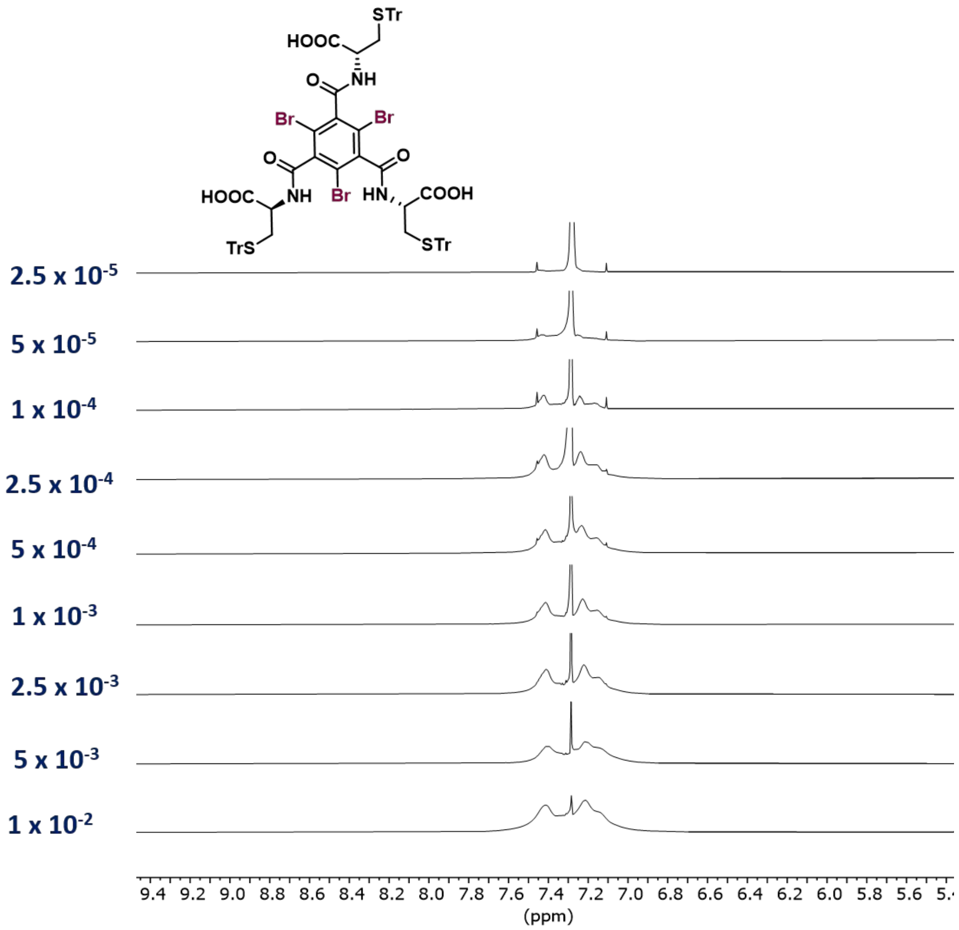

Fig. S51. Concentration-dependent ^{1}H NMR spectra of **3c** in $CDCl_3$.

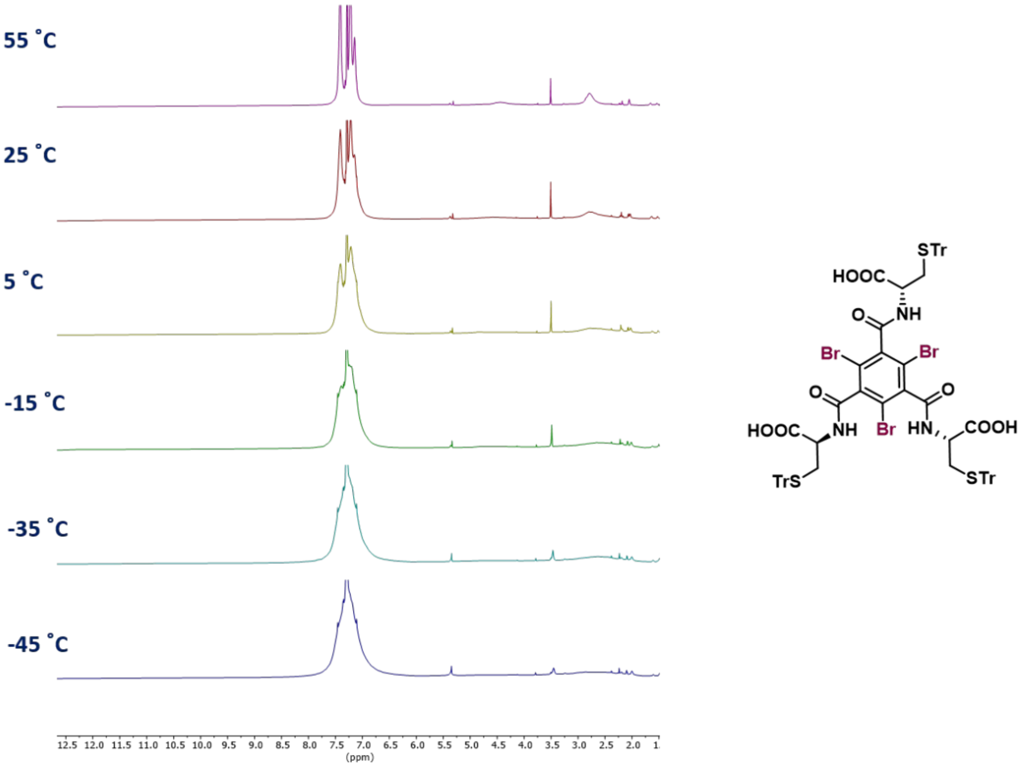

Fig. S52. Variable-temperature ^{1}H NMR (600 MHz) spectra of **3c** in $CDCl_3$.

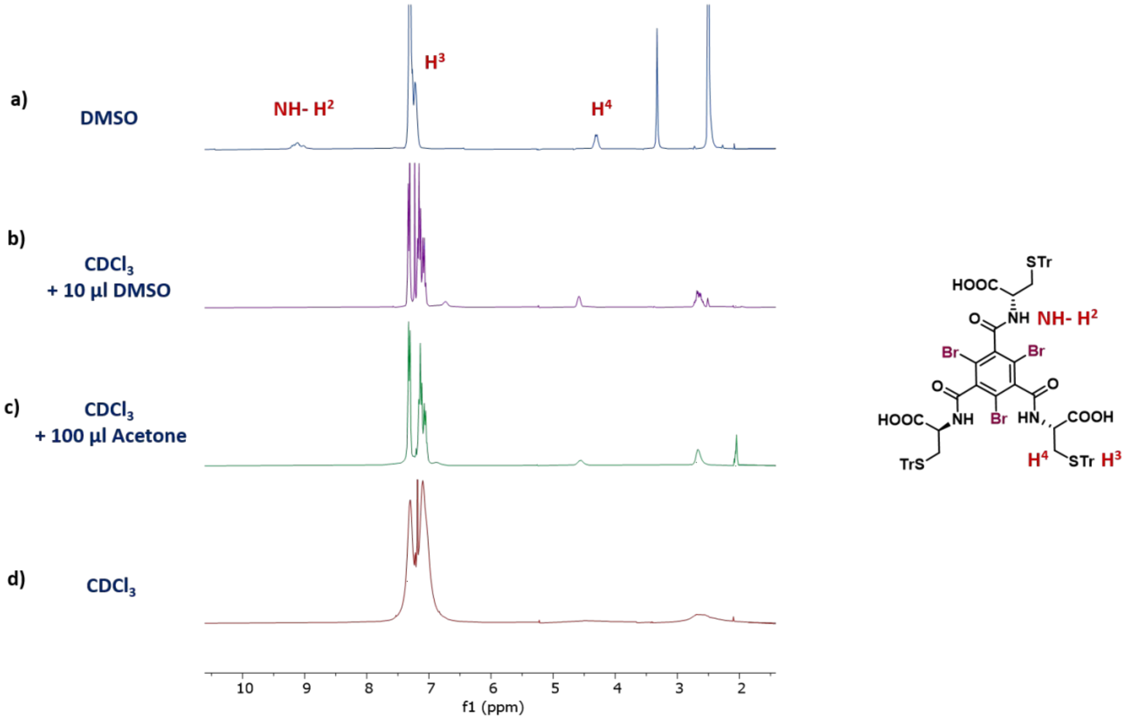

Fig. S53. ^{1}H NMR (600 MHz) of **3c** in a) DMSO-d_6, b) $CDCl_3$ + 10 µl DMSO, c) $CDCl_3$ + 10 µl acetone, d) $CDCl_3$.

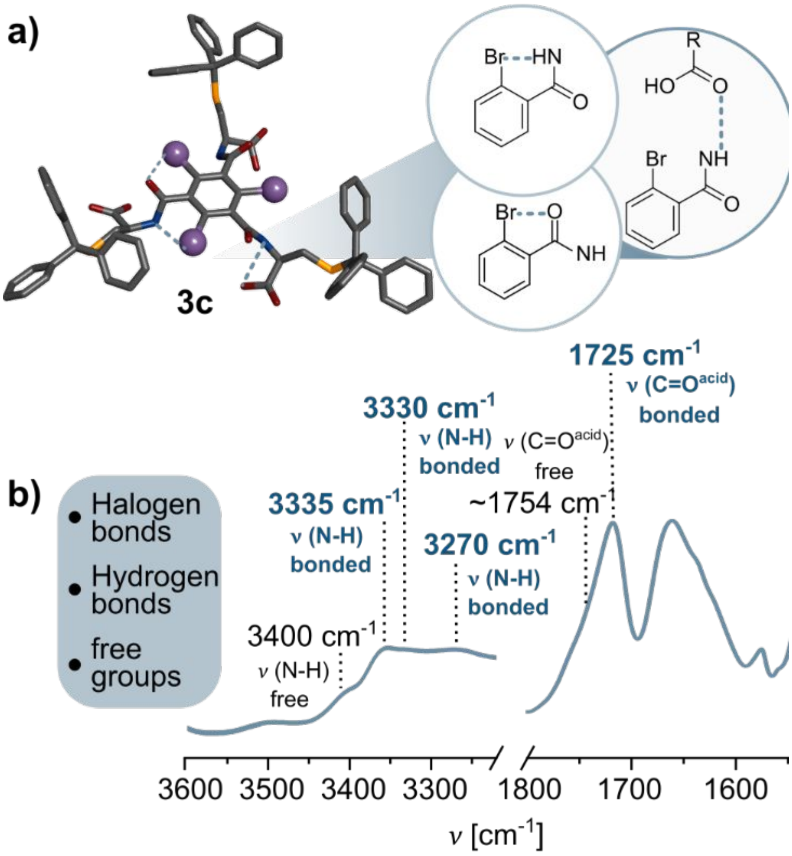

Fig. S54. a) MM2 models of **3c** with marked hydrogen bonds. Some of the H atoms have been omitted for clarity. b) FT-IR characterization of (**3c**) at C= 1.0 × 10^{-2} M and 298 K in $CHCl_3$ with assigned bands. Zoom on the N–H and C=O regions.

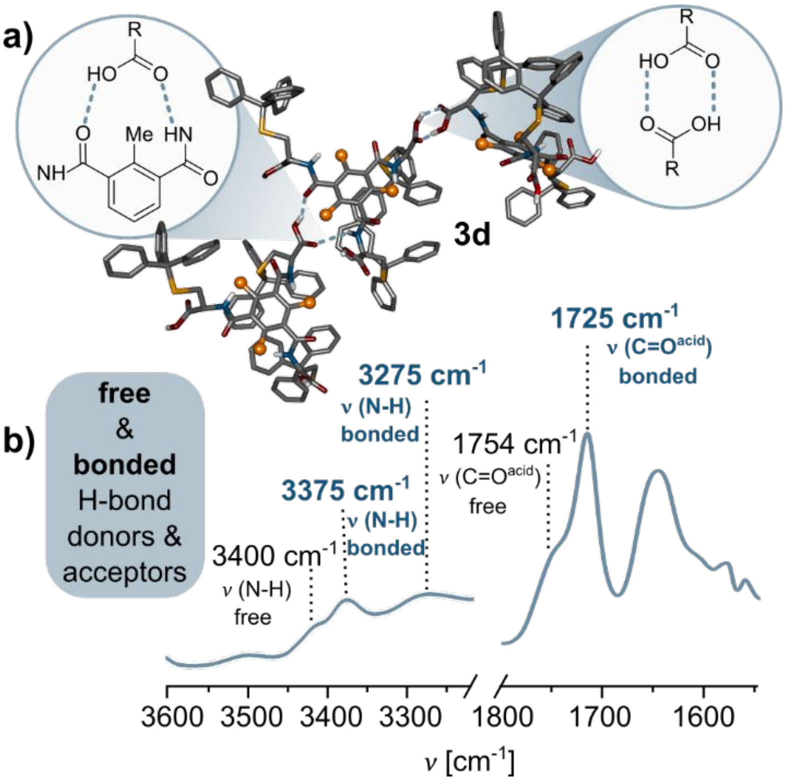

Fig. S55. a) MM2 model of (**3d**) with marked hydrogen bonds. Some of the H atoms have been omitted for clarity. b) FT-IR characterization of (**3d**) at C= 1.0 × 10^{-2} M and 298 K in $CHCl_3$ with assigned bands. Zoom on the N–H and C=O regions.

ORD (optical rotatory dispersion) measurements were performed, confirming the chiral purity of compound 3e, as evidenced by the positive ORD signal across the entire wavelength range of the dispersion (Figure S41).

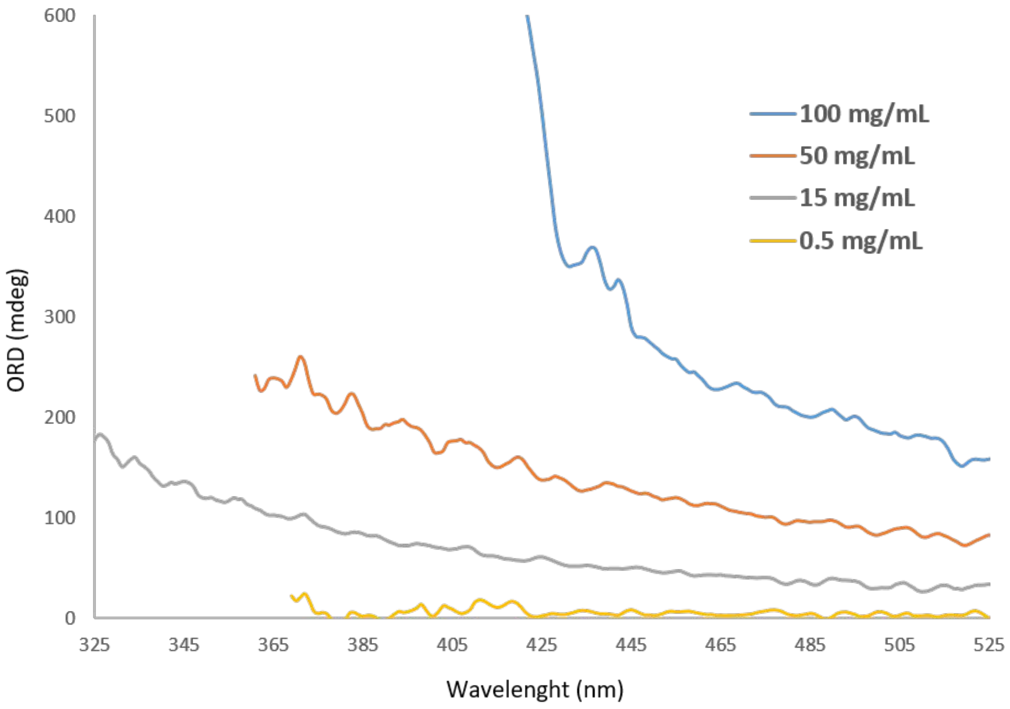

Fig. S56. ORD spectra for **3e** at four concentrations in TCE.

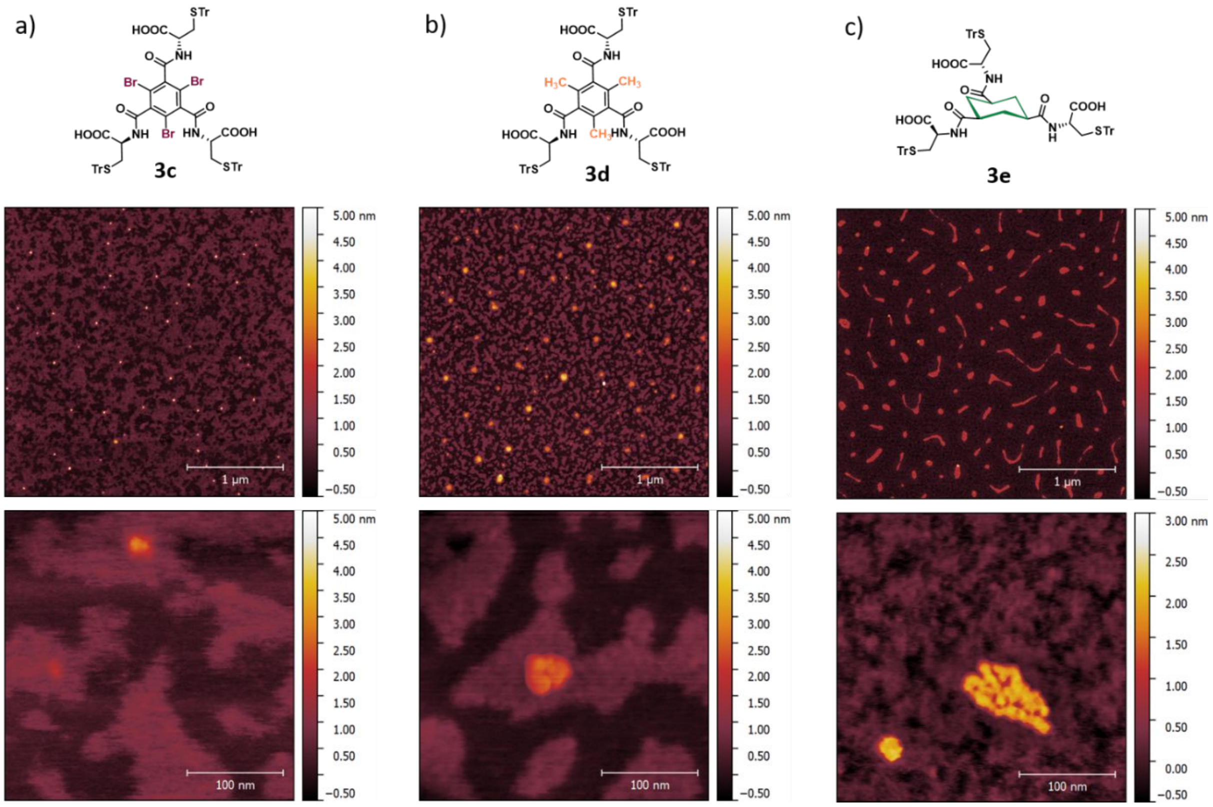

Fig. S57. AFM images of a) **3c** b) **3d** and c) **3e**. $C = 2.5 \times 10^{-4}$ M in TCE.

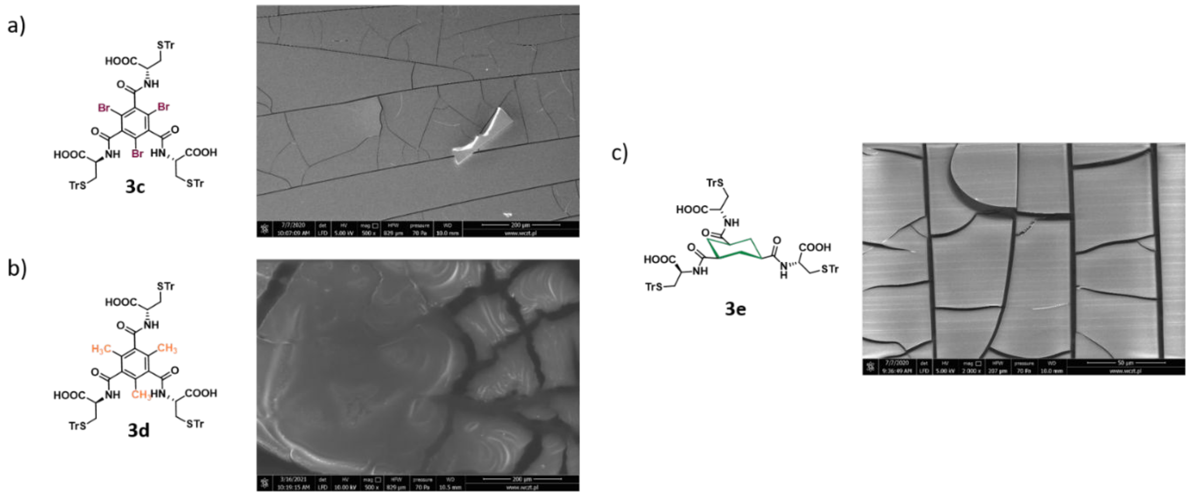

Fig. S58. Scanning electron microscopy (SEM) images of: a) **3c** b) **3d** and c) **3e**. $C = 2.5 \times 10^{-4}$ M in TCE.

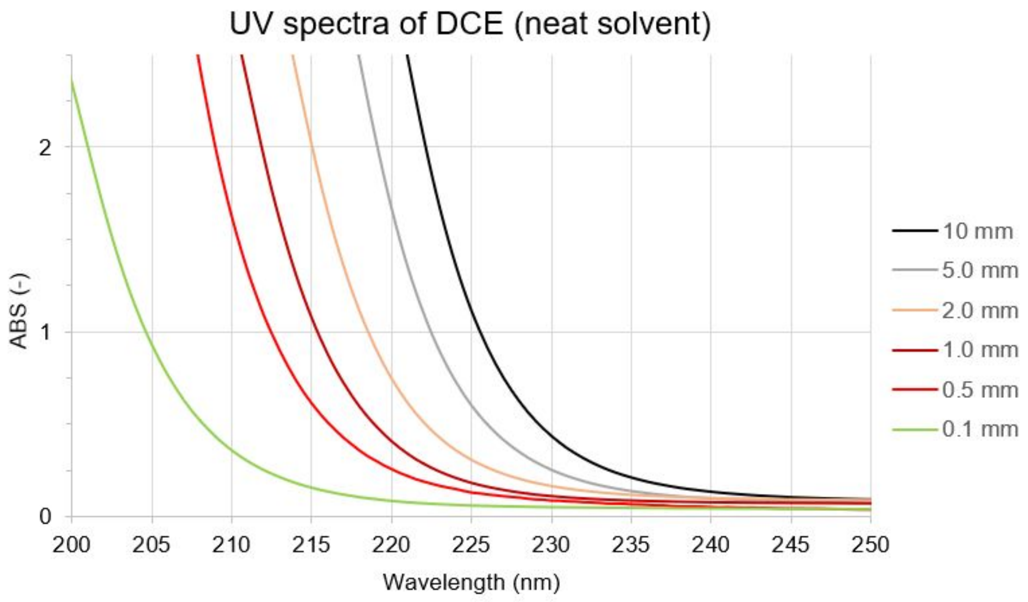

Fig. S59. UV-vis spectra of neat 1,2-dichloroethane (DCE) collected at various pathlengths.

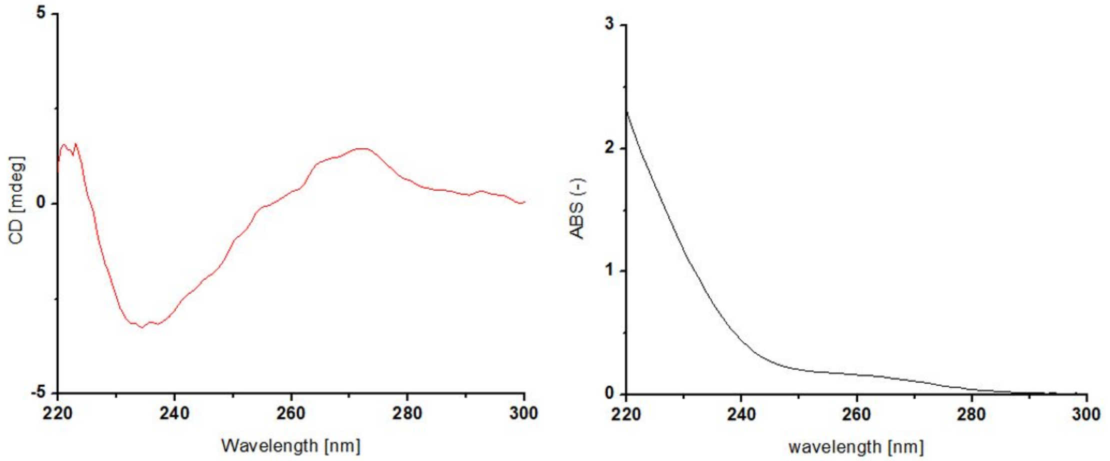

Fig. S60. CD and UV-vis spectra of **3d** recorded in THF (C=2.5 ×10^{-4} M, d=1 mm).

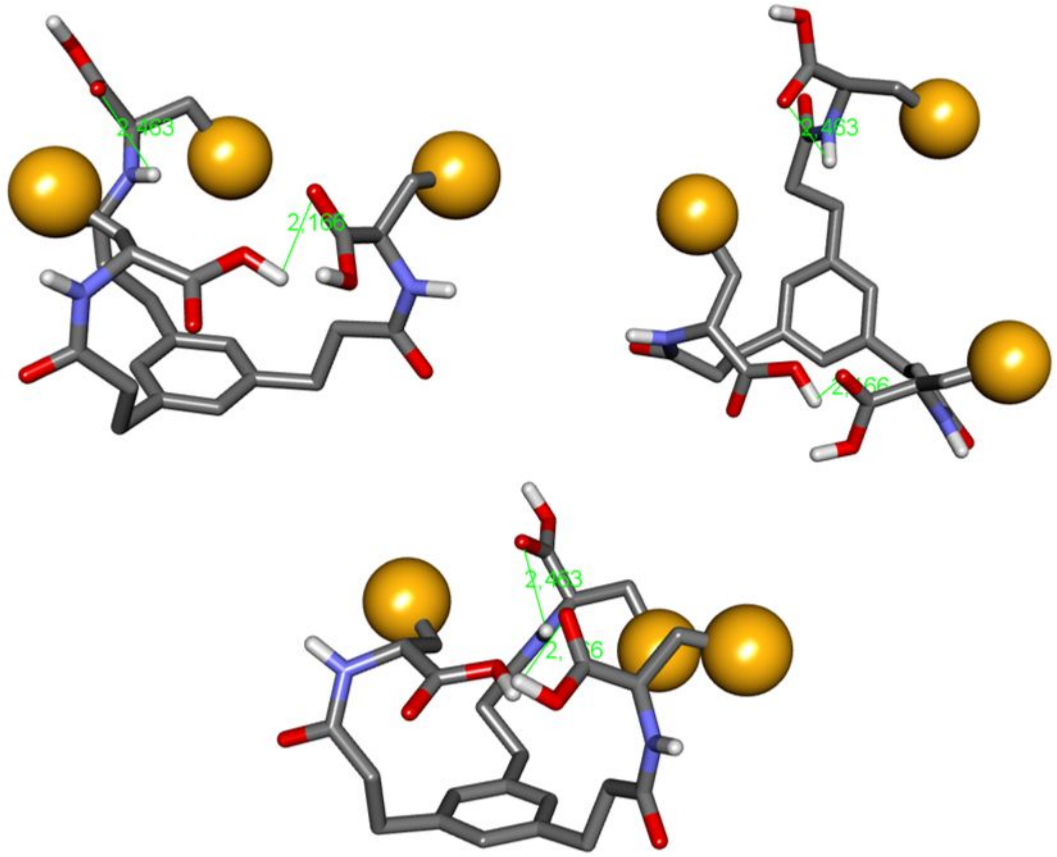

Fig. S61. Different views of the MM2 model of compound **3d** with intramolecular hydrogen bonds highlighted. Some H atoms and the STr group are omitted for clarity.

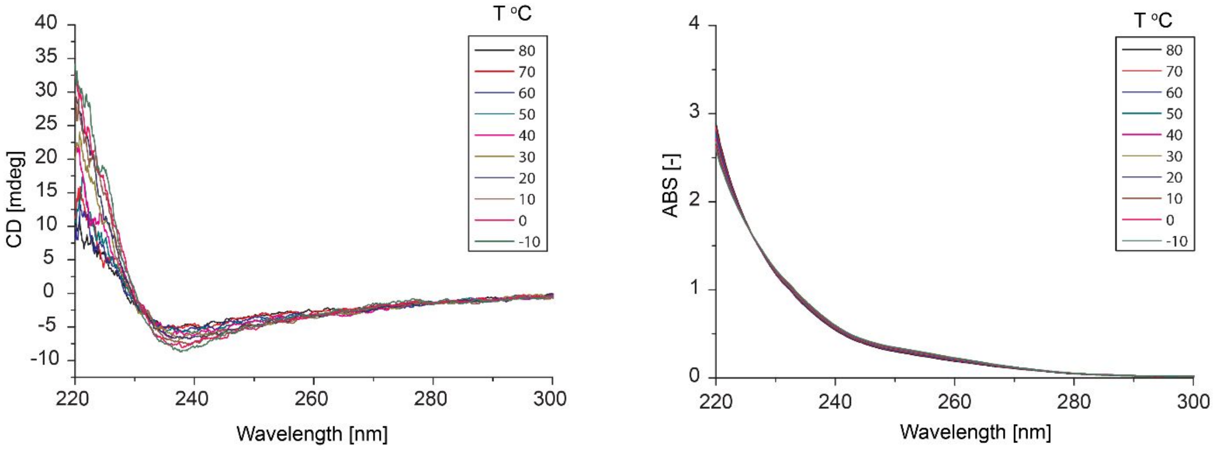

Fig. S61. Variable temperature CD and Variable temperature UV-vis spectra of **3a** in DCE (C=1.0 ×10^{-4} M, d=2 mm).

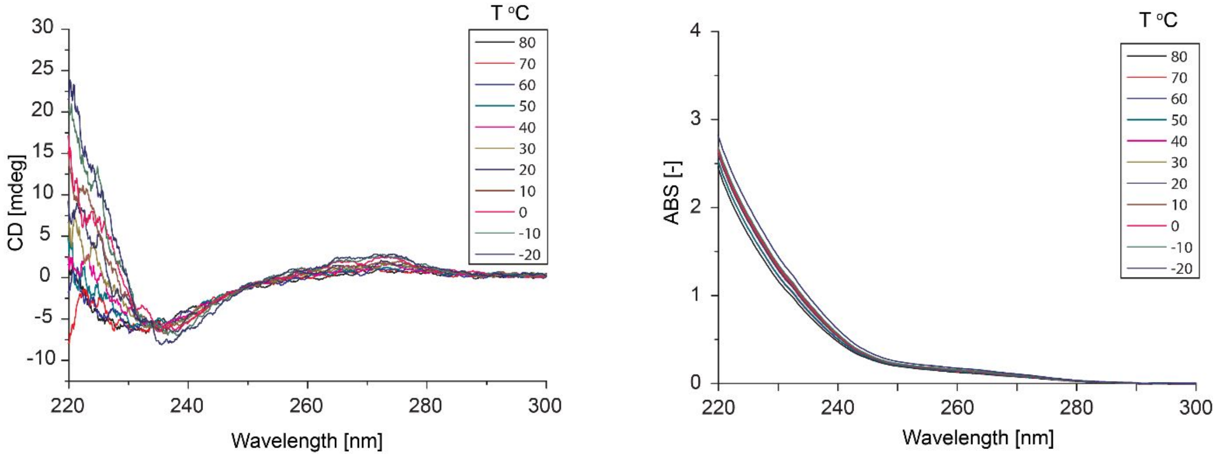

Fig. S62. Variable temperature CD and Variable temperature UV-vis spectra of **3d** in DCE (C= 5.0 ×10^{-4} M, d=0.5 mm).

4. Literature:

[1] D. C. Batesky, M. J. Goldfogel, D. J. Weix, *The Journal of Organic Chemistry* **2017**, *82*, 9931-9936.

[2] aM. W. Majchrzak, J. N. Zobel, D. J. Obradovich, G. A. Peterson, *Organic Preparations and Procedures International* **1997**, *29*, 361-364; bY. Wang, Z.-Q. Wang, L. Yin, *Synthesis* **2023**, *55*, 2228-2240.

[3] G. De Bo, M. A. Y. Gall, M. O. Kitching, S. Kuschel, D. A. Leigh, D. J. Tetlow, J. W. Ward, *Journal of the American Chemical Society* **2017**, *139*, 10875-10879.

[4] D. Bruns, H. Miura, K. P. C. Vollhardt, A. Stanger, *Organic Letters* **2003**, *5*, 549-552.

[5] M. L. Bushey, T.-Q. Nguyen, C. Nuckolls, *Journal of the American Chemical Society* **2003**, *125*, 8264-8269.

[6] S. Hiraoka, T. Yi, M. Shiro, M. Shionoya, *Journal of the American Chemical Society* **2002**, *124*, 14510-14511.

[7] S. V. Kolotuchin, P. A. Thiessen, E. E. Fenlon, S. R. Wilson, C. J. Loweth, S. C. Zimmerman, *Chemistry – A European Journal* **1999**, *5*, 2537-2547.

[8] I. NAVIGEN, **2024**, *TW202438508A*
